# Pathway to Deployment of Gene Drive Mosquitoes as a Potential Biocontrol Tool for Elimination of Malaria in Sub-Saharan Africa: Recommendations of a Scientific Working Group†

**DOI:** 10.4269/ajtmh.18-0083

**Published:** 2018-06

**Authors:** Stephanie James, Frank H. Collins, Philip A. Welkhoff, Claudia Emerson, H. Charles J. Godfray, Michael Gottlieb, Brian Greenwood, Steve W. Lindsay, Charles M. Mbogo, Fredros O. Okumu, Hector Quemada, Moussa Savadogo, Jerome A. Singh, Karen H. Tountas, Yeya T. Touré

**Affiliations:** 1 Foundation for the National Institutes of Health, Bethesda, Maryland;; 2 University of Notre Dame, Notre Dame, Indiana;; 3 Institute for Disease Modeling, Bellevue, Washington;; 4 McMaster University, Hamilton, Canada;; 5 Oxford University, Oxford, United Kingdom;; 6 London School of Hygiene & Tropical Medicine, London, United Kingdom;; 7 Durham University, Durham, United Kingdom;; 8 Kenya Medical Research Institute, Nairobi, Kenya;; 9 Ifakara Health Institute, Ifakara, Tanzania;; 10 University of Glasgow, Glasgow, Scotland;; 11 University of the Witwatersrand, Johannesburg, South Africa;; 12 Donald Danforth Plant Science Center, Saint Louis, Missouri;; 13 New Partnership for Africa’s Development, Ouagadougou, Burkina Faso;; 14 Centre for the AIDS Programme of Research in South Africa, Durban, KwaZulu-Natal, South Africa;; 15 University of Sciences, Techniques and Technologies of Bamako, Bamako, Mali

## Abstract

Gene drive technology offers the promise for a high-impact, cost-effective, and durable method to control malaria transmission that would make a significant contribution to elimination. Gene drive systems, such as those based on clustered regularly interspaced short palindromic repeats (CRISPR)/CRISPR associated protein, have the potential to spread beneficial traits through interbreeding populations of malaria mosquitoes. However, the characteristics of this technology have raised concerns that necessitate careful consideration of the product development pathway. A multidisciplinary working group considered the implications of low-threshold gene drive systems on the development pathway described in the World Health Organization *Guidance Framework for testing genetically modified (GM) mosquitoes*, focusing on reduction of malaria transmission by *Anopheles gambiae* s.l. mosquitoes in Africa as a case study. The group developed recommendations for the safe and ethical testing of gene drive mosquitoes, drawing on prior experience with other vector control tools, GM organisms, and biocontrol agents. These recommendations are organized according to a testing plan that seeks to maximize safety by incrementally increasing the degree of human and environmental exposure to the investigational product. As with biocontrol agents, emphasis is placed on safety evaluation at the end of physically confined laboratory testing as a major decision point for whether to enter field testing. Progression through the testing pathway is based on fulfillment of safety and efficacy criteria, and is subject to regulatory and ethical approvals, as well as social acceptance. The working group identified several resources that were considered important to support responsible field testing of gene drive mosquitoes.

## INTRODUCTION

**Figure 1:**
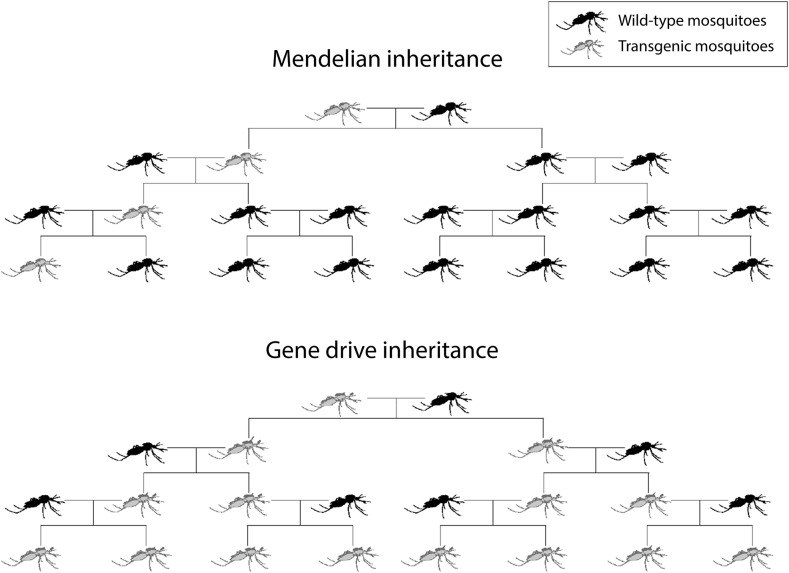
Spread of novel traits by gene drive vs. Mendelian inheritance.

Mosquitoes modified with gene drive systems are being proposed as new tools that will complement current practices aimed at reducing or preventing transmission of vector-borne diseases such as malaria. Gene drive systems have the potential to spread new genetic traits through interbreeding populations of malaria mosquitoes from low initial introductions (Figure 1), and the transgenic construct could persist in those mosquitoes indefinitely or until the target mosquito population is locally eliminated. Having observed naturally occurring drive mechanisms in insects and other organisms, scientists speculated for decades about how these mechanisms could be harnessed to insert beneficial traits into a population of vector mosquitoes to create a high-impact, low-cost, sustainable tool for controlling disease transmission.^1^ With the advent of new molecular tools for modifying mosquitoes,^2^ a mechanism was envisioned to use synthetic genes with the capability of spreading in populations, even if they confer a fitness cost (driving transgenes). The envisioned goal for applying this technology is to reduce or eliminate vector mosquito populations or, alternatively, to render them less competent to transmit pathogens. Either of these outcomes should contribute to disease reduction. However, the characteristics that make gene drive technology so attractive as a cost-effective and durable vector control tool raise questions about possible adverse effects on human or animal health or the environment that must be seriously considered in product development.

Several mechanisms are being examined to achieve gene drive.^3,4^ Until recently, the attempted methods either did not work in mosquitoes or were difficult to engineer^5,6^; however, discovery of the clustered regularly interspaced short palindromic repeats (CRISPR)/CRISPR associated protein 9 (Cas9) system for gene editing has provided a widely accessible and versatile molecular tool for creating driving transgenes.^7^ The use of CRISPR/Cas9 in mosquitoes follows from an idea, conceived in 2003, that naturally occurring genes producing homing endonuclease enzymes that target and cut specific deoxyribonucleic acid (DNA) sequences could be used to create gene drive.^8^ Conceptually, constructs incorporating the CRISPR/Cas9 system spread in the same way as these natural endonuclease genes, but the easy manipulation of the guide ribonucleic acid (RNA) that specifically selects the site where the chromosome cut occurs allows for the targeting of a greater range of gene sequences. Computational modeling based on other gene drive systems suggests that the type of drive that can be achieved with the CRISPR/Cas9 system can be so effective that release of low numbers of modified mosquitoes into the environment could result in establishment of the genetic modification in the natural interbreeding population (P. A. Welkhoff, personal communication).^9,10^ Although still in the process of being optimized, such mosquitoes have already been developed in the laboratory with the ultimate intent of testing in the field.^11,12^ Computer simulations and population genetic analyses suggest that gene drive strategies for reducing or modifying the population of vector mosquitoes both have the potential to provide a transformative new tool for conquering malaria and to make a valuable contribution toward the elimination, and ultimate eradication, of this disease.^13,14^

In 2014, the World Health Organization (WHO) released the *Guidance Framework for testing genetically modified (GM) mosquitoes* (WHO Guidance Framework) that describes a phased testing pathway and best practices for evaluating GM mosquitoes (GMM) intended as public health tools.^15^ The proposed product development pathway moves from physically confined (also termed contained) studies in the laboratory and insectary (Phase 1) to small-scale physically and/or ecologically confined field-testing (Phase 2). Early small-scale releases in Phase 2 are intended to allow observation of the behavior of GMM in natural environments, and thus assessment of entomological endpoints of efficacy, under conditions that would minimize risk to the environment and/or human health. Contingent on satisfactory results from confined testing, the WHO Guidance Framework advocates proceeding to a series of staged open release trials of increasing size, duration, and complexity (Phase 3).^15^ These trials assess performance under various conditions, such as different levels of pathogen transmission, seasonal variations in mosquito density, or presence of other disease vectors in the region. Larger trials in this phase allow measurement of the impact of GMM on infection and/or disease in human populations, in addition to entomological endpoints. Following successful completion of Phase 3, national authorities will determine whether to move a specific GMM product into application as a malaria control tool (Phase 4), which would include ongoing surveillance of the effectiveness of the product under operational conditions, accompanied by monitoring of safety under diverse conditions of use.

New low-threshold‡ gene drive technologies, such as those using the CRISPR/Cas9 system, have broad implications at multiple phases of the recommended WHO Guidance Framework testing pathway because of the potential to be self-sustaining,^15^ that is, to spread a genetic modification through the local mosquito population, and for that modification to become established and to persist. Recognizing both the benefits and risks accompanying this new technology, there have been calls for additional guidance and oversight before any field-testing begins.^7,16,17^ The recommendations provided here represent the response of a multidisciplinary working group that comprised international experts in mosquito research (including, but not limited to, molecular entomologists and individuals with field experience in vector ecology and control), as well as experts in containment/quarantine of exotic arthropods, mathematical modeling, epidemiology, clinical trial design, statistics, ethics, regulatory science, and policy (Box 1). Working group members considered whether mosquitoes modified with low-threshold gene drive could be developed appropriately and used against malaria, and, if so, the resources and activities needed to ensure their safe and efficient field-testing and implementation. These consensus recommendations build primarily on existing guidance provided by the WHO Guidance Framework,^15^ but also take into account recommendations from the report of the National Academies of Science, Engineering, and Medicine (NASEM) *Gene Drives on the Horizon: Advancing Science, Navigating Uncertainty, and Aligning Research with Public Values* (NASEM report),^18^ which considered the broader public health, conservation, and agricultural potential of gene drive technology, as well as widely accepted guiding principles for sponsors and supporters of gene drive research.^19^ The recommendations presented here attempt to envision the entire development pathway for gene drive mosquitoes, from discovery research to implementation, to provide a basis for establishment of standards of best practice before the initiation of any field trials. Like the WHO Guidance Framework^15^ and NASEM report,^18^ they are intended to inform decision-making by researchers, funders, regulators, and policy-makers. It is anticipated that these recommendations will be revised and refined as more experience with gene drive technologies is accumulated.

Box 1 **Working group composition**

**Core Working Group Members:** participated in all working group activities and authored the recommendations

Frank H. Collins, University of Notre Dame; Philip A. Welkhoff, Institute for Disease Modeling; Claudia Emerson, McMaster University; H. Charles J. Godfray, Oxford University; Brian Greenwood, London School of Hygiene & Tropical Medicine; Steve W. Lindsay, Durham University; Charles M. Mbogo, Kenya Medical Research Institute; Fredros O. Okumu, Ifakara Health Institute, University of Glasgow, University of the Witwatersrand; Hector Quemada, Donald Danforth Plant Science Center; Moussa Savadogo, New Partnership for Africa’s Development (NEPAD); Jerome A. Singh, Center for the AIDS Program of Research in South Africa; Yeya T. Touré, University of Sciences, Techniques and Technologies of Bamako

**Ad Hoc Working Group Participants:** attended specific working group meetings as appropriate to areas of expertise and provided comments

Aggrey Ambali, NEPAD; Mark Benedict, Foundation for the Centers for Disease Control and Prevention; Christophe Boete,* Institut pour Recherche pour le Développement; Catherine Bourgouin,* Institut Pasteur; Paul De Barro, The Commonwealth Scientific and Industrial Research Organisation; Abdoulaye Diabate, Institut de Recherche en Science de la Santé/Center Muraz; Azra Ghani, Imperial College London; Fred Gould, North Carolina State University; Lee Hall, US National Institute of Allergy and Infectious Diseases; Steve Higgs, Kansas State University; Immo Kleinschmidt, London School of Hygiene & Tropical Medicine; Greg Lanzaro, University of California, Davis; Christian Lengeler, Swiss Tropical and Public Health Institute; Jo Lines, London School of Hygiene & Tropical Medicine; David Malone, Innovative Vector Control Consortium; Kevin Marsh, University of Oxford; Leonard Mboera, National Institute for Medical Research Tanzania; Abraham Mnzava, African Leaders Malaria Alliance; Scott O’Neill, Monash University; Seth Owusu-Agyei, University of Health & Allied Sciences; Malla Rao, US National Institute of Allergy and Infectious Diseases; Larry Slutsker, Program for Appropriate Technology in Health; Willy Tonui, National Biosafety Authority (NBA) Kenya; Kenneth Vernick, Institut Pasteur

**Contributors:** provided written or verbal comments or information for working group consideration

Adam Bennett, University of California, San Francisco; Austin Burt, Imperial College, London; Nora Besansky, Notre Dame University; Lorna Clark, Imperial College London; George Christophides, Imperial College London; Andrea Crisanti, Imperial College London; Anthony James, University of California, Irvine; John Marshall, University of California, Berkeley; Tony Nolan, Imperial College, London; Nikolai Windbichler, Imperial College, London

**Observers:** attended one of more working group meetings

Anne Cheever, Booz Allen Hamilton and Contractor support to the Defense Advanced Research Projects Agency; Adriana Costero-Saint Denis, US National Institute of Allergy and Infectious Diseases; Anna Drexler, World Health Organization (WHO), Florence Fouque, WHO; Fil Randazzo, Bill & Melinda Gates Foundation; Mike Reddy, Bill & Melinda Gates Foundation; Emmanuel Temu, WHO; Raman Velayudhan, WHO; Renee Wegrzyn, Defense Advanced Research Projects Agency

* Invited at the recommendation of WHO observers

The investigational product for these recommendations is considered to be the transgenic mosquito carrying a low-threshold gene drive system (for convenience, herein referred to as a gene drive mosquito). Over the course of three face-to-face meetings, with ongoing discussions between each meeting, the working group systematically examined how utilizing low-threshold gene drive might influence the planning and conduct of each testing phase described in the WHO Guidance Framework.^15^ This report does not attempt to summarize the detailed information contained within the WHO Guidance Framework, which was accepted by the working group as the foundation for the additional considerations related here. Readers are encouraged to consult the WHO Guidance Framework for underlying information on efficacy and biosafety testing, ethics, public engagement, and regulatory issues relevant to GMM.^15^ The working group envisioned that these recommendations will be used as a companion to the earlier WHO guidance.

### Scope and rationale.

To focus the discussions, the working group concentrated on the example of malaria transmission in Africa by mosquitoes of the *Anopheles gambiae* complex^20^ (see Box 2).

Box 2 **The *An. gambiae* complex**

The *An. gambiae* complex (also known as *An. gambiae* sensu lato [s.l., in the broad sense]), which includes some of the most important and efficient vectors of malaria in sub-Saharan Africa, consists of eight named sibling species that are difficult to distinguish morphologically but can be identified using molecular methods:

 *An. gambiae* sensu stricto (s.s. in the strict sense)

 Anopheles amharicus

 *Anopheles arabiensis*

 *Anopheles bwambae*

 *Anopheles coluzzii*

 *Anopheles melas*

 *Anopheles merus*

 *Anopheles quadriannulatus*

The individual species exhibit distinct behavioral and ecological preferences. As examples, *An. gambiae* s.s. and *An. coluzzii*, which are closely related, feed almost exclusively on humans (anthropophilic), whereas *An. quadriannulatus* takes its blood meal from animals (zoophilic). *Anopheles melas* and *An. merus* can breed in salt water, whereas *An. gambiae* and the other species breed in fresh water. *Anopheles quadriannulatus* is not considered to be a malaria vector. Although these species are considered to be reproductively isolated, there is evidence of interbreeding between some of them. Patterns of introgression between *An. arabiensis* and *An. gambiae*/*An. coluzzii*, and between *An. merus* and *An. quadriannulatus*, are similar across their geographic range.^21^

Consideration must, therefore, be given to the diversity of members of this complex present at field testing sites and to whether the gene sequence targeted by gene drive constructs is present in more than one species. Nontarget *Anopheles* species should be examined for the extent of gene flow between sibling species and the potential effects of any genetic transfer events.

It was assumed that the transformation event would be performed in *An. gambiae* s.s. and later transferred to sibling species by introgression in the laboratory or by natural hybridization in the field.

*Anopheles gambiae* s.l. mosquitoes are reported only on the African continent.^22^ This geographic limitation is an important consideration in evaluating the potential spread of gene drive approaches targeting these mosquitoes. Although *An. gambiae* s.s. and sibling species *An. coluzzi* and *An. arabiensis* are major malaria vectors in sub-Saharan Africa, the working group recognized that other *Anopheles* species (notably *Anopheles funestus*) also transmit malaria, and may, in certain situations, contribute a significant proportion of the residual transmission.^22,23^ Products directed at these mosquitoes also will be required for malaria elimination.^24^

Because of massive deployment of currently available malaria control tools, *Plasmodium falciparum* infection prevalence in endemic Africa halved and the incidence of clinical disease fell by 40% between 2000 and 2015.^25^ Yet, residual levels of transmission still persist even in places where coverage with existing interventions is already very high.^26^ According to the most recent World Malaria Report 2017,^26^ despite best control efforts undertaken to date, there were 216 (95% confidence interval = 196–263) million cases of malaria and an estimated 445,000 deaths from malaria in 2016, with 90% of cases and deaths occurring in sub-Saharan Africa and with a leveling off in the recent decline in malaria mortality. In 2015, malaria killed an estimated 303,000 children under the age of 5 years globally, and 96% of these deaths occurred in the African region.^26,27^ Although the African region has shown considerable recent progress, malaria remains stubbornly persistent in some areas and is increasing in others^26^; the substantial progress that has been made is fragile and is threatened by insecticide resistance,^28^ changes in vector behavior, resistance to antimalarial therapeutics, and high ongoing costs of malaria control (estimated at over $6 billion per year to meet the 2020 target for reduction in malaria prevalence,^26,29–31^ with over half the costs going toward vector control).

Thus, control of malaria in Africa is arguably where the use of self-sustaining gene drive mosquitoes could yield the greatest public health benefit, and, therefore, where their initial use would be most justified. Although there are still many issues to be resolved, initial indications are that low-threshold gene drive technology, if optimized, has the potential to be readily deployable across diverse geographical and socioeconomic areas, including low-income communities and those with poor access to health care, thus protecting millions of people and achieving extremely high impact over relatively short periods of time.^13^ Because of these potential benefits, NASEM and a WHO expert advisory committee have encouraged continued research on gene drive mosquitoes as a new tool to work synergistically with other malaria interventions.^18,32^

Although this working group considered only malaria transmission by *An. gambiae* s.l. in Africa, it is expected that the recommendations related here will have relevance to similar research on other mosquito vectors of malaria, including those prevalent in other regions, and on other disease vectors. However, the testing pathway will need to be reconsidered according to the specifics of these other cases.

## GENE DRIVE STRATEGIES

As defined in the WHO Guidance Framework, gene drive approaches that are “self-sustaining” (sometimes termed “self-propagating”) are intended to spread through the target mosquito population.^15^ The drive mechanism must be capable of overcoming any fitness costs and capable of increasing in frequency from low initial levels to fixation, or near fixation, in the population into which it was introduced within a time frame that will be meaningful for malaria elimination. Although other, more limited, approaches are now being considered (see *Self-limiting alternatives*), this definition remains valid for low-threshold gene drive strategies that are the subject of these recommendations.

**Figure 2:**
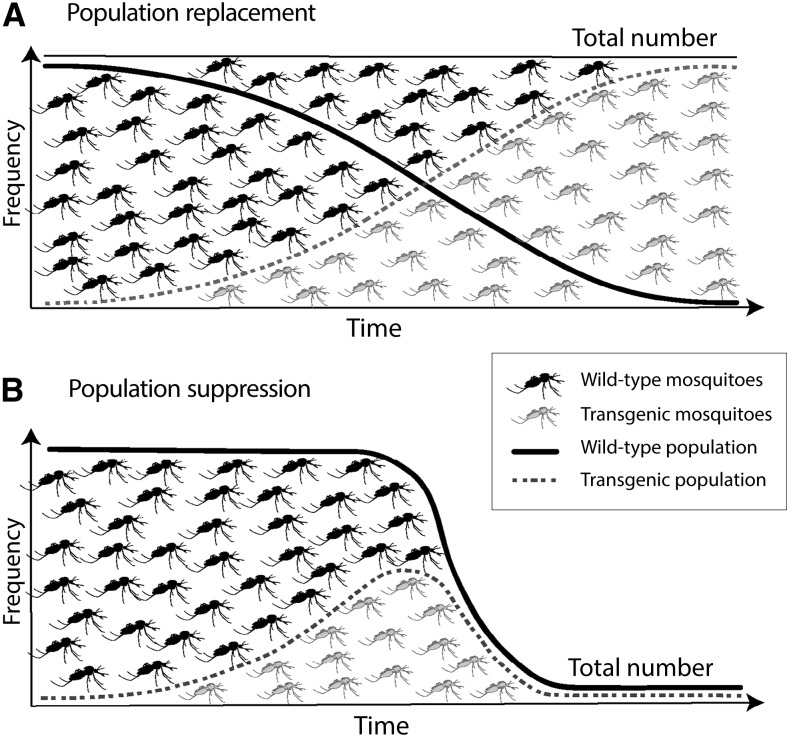
Comparison of population replacement (**A**) and population suppression (**B**) strategies.

There are two major categories of gene drive strategies—population suppression and population replacement§ (Figure 2). Computer simulation indicates that both have the potential to interrupt malaria transmission by the targeted mosquito species even in the most challenging settings, and these studies provide insight into how deployment methods and spatiotemporal extents can be tailored to local conditions to overcome obstacles such as extreme seasonality.^13^

Population suppression strategies are intended to reduce the size of the vector population to such an extent that it will not be able to sustain malaria transmission. This is an extension of the goal of all current vector-control products and does not require driving a population to extinction. Population suppression strategies are based on inactivation, or knock-out, of genes involved in the target mosquito’s survival or reproduction (e.g., reducing fertility or production of female progeny), and/or bias of the sex ratio toward males. These may be termed “loss of function” techniques.

Population replacement strategies are intended to reduce the inherent ability of individual mosquitoes to transmit the malaria pathogen. These strategies may be built around inactivation of a gene or genes that facilitate parasite survival in the mosquito vector or that are required for the mosquito to transmit malaria, such as a tendency to feed on humans. Population replacement strategies based on inactivation of genes directly involved in vectorial capacity are also termed “loss of function” techniques. Other population replacement strategies involve the introduction of a new gene or genes, such as those that produce effector molecules that will kill the malaria parasite in the mosquito. To perform successfully, such introduced genes must be carried into the mosquito genome in tight linkage with the gene drive mechanism.

Mosquitoes modified with low-threshold gene drive constructs are expected to persist in the environment. Those strategies aiming for population replacement require the modification to persist at high levels for as long as malaria continues to be transmitted to achieve their objective. For those strategies aiming for population suppression, modified mosquitoes are expected to decrease to low numbers over the period of a few years as the overall population of target mosquitoes is reduced. Phenotypic traits identified as relevant to efficacy and/or safety should be observed in the laboratory over multiple generations to obtain information on their stability. Because the anticipated mechanism of action and period of environmental exposure will differ among various gene drive strategies, researchers will be responsible for proposing an adequate plan for demonstrating the durability of efficacy in their regulatory applications. Modeling will provide a critical tool for determining the number of generations over which key stability, efficacy, and safety characteristics must be monitored in the laboratory to provide sound justification for proceeding to field-testing.

### Self-limiting alternatives.

There are circumstances in which consideration might be given to the testing of a self-limiting approach.^15^ Self-limiting constructs constitute a form of biological or molecular confinement, which would supplement physical and ecological confinement. The genetic sterile insect technique is the most extreme self-limiting technology, and several fertile but self-limiting or self-exhausting approaches also are possible. One such approach would use a closely related but nondriving version of the proposed self-sustaining construct, which is expected to be passed on in diminishing proportion through subsequent generations according to normal Mendelian inheritance until eventually becoming highly diluted in the population through outcrossing and lost if it conveys any fitness cost. Other proposed approaches include genetic manipulations aiming to purposely limit the time period or geographic range over which gene drive is expected to remain functional.^33–35^ Additional alternatives likely will continue to be conceived.^36^

The focus of these recommendations is on developing gene drive mosquitoes to contribute to elimination/eradication of malaria across sub-Saharan Africa, a vast continent^37^ where malaria is largely present in rural regions and endemic in most countries. To date, there are no modeling data to support the possibility that any of the currently contemplated self-limiting approaches might achieve an appreciable reduction of malaria transmission across sub-Saharan Africa.‖ However, the working group recognized three circumstances in which testing of a self-limiting intermediate before moving to the field with a self-sustaining driving construct could be particularly pertinent to the development pathway for gene drive mosquitoes to control malaria in Africa.

1. To provide additional data regarding the interaction of the genetic construct with the environment for a first-in-class gene drive strategy, if deemed necessary to answer questions raised in the risk assessment or to build confidence with regulators, communities, and other stakeholders. For example, this might be useful to gain multinational acceptance of a new technology. In this case, the composition of the self-limiting construct with respect to promoter, effector, and marker genes should be as similar as possible to that of the self-sustaining construct to maximize the relevance of information to be gained from this approach.
2. To conduct field testing for efficacy of population replacement strategies under conditions of lower risk. Initial efficacy testing may be performed using a laboratory strain of *P. falciparum*, and gametocyte-producing laboratory strains of the parasite are available. However, before progressing to field-testing of gene drive mosquitoes, it will be important to determine whether the construct exhibits predicted activity against locally transmitted parasite strains. Because malaria gametocytes only remain viable for a short period, such testing with local strains must be performed in a malaria endemic region. Vector competence for local parasites could initially be performed in-country using a self-limiting intermediate.
3. To provide additional training and capability strengthening for an unproven containment facility and/or inexperienced staff before initiating work with a low-threshold self-sustaining form of gene drive. Combined experience suggests that many breaches of containment are associated with human error due to failure to follow established procedures, emphasizing the importance of training and experience for physically confined studies. Initial work with a self-limiting strain will provide an opportunity for researchers to evaluate system capacity and compliance with standard operating procedures (SOPs) and understand regulatory requirements, under conditions of decreased risk.

Researchers should consider the following factors when making the decision on whether to include a self-limiting step in the development pathway for a self-sustaining investigational product. First, under most conditions found in sub-Saharan Africa, it is questionable whether a self-limiting version could be effective over a sufficient area and time frame to provide a cost-effective and sustainable reduction of malaria transmission. Thus, although short-term population reduction or replacement effects may be measurable, the feasibility of obtaining meaningful and cost-effective epidemiological efficacy, especially in those regions most in need of additional control tools to achieve malaria elimination, should be examined. Second, no matter how similar the self-limiting intermediate is to the intended self-sustaining version it cannot be expected to have exactly the same environmental interactions and implications because of the intentionally limited level and period of exposure. Finally, field-testing of a self-limiting intermediate will require a sizable expenditure of time and resources, which may result in an appreciable delay in the availability of a self-sustaining gene drive mosquito product for use against malaria. On the positive side, although a self-limiting strain may not in itself be an effective tool against malaria transmission in sub-Saharan Africa, it may enable researchers to gain useful experience and information that will increase the likelihood of success when the self-sustaining version is released. Testing of a self-limiting strain can also help to build relevant regulatory experience, allowing in-country regulators an opportunity to consider country-specific risk questions and to tailor or adapt their regulatory frameworks.

Careful consideration must be given on a case-by-case basis to determine what information can reasonably be acquired from testing a self-limiting intermediate, how vital this information is to decision-making, and how extensive such testing must be to obtain the necessary information. It is possible that regulators and policy makers may desire an intermediate step in the testing pathway. Researchers must be prepared to explain the advantages and disadvantages of such a step.

Self-limiting alternatives will be subject to relevant risk assessment and regulatory requirements for importation and use of GM organisms (GMO). Beyond that, because of the diversity of potential self-limiting approaches, it is possible that some confinement and release requirements described here for low-threshold gene drive may not be applicable. These should be determined on a case-by-case basis according to the nature of the construct.

### Follow-on products.

Given the current state of development, the recommendations provided here focus primarily on first-in-class applications of low-threshold gene drive technology. However, it is anticipated that this research will not end after field testing of the first gene drive mosquito product.

For purposes of these recommendations, the working group assumed that initial products will be targeted at *An. gambiae* s.l. It is expected that the transgenic construct can be transferred to other major vector species within this complex through interbreeding in the laboratory. There will be a need, however, to move the technology into other malaria vector species, notably to the *An. funestus* complex which is also important for malaria transmission in large regions of Africa^38^ and is developing resistance to common insecticides.^39^ This probably will require a new transgenic event and, thus, likely will be considered an independent investigational product.

Moreover, as has been observed repeatedly with insecticides and antimalarial drugs, it can be expected that resistance eventually will develop to first generation products through selection or evolution of variations in the targeted genetic sequence in the mosquito or parasite.^40–43^ Mechanisms are being explored to delay the expression of resistance (see *Resistance*). However, unless malaria has been eradicated before this occurs, resistance is likely to generate a need to identify effector mechanisms for population suppression or replacement that target different gene sequences.

Finally, gene editing is a rapidly evolving field of research, and new advances may result in second generation products with improved efficacy or other desirable features. Recognizing the likelihood of ongoing advances and new approaches in gene drive technology, this working group endeavored to avoid being too prescriptive in its recommendations, aiming to provide advice that will be relevant to current and future efforts to develop gene drive technology for use in mosquitoes. Nonetheless, it should be understood that some of the stated recommendations and requirements described here might be changed for follow-on products after uncertainties have decreased.

## GENERAL CONSIDERATIONS FOR DEVELOPING GENE DRIVE MOSQUITOES

Many of the issues that must be considered for field-testing an investigational product will be common to all low-threshold gene drive strategies and all along the continuum of the development pathway. Product development will be more efficient if planning for these issues begins early in the project (see Box 3).

Box 3 **Planning considerations for testing along the continuum of the development pathway**

Establish a team with appropriate expertise and experience

Develop/refine the target product profile (TPP) and criteria for advancement

Conduct modeling to inform experimental study design and understand potential benefits

Develop processes for information and data sharing to promote transparency

Establish partnerships as necessary

Characterize the field testing site (ecology, vector, and clinical)

Undertake environmental risk and biosafety assessments

Address ethical issues

Plan for and conduct stakeholder engagement at multiple levels

Plan for and meet regulatory requirements

Design remediation/mitigation plans

### Product characteristics.

The WHO Guidance Framework identified two major issues to be addressed in the critical path for development of GMM as public health tools: 1) proof of efficacy, determined through testing for entomological and epidemiological impact; and 2) evidence of acceptability, determined through biosafety, ethics and engagement activities, and compliance with regulatory requirements.^15^ Similarly, these two issues are priorities for gene drive mosquitoes. Development of a target product profile (TPP) will help researchers identify their specific goals in each of these areas and facilitate decision-making about when an investigational product is ready to move further along the testing pathway. The TPP is a tool that aids investigators to begin their work with the ultimate goal in mind, focusing on the specific claims of the envisioned product, and is frequently recommended for product development.^44–46^ Researchers must be able to articulate the rationale for the product, including the advantages it will provide beyond existing tools. Engagement activities should include consulting the potentially exposed community and relevant government authorities early in the process of TPP formulation to understand what characteristics would make the product attractive from their perspective. For example, this engagement could begin during the period in which baseline field data are collected. The TPP will include parameters such as efficacy, safety (including ecosystem impact), stability/durability, and production and release characteristics^18,47^ (discussed further in the following text). Establishment of TPP criteria should be informed by modeling. The TPP can be refined as additional field experience and data are obtained. However, even early in development, researchers should think about such practical issues as how the potential gene drive mosquito will be manufactured, distributed, and monitored, as this may influence fundamental decisions, including construct composition and whether to protect intellectual property (see **Implementation as a public health tool**). A cost analysis likely will be required before decision-making on implementation, so researchers should consider their goals for cost of eventual deployment.

### Development pathway.

The predicted properties of rapid spread and persistence that make low-threshold gene drive such an attractive tool for controlling malaria transmission also complicate the ability to remove the modification once it has become established in the local mosquito population should demonstrable harms be observed. Unlike GM crops for agriculture where the modification is not intended to spread, low-threshold gene drive modifications purposely are designed to continue spreading after releases of the gene drive mosquitoes are halted.

**Figure 3:**
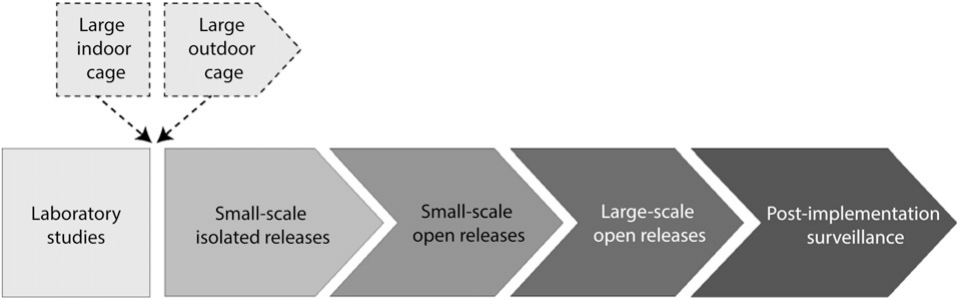
Pathway to deployment of gene drive mosquitoes.

Similarly to the pathway described in the WHO Guidance Framework,^15^ testing of gene drive mosquitoes is expected to proceed through multiple phases or stages, each incrementally increasing the degree of human and environmental exposure to the investigational product. The transition along the pathway will be subject to fulfillment of efficacy and safety criteria as defined in the TPP and evaluated in the context of specifically designed field trials, as well as regulatory and ethical approvals, and social acceptance. However, the characteristics of gene drive may make it difficult or even undesirable to delineate distinct cutoffs between phases in the testing pathway beyond initial studies under physically confined laboratory and insectary conditions. Thus, to avoid confusion, these working group recommendations consider the goals and requirements of successive major stages of field testing as a continuum of expanding releases, rather than as distinct or discontinuous phases (Figure 3).

As is the case with biocontrol agents, which also are expected to spread and persist in the environment and whose releases may be difficult to reverse, emphasis is placed on safety evaluation at the end of physically confined laboratory testing (including testing in large indoor cages that simulate the natural environment, if applicable) as a major decision point for whether to enter field testing. Initial transition to the field may begin with testing in a large outdoor cage (semi-field testing), although this was not considered an essential requirement. The first small field release should strive for geographic isolation to limit environmental exposure to the extent practicable as safety observation continues. Increasingly larger scale open releases will allow for assessment of first entomological and then epidemiological efficacy of the investigational gene drive product. Acceptance as a public health tool would initiate more systematic scale-up releases and initiation of post-implementation surveillance for ongoing efficacy and safety. Requirements for each of these phases are described in detail in the following text.

In determining the minimal requirements that an investigational mosquito product should meet to justify moving beyond the laboratory and along the development pathway, safety concerns and potential benefits to be gained from further testing both must be weighed. Benefit should be considered not only from the perspective of the final product, which aims to provide cost-effective control of malaria transmission, but also at each level of testing before expansion of releases is allowed. Researchers must be prepared to justify each study or trial to regulators and other decision-makers in terms of how the information to be gained will contribute to decision-making. Although safety will be assessed carefully before proceeding to field testing, ongoing consideration of safety is necessary as a matter of due diligence.

### Risk assessment.

The predicted ease of spread of gene drive mosquitoes calls for extremely thorough evaluation under careful confinement before release into a hospitable environment (i.e., conditions that could support the survival of the mosquito). However, as emphasized in the WHO Guidance Framework,^15^ it is important that safety expectations should be proportionate to those for other vector control tools and should take into account the risks associated with maintaining the status quo.

Risk assessment will provide guidance on decision-making for the project team, including information for preparation of regulatory applications and development of risk mitigation plans. This can identify additional questions that need further research to fully assess risk. The WHO Guidance Framework discussed risk assessment and risk management considerations at each phase of testing.^15^ Quantitative ecological risk assessment was endorsed in the NASEM report as especially useful for estimating the probability of specified outcomes.^18^ In their consideration of synthetic gene drives in Australia, the Australian Academy of Science recommended that any decision to release a synthetic gene drive be made on a case-by-case basis following a comprehensive environmental risk assessment which includes ecological and evolutionary modeling.^48^ Others have recommended an integrated approach to risk assessment of gene drive technologies that includes the participation of ethicists^49^ and biosafety professionals.

The risk assessment should be grounded in the protection goals established by the countries that would host the testing and/or use the technology.^50^ However, it should cover not only environmental and health risks, but also social and economic risks.^51^ There are challenges associated with weighing risks that the research team identifies as most significant against those of greatest concern to the lay public in risk assessments,^52^ which will be especially true for gene drive, a technology that is expected to cross national borders. Thus, there will need to be a plan for how public input on hazards is solicited and integrated. Principles for both environmental and social impact assessment have been proposed.^53,54^ The risk assessment conducted for testing *Wolbachia*-infected mosquitoes for controlling transmission of other vector-borne diseases provides an example,^55^ but this would have to be adapted to the context of gene drive applications in Africa.

An external risk assessment, conducted by qualified individuals with no vested interest in the success of the product, can be valuable for building community, stakeholder, and public confidence. This will be particularly important for first-in-class gene drive strategies. The working group recommended that researchers and/or funders commission an external all-hazards risk assessment, to be conducted by experts that are unaffiliated with the research project, and that results of the risk assessment be made publicly available. Funders should be prepared to support the costs for risk assessment as an integral part of the overall research plan.

The risk assessment must be reexamined and updated before moving forward along the testing pathway, to take into account any changes in human or environmental exposure, additional data, and any further public concerns.^56^ Although researchers are encouraged to make such external assessments available to regulators and the public, it should be understood that these would not supersede the risk assessments performed by the regulators in connection with evaluating applications, in compliance with national regulatory requirements and guidelines.

### Decision-making.

As described in the WHO Guidance Framework, it is expected that decision-makers will take into consideration criteria of both safety (risk) and efficacy (benefit) for a product’s intended use.^15^ Benefit will be perceived relative to the particular context, which in this case is the need for malaria control in Africa.

Early on in the development of individual gene drive mosquito products, funders will need to make important decisions about their commitment to move an investigational product to the field. This decision will be facilitated by mathematical modeling of the predicted effects under realistic transmission conditions. As the science continues to evolve, there always will be the possibility of new products on the horizon; however, anticipation of a better product needs to be balanced with the potential life-saving benefit(s) of a current investigational product if support is provided to complete the development and testing process. It can be expected that field-testing of gene drive mosquitoes will be rigorous and expensive. The effort necessary to move an investigational product forward through field testing in developing countries in a responsible manner represents a major commitment, as described in the following text. If an investigational product meets mutually agreed TPP criteria of safety and efficacy during contained laboratory testing, indicating that it could have a significant impact in reducing malaria transmission in the setting in which release is contemplated, a decision is made to move forward to field testing and releases begin; funders must be prepared to commit sufficient resources to meet long-term obligations to the researchers and to the countries where the testing will take place to which they committed in the research plan. It is advised that all involved institutions (including funders) develop a joint research collaboration agreement in advance, which makes each institution’s obligations clear.

### Modeling.

Just as pharmacokinetic/pharmacodynamic modeling is an essential component of developing and testing a new drug, mathematical modeling has an important role to play in each step of the testing pathway for gene drive mosquitoes. However, the validity of the modeling will be influenced by the strength of the dataset it utilizes, including its relevance to conditions at the trial site, and this underscores the need to collect relevant baseline field data and to make it widely available to the research community (see *Data systems*).

Even before developing constructs in the laboratory, mathematical modeling can guide the specification of required properties of the construct, such as homing/drive rate, effector strength, frequency of development of resistance, and more. Mathematical modeling can also help to identify baseline data required from potential field sites before the first field trials, which will assist in determining whether the sites under consideration will be sufficiently informative for the proposed trial objectives.

Computational modeling can use performance characteristics measured during early development (laboratory and large cage testing) to predict possible outcomes in open–field-testing for the investigational product before any actual field releases are performed. These types of model-based inferences provide an important contribution to decision-making about whether field releases are justified.

Data collected from small and large-scale field testing can be used in computational models to help plan a resource-optimized robust release strategy for wide-scale implementation that achieves the goals of disease control and elimination efforts before scale-up begins. Risks of resistance also can be explored in mathematical models to develop sampling schemes to identify any occurrences in the course of implementation with substantial potential effects on the disease. Modeling also may provide insights into the effectiveness of proposed remediation strategies.

### Transparency.

The working group members noted that development of gene drive technology carries an obligation for transparency and accountability. This is important for earning public confidence, ensuring that the product meets stakeholder needs, encouraging inter-project coordination necessary for responsible field testing, and minimizing any risks to human health and/or the environment. Gene drive researchers should commit to being appropriately transparent about their work.

With respect to public engagement, failure to be transparent about data can heighten anxiety by creating the impression that scientists know things they are not willing to reveal, and this may fuel distrust. From the perspective of product development, inappropriately conducted field trials have the potential to negatively impact the future success of other gene drive products; to undermine community, stakeholder, and/or public confidence in the technology; and to contaminate the regulatory and funding environment. Also, even though the release of an ineffective gene drive construct in the context of an efficacy trial may not appreciably alter mosquito function or result in any direct biosafety threat, such a release might create subtle genetic changes in the target mosquito population that could impact the effectiveness of subsequent investigational products and influence the use of subsequent new gene drive products at the study site. This could result in loss of time and resources spent developing other gene drive products and preparing field sites, and possibly prevent the sites from benefiting from future products. At worst, ill-conceived field trials might cause damage to human or animal health, or the environment. Thus, transparency should include, but is not necessarily limited to, keeping open and accessible records of any (accidental or intended) releases, containing a full description of the investigational product.

Policies and mechanisms for inter-project coordination and broader data and information sharing are a necessity. This level of cooperation is best driven by research funders, as exemplified by prior data sharing agreements.^57,58^ Recognizing the importance of transparency for public confidence and future development of gene drive technology, the working group recommended that funders work cooperatively on the early establishment of policies for appropriate sharing of data from gene drive research.

Researchers, funders, policy makers, and government authorities will need to consider whether currently available sites for publicly disclosing relevant information (e.g., the Biosafety Clearing House of the Convention on Biological Diversity (CBD), various clinical trial and nucleic acid databases, and national regulatory agency websites) are sufficient for gene drive technology or whether additional reporting mechanisms are necessary.

### Coordination.

The working group encouraged funders to support efforts to establish mechanisms for coordination across projects and programs on gene drive technology. Formation of networks among gene drive funders, researchers, and regulators and policy makers, could encourage information sharing and cooperation in areas of mutual interest and overall importance to the field. For example, coordination of communication strategies among teams working on similar technologies, different approaches, and/or in the same region is desirable and would contribute to research advancement through enabling better community, stakeholder, and public understanding. Such coordination should be encouraged by those who are aware of various projects within a region, such as academic institutions, regulators, ethics committees, and funders. A forum for researchers interested or involved in gene drive research would be especially useful to promote evidence-based self-regulation, sharing information on best practices, and supporting appropriate management of field trials.

Development of gene drive technology from initial research through field-testing and deployment will require complex interactions among researchers, funders, and national and international authorities at multiple levels, including broad alignment of public engagement efforts, biosafety, and ethical standards. The working group recommended the establishment of a neutral body empowered to manage high-level coordination among the various stakeholders and to organize centralized responses to the diverse challenges that will arise in the development pathway for gene drive mosquitoes as public health tools.

### Data systems.

Researchers are strongly encouraged to share field data openly and collaboratively for the greater benefit of the malaria research and control communities. Adequate database platforms for data gathering and storage for evaluation/analysis, therefore, should be available. It is recommended that the data be archived in centralized, widely accessible data repositories with the aim of having common data formats. VectorBase^59^ and PlasmoDB^60^ are examples of databases established for the purposes of such research. Mosquito data should not only include sequence information, but also extensive meta-data describing the type of mosquito (gene drive or wild), source of collection, and experimental study design. As data systems are being designed for field trials, it is recommended that they be developed following Clinical Data Interchange Standards Consortium (CDISC) guidelines. Clinical Data Interchange Standards Consortium is a nonprofit standards-developing organization and has developed some standards for data instruments for malaria research.^61^ Investigators could engage CDISC to develop the data ontology relevant to mosquito vectors and related information and expand this suite of standards for gene drive research.

### Ethical obligations.

The development and deployment of gene drive mosquitoes for control of vector-borne diseases will involve interaction with a diverse spectrum of groups, as recognized by both the WHO Guidance Framework and the NASEM report.^15,18^ The WHO Guidance Framework distinguishes between “communities” that live at the trial sites and “third parties” that also have interest in the research but do not live at the field trial site.^15^ The NASEM report defines “communities” as those who live in or near sites where gene drive organisms will be used and further distinguishes “stakeholders” as those who have direct professional or personal interest in gene drive and “publics” as those who lack a direct connection but have interests or concerns that may contribute to decision-making.^18^ The composition and extent of these groups likely will change with each successive phase of testing. It could be argued that because gene drive constructs theoretically could spread across large regions of Africa, most of the African population legitimately falls in the category of stakeholder regardless of where the trials begin. This points to the importance of engaging with regional and multinational bodies with authority to represent transnational sets of stakeholders.

As described in the WHO Guidance Framework, obligations to these different communities, stakeholders, and publics will vary in their ethical significance and may be addressed through a range of activities.^15,62^ Researchers are responsible for obtaining fair and legitimate authorization for field-testing gene drive mosquitoes.^56^ At the highest level, safety is a paramount public interest that is addressed through the regulatory mechanisms put in place by governments. Moreover, it is a standard requirement to obtain ethical clearance for any research involving human participation. The process for doing so may differ among countries, and in some may require considerable lead time.

At any point along the continuum of an investigational product’s testing, individual informed consent is required from those who meet the internationally accepted criteria of research subjects (examples of requirements may be found at the websites of the WHO^63^ and U.S. Department of Health & Human Services, Office for Human Research Protections^64^), such as those who provide clinical specimens or identifiable information at the individual or household level.^65^ However, simply living near a vector release site does not qualify someone as a research subject.^66^ Nonetheless, researchers are obligated to respect the interests of those within the community(ies) hosting trials of gene drive mosquitoes who, although not research subjects, may be associated with and/or affected by the research in a meaningful way. As discussed in the following text under community engagement, this requires practices undertaken to inform such persons about the project, and to understand, respond to, and learn from their perceptions and reactions in a way that makes it clear their opinions have influence.^15,56,62^

Once the decision to field-test a particular investigational product has been made, researchers and funders incur a responsibility for the safety of the host community. Prematurely discontinuing field-testing and/or monitoring for lack of funding could be considered irresponsible. Funders must be prepared to commit to continued support for trial and post-trial activities as long as is required by regulators and by ethical obligations to the community hosting the field testing. Likewise, researchers should not initiate field releases until adequate funds are secured to carry out their regulatory and ethical obligations.

Given the complex ethical and community engagement issues accompanying gene drive technology, an ethics advisory group comprising experts external to the project would be an important mechanism to supplement the input from community advisory boards or other community engagement activities, providing additional and broader perspectives. This group would be distinct from the institutional or national ethics committee to which researchers must submit their proposed activities for review and approval, and would advise the researchers on ethical issues related to the project. This advice could be especially helpful in determining how to anticipate and address controversial or sensitive issues. Mechanisms should be established to allow this group to obtain relevant information on issues such as risk assessment, policy, engagement activities, and trial status from the project and other advisors. The working group strongly recommended that researchers establish an independent group of ethics experts that is external to the project team and includes in-country experts and those from involved communities, to advise their projects throughout the research and field testing trajectory.

### Engagement.

Appropriate engagement will be crucial to the success of the research on a number of levels. Therefore, funders must be prepared to provide support for ongoing engagement activities as an integral component of the research plan. Acceptability of the research project, and of the ultimate gene drive mosquito product, is fundamental to its success. Engagement is essential to meeting ethical obligations of informed consent, building trust, and gaining acceptance of the research. When conducted through an open exchange of ideas, engagement can also support knowledge sharing that leads to development of a better and more acceptable product. Engagement will be an iterative process that continues throughout the development pathway, understanding that opinions can change over time. Consideration must be given, however, to mechanisms to monitor for and avoid stakeholder fatigue over the course of lengthy trials.

Before releases begin, researchers, in collaboration with government authorities of countries hosting the trial, funders, or other advisors should create a plan for achieving effective engagement with communities and other stakeholders, thereby providing for opinions of various groups to be considered in the decision-making process over the course of a project. For this, it will be important to conduct a systematic analysis of influential stakeholders at different levels.^67^ At the early stages of research, in addition to in-country members of the project team and community members, researchers should seek to learn from other in-country and/or regional experts and organizations familiar with the local political, religious, social, and cultural structure to establish an appropriate engagement strategy. It is important to understand the different levels of government when planning the engagement approach and respect the requirements at each level. Researchers should engage early with relevant ethics committees (e.g., institutional or national) for field sites to determine the extent of public engagement required in preparing for and conducting field studies, and guidance in identifying local leaders and key influencers (religious, community, civil society, and media) who should be consulted. Researchers should coordinate engagement efforts with existing regulatory processes and relevant agencies that will be involved in deploying the product. Involvement and input by the end user of the technology, which in the case of gene drive mosquitoes is likely to be the national malaria control program and/or Ministry of Health or equivalent, can substantially facilitate public engagement.

The precise nature of community engagement will vary from context to context but must be assumed to require long-term commitment and substantive funding. A common principle is that communities should be provided with sufficient opportunity to interact with the project team to learn about the research and its implications to formulate reasoned positions about whether to host a trial. Information about the research and investigational product must be provided and discussed with communities and other stakeholders in a way that strives for all voices to be heard. The expectation is for community engagement with open and honest exchange of ideas and information. Researchers should seek to learn from these groups about ways they can improve the project or the product. Researchers and funders must be open to the possibility that research plans may need to change in response to community input or even that an ongoing project must be halted or moved.

The mechanism by which communities will indicate their authorization or endorsement for a trial to proceed likely will differ according to cultural contexts and may evolve over sequential phases of testing. Researchers should discuss with the community how it wishes to be consulted and what it considers to constitute authorization to proceed with testing. Involvement in trial planning is important to foster community ownership and identification with the research. The ideal outcome is community-driven support for the gene drive intervention. Researchers should commit to updating the community with current information periodically, and particularly if plans change. Transparency will be central to trust building.

At all levels of engagement, co-ownership of the entire product development and testing process by in-country scientists and government authorities will be critical for acceptability. Because the nature of effective engagement is so context specific, it is best undertaken by people who are locally known and respected and have deep knowledge and understanding of the local value system and culture. Social scientists, ethicists, and other experts experienced in engagement should be included in the research team to develop and implement the stakeholder engagement plan. All members of the project team, however, will interact with the community on some level as part of their ongoing activities and, therefore, it is crucial to ensure that all team members are informed and able to provide accurate information about the project and its goals.

Community engagement should not be conflated with or mistaken for public relations or marketing and does not imply advocacy. Examples of successful community engagement for other new technologies are available for guidance,^68–70^ as is more general guidance.^18,69–77^

Because of the potential for geographic spread of gene drive mosquitoes, engagement must expand rapidly to the national and multinational levels (as addressed for each testing phase in the following text). Government-level championship of the research will be critical by the stage of large-scale testing. Considerations for broader public engagement were described in the WHO Guidance Framework, which advocated for an “honest broker” approach that recognizes and responds to the value-based perspectives of third parties.^15,78^ The NASEM has published an evidence-based framework to guide science communication.^18,79^

### Communication and outreach.

Good communications materials, translated into the appropriate language(s), will be vital for explaining the technology and, therefore, will underpin engagement efforts at all levels. Researchers should include experienced science communicators on their team, as well as sociologists and linguists to help develop the necessary vocabulary to accurately and understandably convey the technical aspects of the research to each group of stakeholders. Project communications should be developed in coordination with appropriate authorities and emphasize information of interest to the community, which might include utilitarian benefit, sustainability, and prudence. Communication materials should include: clear, current, and understandable information describing the project; a set of frequently asked questions that anticipate confusing or controversial issues; and a crisis communications plan for handling emergencies, including methods for rapid dissemination of information and surveying public perspective.

Communication strategies may differ for different audiences, but details regarding project goals, timelines, planning, and execution must remain consistent for a specific project. Local media outlets, such as radio stations, can be useful for making the community aware of the research and where to obtain more information. Fostering well-informed media is an important consideration throughout all facets of product development and testing. It will be important to engage proactively with the media, for example, by offering accurate, fair, and balanced informational sessions and tours of the research facilities. This will help the media to obtain a basic understanding of the project and to provide accurate material and information about gene drive technology. Researchers should identify project spokespeople and provide them with communications skills that will enable them to explain the project clearly to stakeholders and the media.

From the beginning, researchers should have a plan for interacting with those who do not agree with the conduct of research on gene drive mosquitoes in their community. Some who disagree may hold deep seated objections that limit compromise, whereas others may seek changes or have concerns that could be addressed and would, therefore, be amenable to dialogue if engaged.

Researchers may be confronted with well-organized dissent, which could originate within or outside the community where the research is being conducted. Execution of a robust, proactive engagement plan may help mitigate against negative messaging. Relevant study personnel should receive support and/or formal training in conflict management and in communications. However, the most important factor will be the relationships that have already been built with key stakeholders, including the community, in-country scientists, media, civil society, policymakers, regulators, and relevant government authorities. In-country champions and supportive voices are best positioned to respond to dissenting opinions from the outside.

### Policy and regulatory considerations.

Regulatory oversight can be expected at many levels–federal, local (e.g., state, province, county, district, or region), and institutional.^15,80^

Gene drive mosquitoes are intended as public health interventions; therefore, malaria endemic countries where these products will be deployed are likely to seek advice from the WHO. The WHO mandate is to provide leadership in areas of global public health. As a result, member countries task the WHO with developing policies and strategies to prevent vector-borne diseases and to respond to outbreaks. The WHO plays an important role in supporting countries that lack the technical capacity in regulation, assessment, and operational use of new technologies. The WHO will consider safety and public health efficacy to be of high importance in its policy making for this technology. However, even in early stage field releases, it is anticipated that authorities (including international bodies such as the WHO and in-country regulators) will want some assurance of potential for benefit. The WHO Vector Control Advisory Group (VCAG)^81^ reviews evidence on new potential vector control approaches for malaria and neglected tropical diseases and makes recommendations to the WHO on their public health efficacy for purposes of policy development. Thus, VCAG offers a useful starting point for WHO interactions. Researchers should engage with the VCAG early in the project development process to obtain advice on trial planning and to help inform policy development for gene drive technology as a vector control tool.

Most malaria endemic countries where gene drive mosquitoes might be tested or deployed are signatories to the Cartagena Protocol on Biosafety (see Box 4), which addresses transboundary movement of GMO.^82^ In these countries, GMO usually are regulated by a National Biosafety Authority (NBA) or Committee, which derives its authority through a national biosafety law or other existing laws. Under Article 17, the Cartagena Protocol requires countries to notify other countries that might be affected by an unintentional transboundary movement that may have an adverse effect on biodiversity. Country obligations under the Cartagena Protocol may be affected by ongoing discussions within the CBD.^83^

Box 4 **The CBD and related multinational agreements**

The CBD is a multilateral treaty under the auspices of the United Nations Environment Program. Its major goals are the conservation of biodiversity, sustainable use of the components of biodiversity, and fair and equitable sharing of benefits arising from genetic resources stemming from biodiversity. The Cartagena Protocol on Biosafety to the Convention aims to ensure safe handing, transport, and use of living modified organisms resulting from modern biotechnology that may have adverse effects on biodiversity, also taking into account risks to human health. The Nagoya Protocol on Access and Benefit Sharing is a supplementary agreement dealing with fair and equitable sharing of benefits arising out of the utilization of genetic resources. The Nagoya Kuala Lumpur Supplementary Protocol on Liability and Redress is a supplementary agreement to the Cartagena Protocol that aims to provide international rules and procedures related to response measures required in the event of damage resulting from living modified organisms.

Because gene drive mosquitoes are intended as a public health tool, it is desirable to have the Ministry of Health or equivalent, which is likely to have malaria control as part of its mandate, engaged in the regulatory process. Health regulators, who will consider the potential public health impact of gene drive mosquitoes, should be included in discussions as early as possible. Through the support of the New Partnership for Africa’s Development (NEPAD) (an agency of the African Union), the WHO, and other partners, African countries are building their regulatory systems for health technologies, which are being led by National Medicines Regulatory Agencies. African countries also are revising their laws for regulating health technologies based on the Model Law for Regulation of Health Technologies^84^ that was adopted by the African Union in 2014. Key in these laws are the aspects of mutual recognition and regional collaboration which the countries may use in strengthening their regulatory systems. Although the Ministry responsible for health, along with other relevant ministries, is usually represented on the NBA, it will have to play a more proactive role in regulating gene drives for malaria control and elimination.

Whether a gene drive candidate is developed outside a malaria-endemic country and must be transported to an in-country institution for testing,¶ or a gene drive candidate is developed by an in-country team, approval must be obtained from the country-specific NBA for contained use. In the first case, approval for contained use enables a permit for import to be issued by the relevant quarantine authority. Researchers should interact with the country’s NBA as early as possible to provide them with information about research plans and goals and to determine the requirements to be met. Those who will be exporting an investigational gene drive product must consult with the biosafety office at their home institution and at the receiving institution before transfer. Requirements for export and import permits are likely to apply. Appropriate safety measures must be taken for transportation.^85,86^

Although several sub-Saharan African country NBAs have experience regulating GM crops,^87^ few have experience with GM insects and none presently have experience with gene drive mosquitoes. Gene drive mosquitoes will introduce new concepts for regulators. For example, with GM crops, experienced regulators usually consider the possibility for an introduced transgene to go to fixation or achieve high frequency in populations of sexually compatible wild relatives as part of their risk assessment. However, gene drive differs in that it is the intentional goal to achieve fixation or high frequency rapidly in the wild population of target mosquitoes, and this can be expected to create special risk assessment considerations. Regulators must understand that for release of gene drive mosquitoes, the modification is anticipated to persist in the wild population for an indefinite period, the expected length of which may differ according to whether a population suppression or replacement strategy is being tested. It is possible that this may require legal clarification, but precedents can be drawn from regulation of classical biocontrol agents.

Governance of gene drive technology and regulatory capacity building has been identified as a priority.^18^ New Partnership for Africa’s Development provides support and assistance to national regulators in conducting their risk assessments.^88^ The working group strongly encouraged funders to work with intergovernmental organizations such as NEPAD to support regulatory training and capability strengthening. Any such training must be neutral and balanced; information sharing must cover both potential benefits and risks of the technology objectively and in a transparent manner.

### Regional approaches.

Given the intention for gene drive mosquitoes to spread beneficial modifications throughout contiguous interbreeding mosquito populations and species, it is expected that the modification eventually will spread across national borders. The working group emphasized the desirability of regional approaches to testing of gene drive mosquitoes that would facilitate a multi-country regulatory review and authorization process, and encouraged relevant stakeholders, including government authorities, to support a regional strategy. For *An. gambiae* s.l., sub-Saharan Africa is the relevant region. Funders and researchers should work with organizations such as the NEPAD to facilitate and support regulatory harmonization efforts within the African region. The African Union defines eight regional integration bodies that are considered as operational arms on regional matters.^89^ These Regional Economic Communities play an important role in coordinating member states’ interests in many areas, including health and development, and will be important components of any regional strategy to prepare for transboundary movement of gene drive mosquitoes. The WHO also has a role to play in convening regional stakeholders.

### Remediation.

Researchers, funders, and government authorities should work together to reach an understanding on liability issues and trial insurance requirements before beginning field testing. Trial liability insurance is an important risk management consideration. All should be aware of the status of the Nagoya–Kuala Lumpur Supplementary Protocol on Liability and Redress to the Cartagena Protocol on Biosafety^90^ and the requirements of local laws.

Remediation of an investigational gene drive mosquito product once it becomes widespread is likely to be complex and challenging. Thus, the emphasis must be on risk assessment before each stage of testing with a goal to identify possible adverse consequences and inform measures to prevent them. Nonetheless, researchers should anticipate that regulators may request a remediation plan in the context of their applications for field-testing gene drive mosquitoes.

Remediation options are likely to be case specific, and dependent on the gene drive strategy, the stage of testing, the location and the issue being remedied. Risk management planning should include consideration of various mitigation and remediation options for nonnegligible effects on a case-by-case basis. Researchers should consider remediation options in the context of each stage of testing and ensure that these are appropriately evaluated, planned, and funded. Partnering with national vector and malaria control programs may be useful in the design of remediation plans; for example, in some cases large-scale campaigns for indoor residual spraying with insecticides might achieve both a vector control goal and a remediation goal. Modeling should be conducted to predict the effectiveness of the remediation strategy.

The working group members suggested that intense application of standard pesticides followed by monitoring would be a logical remediation strategy for semi-field testing and small-scale releases. This will be a familiar vector control strategy to regulators, public health authorities, and communities. In this case, researchers must make plans to have the remediation materials (pesticides or other methods) and necessary equipment on site, along with staff trained in their proper administration for maximum effectiveness. This will require that researchers ascertain in advance the pesticide susceptibility of both the gene drive mosquitoes to be released and of the local wild-type mosquitoes to the pesticide(s) chosen for remediation efforts and the feasibility of using that pesticide in the wider context of national malaria control or elimination efforts.

In the unlikely case that remediation becomes necessary for a larger scale release, such remediation would require additional vector control methods to supplement standard procedures such as indoor residual spraying and larval source management. The choice of remediation, or mitigation, method likely will be dependent on the nature of the exposure and the predicted harm. If the overall effect of gene drive mosquitoes is found to be beneficial, it may be possible that a particular harm can be mitigated without need for their wholesale removal. It is worth noting that gene drive technology offers additional hypothetical remediation possibilities not necessarily available for other biocontrol agents. These include release of sufficiently fit naturally occurring or GM variants of *An. gambiae* carrying a nuclease-resistant allele that restores function of the mosquito gene that was the target of the gene drive construct, or the release of another driving construct designed to inactivate the original driving construct (sometimes known as a “recall” construct).^8,91–93^ Researchers must remain aware of new technological developments that could contribute to remediation options. The working group encouraged additional research and modeling to investigate the utility of various remediation options for specific gene drive strategies.

Any remediation or mitigation method based on genetic modification also will be subject to risk assessment and regulatory approval. Researchers must consider the regulatory and ethical obligations for deploying a novel remediation method, including the appropriate timing for obtaining community authorization. The working group recommended that researchers explore the receptivity to genetic remediation methods with regulators early in the planning process. If such a method is to be used effectively to counteract an unforeseen event, it likely would need to be approved for release at the same time as the original investigational gene drive product to ensure that it is immediately available and ready for use before deployment at the time it might be required. The extent to which efficacy of such a method must be demonstrated before approval should be agreed in advance with regulators. However, the working group suggested that any novel remediation method should be tested for effectiveness to the furthest extent practicable before the testing stage in which it would be proposed for use, bearing in mind whether a premature release of the remediation construct at a trial site could preempt further testing of the investigational product there.

### Key points: general considerations for developing gene drive mosquitoes.

- Researchers should develop a TPP as early as possible to help identify goals for efficacy and safety, and initiate consideration of issues relevant to manufacturing, delivery, and cost. This will facilitate decision-making about when an investigational product is ready to move further along the testing pathway. Establishment of TPP criteria should be informed by mathematical modeling. Criteria can be refined as more data is gained from testing.
- Collection and evaluation of baseline vector, epidemiological, and ecological data should begin early to allow for observation of multi-seasonal and multiyear variation. Efforts should be made to anticipate conditions under which efficacy trials will be conducted, especially with respect to other vector control methods in use, as this will impact the relevance of the baseline data.
- Government oversight of gene drive mosquitoes will combine aspects of both biosafety and health regulation, and also introduce issues that are different from prior experience. Researchers must interact fully and transparently with regulatory and other government authorities as early as possible to provide information about research planning and goals, and to determine what requirements likely must be met. Funders should work with independent organizations such as the NEPAD to support neutral and objective regulatory training and capability strengthening. Regional approaches to testing of gene drive mosquitoes that would facilitate a multi-country regulatory review and authorization process are critical and must be encouraged and supported.
- Appropriate ethical clearance is necessary for studies involving human participation. At any stage of testing, informed consent is required for those who meet the internationally accepted criteria of research subjects. Those living at or near the field-testing site who are not participating directly as research subjects should be sensitized and mobilized via community-based engagement activities. Researchers, in collaboration with government authorities, funders, or other advisors, must put a plan in place for how engagement at various levels will be managed, which will be facilitated by including community engagement and communications experts on the research team. Funders must provide support for vigorous engagement activities as an integral component of gene drive research. Once the decision to field-test a candidate has been made, researchers and funders incur a responsibility for the safety of the participants and must commit to any follow-up activities required by regulators or imposed by ethical obligations to the community. Field testing must not begin until adequate funding is available to fulfill these responsibilities. Researchers are advised to consider appointing an external ethics committee to advise on sensitive or contentious issues.
- Testing must be conducted incrementally, increasing the level of human and environmental exposure only after the investigational product has fulfilled agreed on safety, efficacy, and acceptability criteria in the prior phase. Before moving from one level of testing to the next, researchers or funders should commission an external all-hazards risk assessment; this will inform project planning and decision-making, and also build trust if made publicly available. Risk assessment should cover not only environmental and health risks but also social and economic risks, and consider concerns expressed by affected communities. During early engagement, researchers should consider asking the community how they wish to be consulted and what they consider to constitute authorization to proceed with testing.
- Consideration should be given to the utility of developing a self-limiting intermediate. Initial field testing of a self-limiting intermediate could be especially useful to gain: information for risk assessment for first-in-class strategies where there is no relevant prior experience regarding potential environmental interactions; experience with working under containment conditions in areas where new facilities are created; and, for population replacement strategies, locally relevant information on efficacy under conditions of decreased risk. However, such intermediates will still be subject to regulatory requirements for GMO, and extensive testing will expend time and resources. Therefore, careful thought must be given to how vital the information from a self-limiting intermediate will be for decision-making, and how extensive the testing must be to obtain the necessary information.
- Researchers should commit to being appropriately transparent about their work. Baseline field data should be shared for the benefit of the other malaria researchers and control programs. Open and accessible records should be kept of any (accidental or intended) releases. Researchers, funders and government authorities will need to consider whether currently available sites for publicly disclosing trial information (e.g., the Biosafety Clearing House of the CBD, and various clinical trial and nucleic acid databases) are sufficient for gene drive technology or new mechanisms should be put in place.
- Researchers are advised to consider remediation options in the context of each level of testing and ensure that these are appropriately planned and funded. These may involve application of currently available pesticide-based methods and/or other methods developed in the context of gene drive research. Any remediation technology proposed must be ready for use at the time it is required, which for novel methods (such as genetic remediation) likely will mean generation of sufficient data and information to satisfy safety and efficacy needs of regulators and other stakeholders. Proposed remediation methods must be considered in the context of existing vector control and malaria elimination efforts.
- Researchers should be cognizant of the role of the WHO in reviewing and recommending new vector control products for the developing world. Early and ongoing interaction through the WHO VCAG is strongly recommended.

## PHYSICALLY CONFINED LABORATORY STUDIES

As described in the WHO Guidance Framework, testing of new investigational gene drive products begins with small-scale laboratory studies for efficacy and safety testing under appropriate containment conditions and operating procedures.^15^ This phase may proceed through testing in larger population cages within the laboratory setting, including large environmentally controlled indoor spaces that aim to simulate a field setting.#

It is recognized that gene drive constructs and modified mosquito strains may be created in laboratories outside malaria endemic regions, in which case they must be transferred to laboratories in locations suitable for *An. gambiae* establishment for further testing, or they may be created at laboratories in locations suitable for establishment. These recommendations for contained testing requirements relate to both possibilities.

Issues such as physical confinement requirements and engagement, monitoring, or remediation obligations, are expected to differ when initial research is conducted in a location that is not conducive for establishment of the gene drive mosquitoes under development versus a location hospitable to mosquito survival. It has been argued that all laboratory gene drive experiments should use at least two stringent confinement strategies, one of which could be performing the studies outside the habitable range of the organism^85^ (ecological confinement). Because this confinement option would not be available when early discovery research is performed in laboratories in malaria-endemic countries, other forms of confinement would need to reduce the probability of release to a level that is acceptably low.

### Safety.

The working group asserted that safety is the paramount consideration for gene drive mosquitoes and recommended several actions to improve the safety of the approach.

Initial research in an environment receptive to the establishment of gene drive mosquitoes should be performed in the context of the genetic background of the mosquitoes of the target species inhabiting that environment (e.g., laboratory-maintained strains derived from local wild-type mosquitoes). Not only will this minimize the chance of introducing incidental traits from nonlocal laboratory lines into the local mosquito population, but it also will maximize the ability to detect changes conferred specifically by addition of the transgene construct. It will be important to understand key characteristics of the local mosquito strain (e.g., insecticide resistance, fecundity) as this will be the most ideal comparator for risk assessment purposes.

If an appropriate facility and well-trained staff needed for maintaining containment are not available at the African institution, then initial safety work with driving constructs should be performed in a region unsuitable for establishment but in mosquitoes with the relevant African genetic background. If mosquito strains developed in Africa are exported for this purpose, researchers must inform themselves about, and operate in compliance with, local laws from the site where the mosquitoes were derived regarding protection of genetic resources (see Nagoya Protocol on Access and Benefit-Sharing^94^). Any out of country safety work should include the participation of scientists from the African partner institution(s) to ensure co-development and continuity. Relevant in-country regulators should be able to visit, inspect, audit, and learn from such off-shore studies if they wish, to ensure confidence in the data. The off-shore work also must comply with the various regulatory requirements of the country where it is being conducted. As new facilities are developed in areas compatible with establishment of gene drive mosquitoes, a period of laboratory and insectary work with naturally occurring mosquito genetic variants and/or nondriving strains of GMM should be undertaken to provide an opportunity for the site to develop capability and gain appropriate operational and regulatory experience (see *Containment requirements*).

Because of the persistence and spreading characteristics of gene drive technology, safety concerns must be explored and addressed to the greatest extent possible in the context of contained (small and large-scale laboratory) studies. As is the case for introduction of biocontrol agents, researchers should have a high degree of confidence in the safety of their technology before applying to move beyond contained studies. However, recognizing that some questions about safety may not be answerable by laboratory studies and modeling, such as some ecological interactions, these recommendations provide for ongoing safety observation in the field as a matter of due diligence.

#### Biocontrol analogy.

All aspects of safety identified by risk assessment should be carefully investigated under appropriate physical confinement, as described in the following text. In addition to assessment of the current investigational product, the possibility of future phenotypic variants arising because of the presence of the transgenic construct that might result in an increased probability for harm should be addressed. The risks posed by a proposed release need to be assessed and judged as acceptably low by the regulatory authority based on laboratory data before any release from the containment facility (see Box 5).

Box 5 **Go/no-go decision-making for moving to field testing**

The safety standard for moving an investigational gene drive product from physical confinement to field testing should be a well-reasoned justification that it will do no more harm to human health than wild-type mosquitoes of the same genetic background and no more harm to the ecosystem than other conventional vector control interventions.

The need to base a safety determination on data obtained from studies conducted in physical confinement is analogous to the process for evaluating exotic organisms as biocontrol agents, which also are intended to persist and spread indefinitely in the environment following release. The evaluation at this stage should provide a comprehensive evidence-based prediction of the safety of future unrestricted release of the gene drive mosquito strain. However, as is consistent with releases of other biological control agents, continued monitoring for negative environmental and health impacts throughout the release and post-release assessment processes is recommended by the working group.

Potential harms of *An. gambiae* mosquitoes with driving transgenes have been considered in a problem formulation exercise, which concluded that the pertinent protection goals will be human health, biodiversity, animal health, and, to a lesser extent, water quality.^50^

Previous reviews have dealt in detail with risk assessment considerations related to human and animal health and biodiversity.^15,95^ Table 1 summarizes several of the hazards related to human and animal health that researchers and regulators should consider on a case-by-case basis in performing risk assessments on each mosquito strain containing gene drive constructs.

**Table 1:** Prominent safety considerations related to human and animal health

Potential harm	Example hazards	Assessment Parameters*
Increased disease transmission	Increased abundance of vector mosquitoes	Fitness components including^15^:
		Growth rate
		Mating success
		Fecundity
		Adult, egg, or larval survival
		Environmental tolerances

	Increased vectorial capacity	Host seeking and biting activity^15^
		Vector competence* (*Plasmodium* or other pathogens carried by *Anopheles gambiae*)
		Change in temperature tolerance that could affect environmental niche or range

	Reduced control capability	Insecticide resistance*

Increased direct pathology	Increased allergenicity	Known allergenic sequences expressed by construct; construct-encoded proteins detected in saliva

	Increased toxicity	Standard toxicity test on construct-encoded proteins

	Increased parasite virulence (population replacement)^96^	Genotypic or phenotypic changes in parasites after passage through gene drive mosquitoes†

* Changes to be assessed in comparison to local wild-type mosquitoes of the same genetic background.

† This would best be performed with gametocytes collected from the field testing site to reflect the diversity of parasite strains circulating at the location and will not predict the evolutionary consequences of ongoing interactions of the parasite with the mosquito and vertebrate host over time.

The most frequently voiced concerns regarding biodiversity relate to loss of mosquitoes as a food source (most relevant to population suppression strategies) and potential that horizontal transfer of genetic material might cause harm to other species. Both have been considered unlikely pathways to harm^51,97^ but should be considered in the case-by-case risk assessment of specific gene drive constructs.

With respect to methods for consideration of nontarget effects, it may be useful to take lessons from conventional biocontrol (Table 2).

**Table 2:** Some considerations for possible effects of *Anopheles gambiae* containing gene drive constructs, extrapolated from consideration of biocontrol agents on nontarget species

Potential effect	Concern	Relevance for *An. gambiae*	Trigger for concern
First-order genetic	Construct might spread to a second species through interspecific mating.	May be anticipated and a deliberate part of the implementation strategy—for example, a gene introduced into *An. gambiae* s.s. is expected to spread into closely related vector species such as *Anopheles coluzzii* or *Anopheles arabiensis*. This would be useful for preventing malaria transmission by a second malaria vector, but the possibility of more distant gene transfer through interspecific mating also must be considered in risk assessment	Genetic evidence for low-frequency intraspecific mating outside the *An. gambiae* complex

Second-order genetic	Construct might spread through some other, non-mating, process to a second species	For example, the construct might move into a mobile genetic element that could be transferred through a microbial vector	Genomic evidence for the transfer of genetic material between mosquitoes and distantly related species

First order ecological	Removal of a species from a community might harm species that directly feed on it or which rely on the species for pollination.	The extent to which a predator or plant relies on *An. gambiae*. The harm done to *Plasmodium* through the removal of its vector is an example of a deliberate, anticipated, and beneficial first order ecological effect, but the possibility of detrimental effect on other, more valued, species also should be considered	Evidence that *An. gambiae* s.l. makes up a considerable fraction of the diets of specific predators in the same ecosystem, or that particular plants are largely pollinated by these species

Second order ecological	An indirect ecological effect resulting from removal of a species allows an increase in the density of another species (or resource) on which it fed (first order effect), which in turn allows a competitor species to increase in density by utilizing the unused resource	Removal of *An. gambiae* might result in increased abundance of another species, with detrimental effects	Presence in the same larval habitats as *An. gambiae* of other species of mosquito that share the same food source and pose a worse threat to human health; evidence of indirect ecological effects, including adaptation of the malaria parasite that have arisen after other successful interventions that have reduced *An. gambiae* density (such as bed nets)

Higher order ecological	An ecological perturbation causes further effects that ripple through the ecological community, and which are amplified rather than being damped	Addition or removal of a keystone species have major effects in ecological communities	A plausible mechanism based on comparative ecological studies showing how *An. gambiae* could act as a keystone species

Barratt et al.,^98^ summarize the general considerations in conventional biocontrol for selecting host species for host-specificity testing, and these also may be considered as instructive for gene drive mosquitoes. They describe a hierarchical method for identifying nontarget species that meet the most “at risk” criteria, based on potential for interaction with the biocontrol agent, and conclude that the number of species to be tested must be decided on a case-by-case basis depending on the length of the “at risk” list, the number of positive results from initial testing, and regulatory requirements.

For control of disease transmission by mosquitoes, the population replacement strategy employing *Wolbachia* provides a useful biocontrol precedent. The Eliminate Dengue project has published several studies on risk assessment and biosafety of their technology.^51,55,99–103^

### Efficacy.

Typical efficacy measurements at all phases of testing have been described in detail in the WHO Guidance Framework,^15^ and they remain relevant for gene drive mosquitoes. In physical containment, these include mating competitiveness, fertility and fecundity, rate of spread of the transgenic construct, and population suppression or replacement functionality measures (e.g., rate of suppression in laboratory cage studies or capability to host and transmit parasite isolates, respectively). Planning ahead to field trials, fitness characteristics are critical because the ability of gene drive mosquitoes to survive over the dry season will be fundamental to their success. Understanding that there may be a need for testing of self-limiting intermediates to gain information and experience before identifying a useful gene drive product (see *Self-limiting alternatives*), and that specific efficacy criteria will vary according to the different types of investigational products, the working group felt it was inappropriate to define minimum acceptable efficacy criteria for moving to field testing with one exception (see *Resistance*).

While still in laboratory testing, researchers should begin to develop a TPP that describes the attributes of the desired product.^18^ This will be informed by modeling of the characteristics that will be necessary to provide the desired effect on malaria transmission.^13^

#### Resistance.

The working group agreed on one characteristic that would constitute an effective “no-go” criterion to stop further testing for an investigational self-sustaining gene drive product intended for future use in malaria control, namely the likelihood of resistance arising too rapidly for the product to have a beneficial effect against malaria transmission. Every effort must be made during initial laboratory studies to mitigate against this possibility. For example, the working group thought it likely that genetic changes leading to a loss of susceptibility to the gene drive mechanism will arise rapidly against any candidate construct directed against a single target sequence within either the mosquito or, for population replacement strategies, the parasite, unless it can be shown that changes in those sequences are sufficiently deleterious to prevent them from becoming prominent in the population.

Possible mechanisms that might negatively impact the stability of the effector function in different types of gene drive constructs include the potential for the following:

1. selection for preexisting resistant phenotypes (in the case of population suppression, the concern would be mosquitoes with polymorphisms in the nuclease target sequence that result in resistance^43^; this would also be a concern for population replacement, but in this case selection for malaria parasites that are not killed by the effector mechanism also can be expected);
2. target site mutations to arise spontaneously that make the sequence resistant to the gene drive nuclease or effector mechanism and are positively selected; and,
3. loss of linkage between the effector gene and the gene drive system (this would be a concern for altered function techniques that require the gene drive to carry an effector gene into the mosquito genome, as is the case with some population replacement strategies).

Information about selection for variation in the target gene sequence may be obtained in early studies through multiple small cage experiments, where affected mosquitoes can be captured for genome sequencing.^43^ However, it also will be important to develop effective, practical assays for monitoring resistance in the field.

Mutations that destroy gene drive function in a population suppression strategy are likely to disappear over time because, in this case, a nonfunctioning gene drive will only show Mendelian inheritance and is expected to be outcompeted by a functioning gene drive. However, mutations that destroy only the effector function in a population replacement gene drive strategy that relies on introducing an exogenous effector gene(s), while leaving the drive mechanism intact, are likely to lead to the spread of a nonfunctional construct.^104^ As described by James, “recombination can lead to the loss of linkage of the drive system from the effector gene that it is carrying. Given the large populations of mosquitoes, even rare recombination might be significant. Features must be built into the drive system that decrease this frequency or mitigate its effects if it occurs.”^105^

Research is underway to identify ways to limit target site resistance.^106^ For example, a “combination therapy” approach aimed against multiple target genes and multiple sequences within each target gene could help to delay the onset of resistance to a gene drive mechanism. This might be achieved, for example, by integrating multiple constructs into one gene drive mosquito lineage or by releasing multiple gene drive mosquito lineages each containing a single construct.^41,107^ However, just as for drugs and pesticides, it is likely that given sufficient time, resistance to the gene drive mechanism eventually will arise. The goal should be to stave off resistance for a period of time that is sufficient to halt malaria transmission, which, as predicted by modeling, should take place within a few years after release of gene drive mosquitoes,^13^ although this interval may include a substantial number of mosquito generations. If resistance arises before malaria transmission is halted, a next generation transgenic mosquito product could be released as is carried out with drugs and pesticides.

To be prepared for the possibility of resistance evolving, research on the development of next generation constructs should continue until malaria is eradicated and/or the gene drive product is no longer needed.

### Containment requirements.

For low-threshold gene drives, such as those that can be created using the CRISPR/Cas system,^11,12^ computer simulations suggest that escape of low numbers of mosquitoes from physical confinement in the absence of effective remediation can result in local establishment (P. A. Welkhoff, personal communication).^9,10^ Therefore, physical confinement must be robust, taking a systems approach to containment employing multiply redundant procedures that each provide an additional level of security.

Researchers should be prepared to provide a plan for risk management, including avoiding accidental escape of transgenic mosquitoes, as part of their applications for physically confined testing. This would be facilitated by international harmonization of standards for the minimum containment requirements for gene drive mosquitoes. Sponsors of gene drive research should work with the WHO and other relevant authorities in these harmonization activities. Those overseeing this research at all levels, including funders and regulators, should agree on best practices for the operation of laboratories/insectaries developing gene drive mosquitoes and implement these across all testing sites. Demonstration of adherence to a set of widely recognized standards will enhance public confidence and may help facilities to obtain liability insurance, if required under national laws.

The working group concurred with the recommendations of Benedict et al.,^86^ which describe enhanced Arthropod Containment Level (ACL) 2, or “ACL 2+,” containment and management measures in the laboratory and insectary for mosquitoes** modified with low-threshold gene drive (see Box 6).

Box 6 **Enhanced ACL2 recommendations for containment and maintenance of mosquitoes modified with low-threshold gene drive**

These recommendations build on commonalities in prior guidance for Level 2 and 3 containments of arthropods,^109^ which require well-sealed structures including windows and doors and often call for additional features such as sealed floors, absence of harborages, impervious casework etc. All prior guidance also requires devitalization of arthropods at all life stages before disposal and controlled waste streams. Further measures recommended to ensure containment of low-threshold driving transgenes include:

 More stringent physical containment measures including triple nested barrier containment, but not microbe-specific measures such as high efficiency particulate air filtration and negative air pressure that are often recommended at ACL 3 (it is suggested that 100 μm screens should be sufficient to contain mosquitoes);

 Regular authentication of all strains held, to ensure that driving transgenes have not contaminated other strains in the insectary;

 Use of a distinguishing transgene marker specific to driving transgene when possible;

 Development of diagnostic polymerase chain reaction (PCR) assays for strains containing driving transgenes;

 Thorough inspection of mosquitoes being shipped out of the insectary to ensure they do not contain a driving transgene unless this is intended.

It should be noted that containment and management requirements for self-limiting alternatives must be considered on a case-by-case basis with appropriate risk assessment, recognizing that some of the approaches under consideration will pose similar challenges.

The working group agreed that compliance with international standards for quality assurance of data generation as is normal procedure for biocontainment (e.g., development of and compliance with SOPs,^110^ including strict documentation and record keeping) is necessary and sufficient for maintaining biosafety and data quality, and recommended that Good Laboratory Practice (GLP) certification of facilities is not a requirement. If researchers plan to use their data for product registration purposes in the future, however, independent external audits for compliance with quality assurance standards is advisable to ensure broad acceptability of results. The working group noted that certain assays, such as toxicity studies, are likely to be required as a component of risk assessment,^111,112^ and that such studies are usually contracted to GLP-certified providers; which would be consistent with requirements for pesticide testing.

Regulators will establish the requirement for appropriate biocontainment of gene drive mosquitoes and will determine a method for auditing adherence. Verification of containment must be performed to ensure that all approved practices are being implemented including facility operation and staff training. Currently, verification activities depend on the national and institutional requirements and will vary between sites. For example, in Australia, the Federal Regulator (equivalent to the United States Department of Agriculture) assesses compliance of a facility to the requirements of a given level of containment. Random unannounced inspections are then conducted to check on compliance. Institutional biosafety committees are also charged with ensuring compliance, but they are still beneath the authority of the regulator and the inspection regimen to ensure compliance.

Projects working on investigational gene drive products should ensure that processes for containment and verification do not create conflicts of interest. Therefore, even if strict procedures are in place within the project, compliance with containment requirements should be externally validated. Some locations in which gene drives may be produced or tested may not have a regulatory infrastructure with compliance oversight procedures in place. Even if such procedures exist, they may lack familiarity with requirements for mosquito containment. In that case, third-party validation would be advisable. Thought should be given to the need for an informal or formal certification mechanism for containment facilities housing gene drive mosquitoes.

Breaches of containment could result from unexpected situations, such as natural disasters, accidents, or deliberate actions. Containment considerations must take into account the possibility of facility break-ins, either by opponents of gene drive technology wishing to disrupt the research or alternatively by proponents wishing to gain access to gene drive mosquitoes and release them prematurely. This requires that appropriate security measures are put in place. The potential for an inward breach of containment, involving the introduction of living organisms (including pathogens) from the external environment, should also be recognized, guarded against, and monitored.

Containment considerations for gene drive mosquitoes must extend to transport at any testing phase, to mitigate against escapes at that level. Shipments should use international standards for shipping of medical specimens and GMO, with multiple layers of containment. Shipment and transport as eggs will decrease the likelihood of escape. Permits for transport may be needed, and it can be anticipated that inspection at arrival and a record of the chain of custody will be required by government inspectors.

### Monitoring.

In areas compatible with establishment of the targeted vector species, researchers will likely be asked about methods for monitoring to detect an accidental, unauthorized release of mosquitoes into the environment as part of their risk management plans. At a minimum, monitoring approaches should include efficient traps, mosquito marking methods (e.g., protein markers or genetic markers) and an SOP for conducting surveillance within and outside the facilities. During initial development, researchers should consider the utility of including a specific signature sequence that would easily identify the product as theirs, which could be useful during large-scale field testing and post-implementation monitoring. Multiple trapping techniques should be deployed, using the best technology available at the time, as part of a surveillance effort that targets both adult and aquatic stages of the mosquitoes. Monitoring will be simplified by the inclusion of a unique visual or easily identified molecular marker in the construct.

Development of more sensitive monitoring tools and approaches should be considered by researchers and funders as a critical research priority. Current trapping methods, which could be applicable for monitoring for escapees in the external environment, are of low efficiency. This can be addressed in part by normal procedures for monitoring within and near the facility. If possible, modifications to the external environment around the facility, such as construction of a buffer zone depleted of natural breeding and resting sites, may improve the chances of detecting escapees.

Given the current sensitivity of trapping methods, for physically confined studies conducted in locations hospitable to the vector species detection of a single gene drive mosquito external to the facility should be considered to represent a lack of containment. Surveillance should continue beyond the period in which active research is underway. Because the driving construct will be designed to spread within the local mosquito population, ongoing evidence of the absence of local establishment will provide confirmation of the validity of containment procedures. When physically confined studies are conducted in areas where gene drive mosquitoes will be unable to survive, less stringent ongoing and posttrial monitoring requirements are appropriate.

It is important to be aware that for studies with gene drive mosquitoes, others unaffiliated with the project also may be monitoring for escapees. A strategy should be put in place for responding to such monitoring efforts and the possible outcome that detection of escapees is claimed. This might include providing a mechanism for transparent information sharing of monitoring results and/or for periodic independent testing of trap contents. A comprehensive communication plan also is necessary as part of overall public engagement, as discussed in the following section.

### Engagement.

As described in the WHO Guidance Framework, researchers have different engagement obligations to the community(ies) at the research site as compared with the greater public.^15^ At the phase of physical confinement, the focus will be on in-country engagement. Engagement during contained studies conducted within the laboratory and/or insectary provides an opportunity to explain project goals and operations, develop a relationship with the community, and initiate the process of building trust. Community engagement is best managed and undertaken by in-country social scientists who understand local value systems and can easily interface with the people. While still working in the laboratory or insectary, researchers should consider their obligations to the community in the immediate vicinity of the facility but should also be planning their interactions with the larger public. At each subsequent phase of testing, broader outreach and engagement will be required.^15^

Engagement during contained studies in malaria-endemic countries should be considered as the beginning of a continuum that will carry on and expand through the remaining development and testing process. Researchers should be prepared to treat engagement as an ongoing process, providing options for ongoing dissemination of information about the project and discourse with the community. This might involve, for example, establishment of a community liaison group or conduct of “open days” at the research facility where community members can observe the work and talk directly with researchers.

Because collection of baseline field data must begin well before releases are contemplated, the start of field population studies will represent another early community engagement opportunity. Baseline studies at the planned release site will require the presence of a field team in villages at the study site and may involve local community inhabitants, for example, for their knowledge of mosquito breeding sites or as collectors. It will be essential to obtain community support even before starting baseline studies. Engagement interactions need to be initiated well before beginning the field studies if the field team is not known to the community and there is a need to build familiarity and trust. Timing could be altered if the study site and population are already familiar with the study team.

Engagement activities at this phase should include consultation with government authorities to understand their needs and requirements for a malaria control tool. This might include discussions with the national malaria control program, which would be expected to use the gene drive mosquitoes as a tool for malaria prevention. However, researchers are cautioned not to assume that engagement with government authorities, including regulators, is a substitute for active community engagement.

Engagement activities should be supported by a comprehensive communications plan, which will underpin efforts to inform society about the project in good faith. Researchers are encouraged to develop their communications plan in consultation with experts, who will be skilled at developing understandable communication approaches that can reach stakeholders at various levels. The working group members remind researchers of their responsibility to engage and communicate clearly and accurately about the potential risks and benefits inherent in their work, while not creating misperceptions.

### Regulatory issues.

Researchers should expect that regulators will want to understand remediation options in the case of an accidental release, which must be communicated in a realistic manner. Researchers and funders also should be aware that regulators may require a plan for follow-up surveillance after completion of approved physically confined studies. It is possible that this may extend for a prolonged period. Expectations may be clarified by early interaction with regulators and ministries or other parties responsible for malaria or biosafety surveillance. Researchers and funders must be prepared to support remediation and surveillance activities as required by regulators.

The Cartagena Protocol currently does not require notification of neighboring countries for physically confined studies with GMO. However, Article 17 does require countries where a release may lead to an unintentional transboundary movement that might have an adverse effect on biodiversity to notify other countries that might be affected; this requirement could become relevant in the case of a breach of laboratory containment in countries where the species is endemic, and the modification is likely to become established and spread within the natural mosquito population. Researchers must notify the NBA of a breach and follow the instructions from the regulator. The country, through its designated Cartagena Protocol Focal Point,^113^ is responsible for notifying other countries.

### Key points: physically confined laboratory studies.

- Strict physical confinement of gene drive mosquitoes can be achieved in an indoor laboratory, insectary, or large environmental chamber. It is expected that a larger testing space will provide a more realistic sense of mosquito behaviors such as mating competitiveness.
- All studies of mosquitoes carrying a low-threshold gene drive system before approval to move to field testing should be conducted under “enhanced Level 2 containment,” equivalent to Level 3 conditions for arthropods without the Level 3 requirements for pathogen containment. Development of SOPs and training of personnel on compliance will be critical to maintenance of containment and data quality. Those overseeing this research should agree on a set of widely recognized standards for operation of laboratories/insectaries testing gene drive mosquitoes. Compliance with containment requirements should be externally validated.
- If an appropriate containment facility and well-trained staff are not available within the endemic area partner country, or risk of escape from containment cannot be brought to acceptable levels for other reasons, then initial safety work with driving constructs should be performed outside of a region suitable for establishment but in mosquitoes with the local African genetic background. Scientists from African partner institutions should be involved in the development that takes place outside the region.
- Candidates considered for movement to field testing should be based on a sound hypothesis and demonstrate efficacy and fitness characteristics in the laboratory that are consistent with the TPP and anticipated product claim. At this early stage of testing, the major efficacy criterion that should prevent a gene drive candidate from being considered as a potential vector control tool is the likelihood of resistance arising too rapidly for the candidate to have a beneficial effect against malaria transmission, and every effort must be made during initial laboratory studies to mitigate against this possibility.
- As is the case with conventional biocontrol agents, emphasis must be placed on safety evaluation before the initiation of field releases. All aspects of safety identified by risk assessment should be investigated as thoroughly as possible under appropriate physical containment to justify moving a candidate to field testing. For example, potential harm to human and animal health, or to biodiversity, should be considered. The hazards posed by a proposed release need to be assessed and their probabilities judged as acceptably low by the regulatory authority based on laboratory data before the investigational product is moved from the containment facility to the field.
- The standard for moving a gene drive construct from the laboratory to field testing should be a well-reasoned justification that it will do no more harm to human health than wild-type mosquitoes of the same genetic background and no more harm to the ecosystem than other standard vector control interventions.

## SITE SELECTION AND PREPARATION FOR FIELD-TESTING

Although making no assumptions about where gene drive constructs might be created, working group members recognized the critical importance of site selection and preparation for field testing gene drive mosquitoes. For those projects originating outside a disease-endemic country, this will begin by establishing a partnership with an institution with which to pursue research and product development within a country where further testing will be conducted.

Regardless of where the project originates, it will be necessary to identify and characterize potential sites for field releases. Site selection must begin very early in the development pathway, ideally more than 2 years before any releases are contemplated, keeping in mind the need for substantial vector, disease, and relevant ecological baseline data and community engagement before field-testing. Thus, site selection and preparation ideally will commence during the period following initial proof of principal, but while the gene drive investigational product still is being tested and refined in the laboratory. Guidance on selection of sites for field-testing GMM has been published elsewhere.^15,114^

### Partnership and technology transfer.

In situations where the gene drive construct is developed outside of sub-Saharan Africa, and researchers are seeking an African partner institution for continued research and development, technology transfer should be a major goal of the work. Partnerships with researchers and institutions in the country(ies) where the product will be developed and deployed must be conducted in a spirit of co-ownership and co-development of the technology, and in a manner that will promote and foster leadership by the in-country scientists. Scientists and research institutions in the countries where the product ultimately will be used must play a central role in the development process from its early stages. In addition to their key scientific role, they will be the most appropriate group to present the technology to communities and other stakeholders in their countries. In-country researchers will be best positioned to understand in-country attitudes and to build trust in the research. They also will have access to local genetic variants of *An. gambiae* and locally important vector species other than *An. gambiae* to which the technology will need to be applied to achieve malaria elimination on the continent. Although all partners should be available for support, the in-country institution will bear responsibility for interactions with the national regulatory and other government authorities. In fact, many countries require partnerships with national institutions, with the in-country institution as the applicant organization, as a condition of regulatory approval for any activity involving a GMO. Thus, the presence of an in-country champion for the development of a gene drive product is imperative.

Technical capacity at the in-country partner site is an important consideration for choice of partner; for example, adhering carefully to SOPs and institutional policies is a prerequisite for assessing and ensuring both safety and efficacy of the gene drive mosquitoes. The in-country partner institution should have access to sufficient infrastructure to support field trials, including an experienced team of entomologists and epidemiologists, and the capacity for transport, sample collection, and laboratory work. Participation of social scientists and science communication experts also will be required before the initiation of field trials. If not already present, support for appropriate infrastructure and capacity strengthening will be required.

### Political support and in-country acceptability.

Recognizing that in sub-Saharan Africa many research organizations are government affiliated, researchers will be responsible for undertaking the early discussions with appropriate authorities and others that are needed to gauge properly their willingness to consider the development and deployment of gene drive technology. Strong evidence of political will to enable and support gene drive studies is a critical element of site selection. Political stability is a highly desirable, but not always predictable, site characteristic.

Early interaction with the Ministry of Health or equivalent is important, because this agency would be the ultimate consumer of the technology as a public health tool. Likewise, early interaction with the appropriate regulatory bodies, which in some countries may be local and/or regional as well as national, is critical. Any existing international agreements that the country has ratified should be considered. In the case of gene drive mosquitoes, the Cartagena Protocol on Biosafety^82^ is relevant, and laws governing research and development of GMO are anticipated to provide an overarching framework for regulatory decisions. Typically, these will be placed in such governmental units as the Ministries of Research, Science and Technology, or Environment. However, other relevant ministries such as Health also should be involved in the decision-making process, and health regulators will play a crucial role in later stage testing.

Civil society often plays a critical role in shaping national discussions. It would be wise to investigate local social and cultural perspectives on biotechnology research, malaria eradication, and large-scale public health efforts. Stakeholder mapping^67^ will help to identify influential religious groups, nongovernmental organizations or individuals who should be engaged. It also will be important to determine whether there are any concerns about protection of local biodiversity. This would include species that are locally valued, as well as species that are protected by national and international conservation laws or agreements.

### Legal and regulatory infrastructure.

The presence of a national biosafety law, or other relevant legislation, and functional regulatory infrastructure should be considered a priority in the selection of research sites. Not all regulatory systems are alike, and it is important to determine in advance whether the regulatory authorities are open to GMO and willing to engage in constructive discussions on this issue. Relevant laws regarding liability and redress for personal injury or environmental damage should be considered in site selection. Where strong precautionary principles underlie the existing regulatory framework, it will be important to engage early with authorities in a discussion of whether they consider the relevance of potential benefits as well as risks.

Because of the potential for transboundary movement, such risk–benefit discussions should consider the possibility for regional regulatory cooperation in planning and conduct of early testing. If there are regional authorities that support multiple countries, these should be consulted as well to determine their understanding of and interest in the use of gene drive mosquitoes for malaria prevention. Early discussions with regulators and other government authorities would be facilitated by access to regulatory and communications advisors and external technical experts in areas that are not represented on the research team. As mentioned in the following text, regulatory capacity building may need to receive substantial attention in some disease-endemic countries.

### Field site characteristics.

Researchers will be responsible for taking all the various requirements for conducting a successful test into consideration and justifying that plan to regulators. The site(s) where efficacy trials will be performed must have, ideally as the predominant malaria vector, the same mosquito species that is targeted by the investigational gene drive mosquito product. This will be particularly important for determining epidemiological impact. Moreover, field site(s) for initial releases must be chosen with a view to geographic isolation (see **Small-scale field testing for entomological efficacy**).

Consideration must be given to anticipated scale and outcomes when selecting sites for future trials. At trial sites, researchers will require access to current baseline data on entomological and epidemiological factors of relevance to trial design and endpoints. The working group suggested that such data should be available for at least 2 years before initiation of testing to allow for observation of multi-seasonal and multi-year variation. Researchers must anticipate the conditions under which efficacy trials ultimately will be conducted, particularly with respect to what other vector control methods might be in use, and ensure that those interventions are in use during the collection of baseline data.

Data to be collected will be those that are necessary to assess the effects of the investigational gene drive product on malaria vector mosquito populations and on local parasite transmission. These might include information such as vector diversity and population structure, effective vector population size, vector dispersal, sporozoite rate, and natural variations in the genomic sequence(s) that are targeted by the gene drive construct. Genetic characterization of the vector may be important for proper targeting of gene drive constructs. If effect on malaria transmission will be assessed in the trial, data should be collected on malaria incidence and prevalence, and on the distribution of the parasite across different *Anopheles* species. Even in early testing, in addition to determining that the relevant mosquito species is present at the proposed field site, regulators also may want to know the epidemiological significance of the specific vector that the gene drive targets. It may be useful to engage with an expert in the design of large-scale vector control trials to plan more specifically what information will be needed for future trials of the investigational product early in the development process. Experiments at all phases of testing should be designed to address anticipated questions about risk; in this regard, it will be necessary to solicit input from experienced ecologists. Researchers should be mindful of the potential need to collect relevant baseline ecological data, in addition to the focus on mosquitoes and malaria.

Researchers should consider collecting, cataloging, and storing samples of local mosquitoes and malaria parasites as a genetic repository against which they can measure any changes resulting from field-testing of gene drive mosquitoes in the future. This would require standardized methods for species identification and appropriate facilities for archiving samples to maintain their integrity. Such sampling ideally would be done in multiple areas, which will eventually be split between control and treatment sites (see sections on *Efficacy* testing).

### Key points: site selection and preparation for field testing.

- When investigational gene drive products are created outside the region where they will be field-tested, partnership with in-country scientists and institution(s) must begin early in the development process.
- Partnerships must be conducted in a spirit of co-ownership and co-development of the technology, which promotes and fosters leadership by the in-country scientists. They will play a pivotal technical and representational role throughout the development pathway.
- Identification of partner organizations and field sites should take into consideration not only the level of in-country scientific capacity and local malaria transmission conditions, but also whether there is political support for new technologies and a national biosafety law or other relevant legislation that would provide the necessary regulatory infrastructure for evaluating the technology.
- Selection of field sites must begin early enough to obtain multi-seasonal baseline entomological, epidemiological, and ecological data needed to plan future trials.

## SEMI-FIELD TESTING

The intent of a phased development pathway is to test a new technology incrementally, adding complexity at each new step. For other GMO, limited environmental exposure traditionally has been accomplished by two means: physical or ecological confinement. These methods may likewise be applied to gene drive mosquitoes. Thus, the first step beyond contained laboratory testing for GMM may be conduct of physically confined semi-field, or caged, testing. Semi-field testing is intended to allow for observation under a more natural setting, but under conditions that limit release of gene drive mosquitoes into the environment.

This step was not stated as a requirement in the WHO Guidance Framework and was not considered by this working group to be on the critical path for development from a technical perspective (see Figure 1). Nevertheless, the working group acknowledged that semi-field testing within large outdoor cages may be viewed by regulators, communities, and/or the public as important for maximizing information about or familiarity with gene drive mosquitoes before field release, perhaps especially when testing a first-in-class approach. Thus, it will be important to have a discussion about the value and necessity of semi-field testing with regulators and the public early in the planning process. In the case that semi-field testing is pursued, it will be important to use it as productively as possible and suggestions on how to do this are provided in the following text. In making the decision as to whether semi-field testing will be undertaken, it must be acknowledged that semi-field trials add time and cost to the development pathway for gene drive mosquitoes as a tool for malaria control and eradication.

Information similar to that expected from semi-field testing also may be obtained in indoor facilities outside *An. gambiae* hospitable regions by testing in large environmentally controlled indoor chambers that simulate natural temperature, humidity, lighting, and spatial conditions. In these recommendations, such facilities are considered as part of physically confined laboratory testing. As an extension of laboratory testing, such facilities provide several layers of containment and offer a useful alternative or precursor to semi-field testing in the mosquito-hospitable region for evaluating the behavior and fitness of gene drive mosquitoes.

The recommendations of this working group consider only the requirements for semi-field testing in an environment hospitable to *An. gambiae*. In malaria-endemic settings, this is assumed to involve testing in outdoor cages.^95,115,116^ This phase is not intended to involve testing in a field facility that provides an ACL 2 + laboratory level of containment. The intent of semi-field testing is to create a nearly natural environment in terms of exposure to variations in environmental such as light and weather and natural flora and fauna. Thus, the working group recommended that it is not useful or desirable to build high containment field cages in disease endemic regions solely for testing gene drive mosquitoes. It must be understood, however, that the chances for gene drive mosquitoes to escape into the environment will be higher in semi-field testing than in the laboratory. Also, no matter how much effort is put into making the cage environment as natural as possible, it still presents a protected and artificial environment. Thus, results from cage testing may not be entirely reflective of results from a field release.

The working group did not consider semi-field testing to be an essential requirement; however, if such testing is conducted, the aim should be to improve the likelihood for success of a subsequent field release. In this regard, the working group envisioned the possibility of a dynamic bidirectional interaction between semi-field studies and initial small-scale release studies. For example, it could be useful to return to cage testing to obtain additional information on mosquito performance to refine methodology and improve the delivery regimen if the first release does not result in satisfactory establishment.

### Safety.

Because containment cannot be guaranteed in semi-field testing, and gene drive mosquitoes have the potential to become established in the environment from low-level releases^9,10^ (P. A. Welkhoff, personal communication) (see Box 7), the determination whether to proceed from physical confinement in the laboratory or insectary to the field is a critical decision point. Semi-field testing should not be considered as a prerequisite for decision-making on safety before field release.

Although regulators will conduct their own risk assessment, the working group strongly recommended that an external, third party all-hazards risk assessment be conducted before decision-making about a regulatory application, as a tool to guide planning and preparedness. As discussed previously, biosafety hazards identified in risk assessment must be addressed satisfactorily before any transition from the containment facility to semi-field testing. The recommended standard for moving a gene drive construct from physical confinement in the laboratory or insectary to semi-field testing should be a well-reasoned justification that it will do no more harm to human health than wild-type mosquitoes of the same genetic background and no more harm to the ecosystem than other standard vector control interventions (which include broad spectrum insecticides).

Box 7 **The hazard of establishment**

The hazard of establishment addresses the possibility that following an accidental release the gene drive construct will still exist anywhere in the environment months to years later. For low-threshold gene drives a variety of analyses predict that release of low numbers of mosquitoes could result in a greater than 50% chance of establishment. However, the probability of establishment calculation does not state anything about how many mosquitoes carrying the gene drive construct remain in the environment, how dispersed they will be, or whether they will drive to fixation around the release site. It only indicates that there is a higher likelihood that the construct will not have disappeared.

Because of the possibility of loss of containment from outdoor field cages, the working group recommended the conservative position of regarding semi-field testing as synonymous with small-scale release. Risk assessment at this point should include small-scale field release, but also should consider whether any additional hazards are created in the context of cage testing (for example, escape of a concentration of mosquitoes). Risk assessment for decision-making to move to field testing will be based on safety and efficacy data collected in containment, including any information obtained from testing in large environmental chambers if this is applicable, and predictive analysis of these data including modeling. If indicated by risk assessment, monitoring of effects on nontarget species would most feasibly focus on a subset of “sentinel” organisms identified on the basis of their potential for interaction with gene drive mosquitoes in the field cage.^95^ Ecologists should be consulted in this decision. Semi-field testing could provide an opportunity to confirm prior safety results in the locally relevant environment.

### Efficacy.

Semi-field testing can provide additional information on the local performance of gene drive mosquitoes that could help in the design of future efficacy trials. Most of the information to be gained in such studies likely can be obtained by short studies over just a few generations. This will include insights on the rate of spread of the transgene under different conditions. Semi-field testing also might provide preliminary information on mating competitiveness and assortative mating, including spread of the transgene to other members of the *An. gambiae* species complex that are present at the site and might be introduced into the cage.

Semi-field testing offers a further opportunity to confirm efficacy of the gene drive construct in the wild genetic background. For population suppression strategies, this would allow testing the rate of suppression against wild mosquito isolates under larger scale conditions. For population replacement strategies, it may be necessary to bring gene drive mosquitoes back to the laboratory to assess their ability to prevent development of local *Plasmodium* isolates using membrane feeding methods with fresh gametocytes.

Certain behavioral characteristics might be examined within the field cage. For example, it might also be possible to obtain a sense of how the gene drive mosquitoes interact with other vector control methods, such as long-lasting insecticidal bed nets. Although likely to be logistically difficult and time-consuming, if cage testing were to be conducted over different seasons, it might provide preliminary information on dry season survival that could be useful for design of future efficacy trials. Depending on the nature of the proposed method, semi-field testing might support proof of concept for a potential genetic remediation strategy; however, if the remediation construct includes gene drive, its premature release and establishment could jeopardize further testing of the original investigational product.

Semi-field testing is not expected to provide any direct data on epidemiological impact, although it could provide data that refine parameters for modeled estimates of potential impact.

### Site selection and containment requirements.

Standard location, and structural and operational characteristics of semi-field cages have been described in detail elsewhere,^95^ where double-walled design was recommended with mesh or screen sides and ceiling of a porosity suitable for containing mosquitoes (100 μm netting was suggested). If regulators allow semi-field testing to be conducted under the same authorization as small-scale release (see *Regulatory issues*), the stringency of containment requirements for field cages can be balanced with the need to provide near natural conditions that will increase the relevance of results to later field testing efforts. Cage size is relevant because the intent is to simulate a natural outdoor environment and allow near-natural mosquito biology and behavior, such as formation of mating swarms. Larger cages also can accommodate the introduction of native plants or alternative animal hosts to enhance the environment and allow staff to work inside.

Given the possibility of escapes, if semi-field testing is deemed to be appropriate, researchers and funders should consider the desirability of placing the field cage in a geographically isolated location as described for small-scale field testing (see **Small-scale field-testing for entomological efficacy**). Compromises between ideals for physical and ecological confinement, as well as practical issues such as worker access, must be considered in determining the best location for the cage.

As in physically confined laboratory testing, adherence to SOPs for containment, record keeping, and other processes of quality assurance will be crucial for studies conducted in the field cage. Given the outdoor location of semi-field cages, SOPs also should be put in place for responding to unexpected events, such as acts of nature that might compromise containment. Staff training and auditing for compliance with such procedures will be a priority in preparing for semi-field testing. Security precautions should be put in place because the field cage will present an obvious target for opponents of the technology wishing to halt the research, or alternatively, for proponents wishing to gain access to gene drive mosquitoes and release them prematurely with the hope of preventing malaria.

### Monitoring.

Regulators will conduct their own facility and trial monitoring. However, the working group suggested that provision for independent third-party monitoring during semi-field testing could be useful for affording both additional information to the project and reassurance to the public.

Standard operating procedures should include plans for monitoring for gene drive mosquitoes outside the cage, similar to those implemented during physically confined laboratory testing. Response to detection of escapees will be dictated by whether regulatory permission for small-scale release has been obtained (see *Regulatory issues*). If the cage is located in or near the intended site for initial small-scale release, and approvals for such a release already have been obtained, escapes from the field cage might simply be considered as the beginning of the planned transition to small-scale release. However, if the field cage is not located in the planned site of small-scale release and/or there has been no approval for release, then escapes from the cage that may result in local establishment of the transgenic mosquitoes would trigger a previously agreed on follow-up plan. Before semi-field testing in this scenario, researchers should have developed a strategy for monitoring for persistence and establishment, along with any remediation activities, in agreement with regulators. Because it is expected that safety will already have been carefully assessed in laboratory testing, remediation may not be a major concern if low-level escapes are detected. However, in a situation where accidental or intended releases have occurred, monitoring for establishment of the gene drive construct in the local *An. gambiae* population should be conducted before subsequent field releases at the same site to ensure the population still will be susceptible to the gene drive mechanism.

### Engagement.

It will be crucial to assess community attitude toward the project before committing to a trial site. Semi-field testing provides additional opportunities for further engagement with the local community. Before and during semi-field testing, engagement activities will include stakeholders living in the region of the field cage and potential release site(s). Although interaction with key opinion leaders should have begun earlier, it will become a critical component of engagement at this point.

It will be important to ensure that the community understands the concept and goals of semi-field testing, including information on safety studies that have already been performed. Observation of research staff working inside the field cage with gene drive mosquitoes may help to boost public confidence about safety. However, researchers will need to explain that cage trials are not aimed at evaluating safety but are for obtaining information that will optimize planning for field release. The community must be advised of the possibility that escapes may happen and what will be done if that should be the case. The potential need for repeated cage studies also should be explained. A process must be put in place sharing information on results from these and other relevant studies with the community and responding to their questions or concerns.

Researchers must identify an appropriate method for obtaining community endorsement to conduct the studies. What constitutes acceptance will be culturally determined and may be identified through advance discussions with the community. Engagement is an ongoing process, and community authorization/endorsement must be continually confirmed as new studies are undertaken. Institutional ethics committees, regulators, and community advisory boards likely will play a role in defining the requirements for authorization. If studies involving human research participation are conducted within the field cage, appropriate ethical approvals must be obtained.^65^

### Regulatory issues.

Individual countries will be responsible for regulatory decision-making. However, researchers also are strongly encouraged to interact with the WHO through the VCAG before proceeding to field studies in locations hospitable to the target vector species.

Because containment within semi-field cages cannot be guaranteed, the working group suggested that if semi-field testing is undertaken the regulatory application optimally would request permission for both semi-field testing and small-scale field release, rather than semi-field testing alone. Safety justification for such a combined application will be based on data collected during contained studies, including, if applicable, data from testing in large environmental chambers located in regions inhospitable to *An. gambiae*. Because the application usually requires information on geographic area and local conditions, the regulatory process will be simplified if the cages for semi-field testing are located in the same area where initial small-scale releases are planned.

Researchers, funders, and government authorities should agree on any remediation expectations and accountability before the initiation of semi-field testing. Plans should indicate how residual populations in the cage will be eliminated after a trial is completed, if required by regulators. The current regulatory assumption, based on experience with confined trials of GM crops, is that the modified organism will be removed from the environment after an approved period. Regulatory recertification may need to be sought if the research continues beyond the originally approved timeline.

If semi-field testing is conducted, the working group recommended that such testing be managed on a regional basis, with involvement of regional regulatory authorities in agreement on issues such as protocol development, testing requirements, and data collection methods. The intent is to facilitate regional understanding and acceptance of the results from semi-field testing, thus increasing the relevance of the results while reducing the need to build cages and repeat testing in every country.

### Key points: semi-field testing.

- Semi-field testing is intended to allow initial field evaluation in a more natural setting than the laboratory, whereas still limiting environmental exposure. The aim of semi-field testing should be to improve the likelihood for success of a subsequent field release. Conduct of semi-field testing also may serve to boost regulatory and public confidence in the investigational product. Because a safety determination should have been made previously based on studies conducted under physical containment and performance results obtained within the protected cage environment, although potentially informative, may not be entirely reflective of results from a field release, caged testing was not considered to be on the critical path for development.
- Mosquitoes carrying a low-threshold gene drive system theoretically may be able to establish themselves from low-level introductions. Because of the possibility of accidental escape from outdoor field cages, the working group recommended the conservative position of regarding semi-field testing as synonymous with low-level release. If undertaken, cage testing should be conducted with the understanding that it is part of a field testing continuum. If allowed by authorities, the regulatory application should include permission to conduct both semi-field testing and small-scale field release, rather than to conduct semi-field testing alone. It is recommended that the cage be placed in or near the site planned for initial small-scale releases, if possible, to simplify regulatory review.
- Although regulators will conduct their own trial monitoring, researchers or funders should consider arranging for independent third-party monitoring for escapes during semi-field testing, to provide both additional information to the project and reassurance to the public. Plans should be put in place before testing begins about how to follow up if monitoring detects a lack of confinement.
- If semi-field testing is conducted, it should be handled on a regional basis, with regional involvement in and agreement on protocol development, testing requirements, and data collection methods. This is intended to facilitate regional acceptance of the results from semi-field testing, thus increasing the relevance of the results while reducing the need to build cages and repeat testing in every country.

## SMALL-SCALE FIELD-TESTING FOR ENTOMOLOGICAL EFFICACY

As defined in the WHO Guidance Framework, the next testing phase would be small-scale ecologically confined testing with a primary goal of measuring entomological efficacy.^15^ The working group debated whether ecological confinement is applicable to gene drive mosquitoes released into an existing wild population. As described under the Site selection and containment requirements, efforts can be made to identify a geographically isolated site for the initial field release where ecological characteristics minimize the possibility of outward migration of gene drive mosquitoes and inward migration of wild-type mosquitoes. Thus, the working group determined that although in the case of low-threshold gene drive ecological confinement cannot be assured, the intent in the initial field trial should be to minimize environmental exposure to the extent possible while confirming efficacy and safety observations from prior stages of testing.

Trial design for entomological efficacy should include standard elements such as outcome measures, comparators, data collection methods, analysis, and reporting schedules, and stopping/discontinuation rules. The primary technical goals of small-scale field releases are to: 1) test the rate of transmission of the transgenic construct into the local *An. gambiae* population; and 2) continue assessing biological function (suppression or replacement).

It is expected that there will be a need for several small-scale trials, each perhaps on the order of a single village, to understand the effect on entomological endpoints of variables such as delivery protocol. Such small-scale trials will also assess performance under different environmental and transmission conditions before proceeding to large-scale trials testing epidemiological efficacy. The rate of spread of the transgenic construct via mating is likely to be dynamic, influenced by factors such as variation in mosquito density, effects of seasonality, natural barriers to or promoters of dispersal, use of other vector-control interventions, human population density, and mosquito species composition. Thus, researchers, funders, and regulatory authorities should expect and plan for more than one small-scale release to obtain data across a range of transmission conditions as necessary to design a trial to measure disease impact. As discussed in the following text, the more similar the test sites at this phase are to the sites for future epidemiological efficacy trials, the more relevant the results will be for future trial design. Indeed, researchers should consider the possibility that sites chosen for small-scale releases later will become part of the epidemiological trial for that same investigational product. When identifying the locations at which to conduct small-scale testing, researchers should keep in mind that the WHO VCAG has expressed a preference for more than one large-scale efficacy trial, to be conducted in different settings, and consider how this might figure into planning.^32^

Assuming no early evidence of adverse consequences, geographic isolation need not remain an expectation in subsequent small-scale trials following the initial release, but observation for any unanticipated results related to safety should continue. As mentioned, subsequent small-scale trials may need to be conducted in multiple sites and under different malaria transmission conditions. The concept of multiple small releases is similar to the traditional testing pathway for drugs and vaccines, and that proposed for other types of vector control products, which generally rely on multiple small-scale trials to collect all data necessary to design a complex and resource intensive large-scale trials for disease efficacy.^117^

Researchers and regulators must agree on a plan for how long initial releases will be followed and what information is required to make the decision to move to large-scale releases for testing epidemiological efficacy. Modeling will help identify the data that should be collected and how long observation should continue. From the standpoint of information collection, a longer observation period would yield valuable data on duration of efficacy (including how long it takes for genetic resistance to develop) and additional insights on safety (e.g., allowing more time to check for ecological effects). For example, sites of early releases of *Wolbachia*-infected *Aedes aegypti* mosquitoes have been monitored over years to understand the stability of the antiviral effector mechanism.^118,119^ However, a longer term observation period also will increase the probability that mosquitoes could escape from geographic confinement. The time frame will need to balance data needs, regulatory needs, availability of funds, and expediency.

If the site is suitable for inclusion in the trial for epidemiological efficacy, the release can be allowed to continue while releases at additional sites commence. If the site is not expected to participate in the epidemiological trial, the release could still be allowed to remain active for extended observation. If allowed to continue, it is anticipated that long-term follow-up could be conducted in parallel with subsequent testing so as not to add time and costs to the development process. This practice has precedent in vaccine and biocontrol trials. If study termination is required, it is expected that this would involve intense application of standard pesticides.

### Safety.

As stated previously, initial field release should not proceed without an external third party all-hazards risk assessment to determine those risks that are significant and require risk mitigation measures and, thereby, guide planning and maximize preparedness. Although data from physically confined studies will form the basis for a decision about the safety of moving to field releases, it is expected that continued observation for safety will be maintained during small-scale releases as a precaution and regulatory requirement. Ecologists should be involved and/or incorporated into the team at this point. Any specific concerns will be identified through the risk assessment process, depending on the nature of the gene drive construct and the environment where the release will occur. If already performed in the context of semi-field testing, subsequent risk assessment at this phase would be incremental, focusing on differences such as numbers of mosquitoes to be released, trial site, or period of exposure. During small-scale field testing, it is important also to include concerns expressed by community members at the trial site and by involved third parties, such as government officials, in the risk assessment process. Project risks, such as reputational risk to the research institution(s), also must be taken into account. After the initial release, risk assessment for subsequent small-scale releases likewise would be incremental, taking into consideration differences such as site characteristics or numbers of mosquitoes to be released and additional concerns expressed by different communities.

The views of ethics committees and/or regulators will provide the safety information that is required and for how long monitoring should continue. Trials must be designed to address safety questions raised by regulators or other oversight bodies. As an example, due diligence at this phase might involve observation of the stability of the transgenic construct and key interactions of the transgenic mosquitoes with other species. The relevant period for monitoring may differ between population suppression strategies, where numbers of transgenic mosquitoes are intended to decrease sharply over time, and population replacement strategies that aim to maintain transgenic mosquitoes in the environment indefinitely.

Determining an impact on malaria incidence is not expected to be a key objective of early small-scale trials. However, if malaria transmission is present at the site of small-scale releases, then monitoring for human health consequences should be considered as a safety precaution. Data on existing control measures, incidence of cases, and prevalence in various age groups should be collected before and after the release (see *Field site characteristics*). If an adequate passive surveillance system is not available in hospitals and health centers within the study area, there may be a need to bolster or establish such a system to obtain accurate data. This information will be important for evaluating whether there is any correlation between a change in disease pattern (either positive or negative) and the release, and likely will be requested by both the community and regulators as a safety measure.

Evidence for adverse impact on human health would constitute a “no-go” criterion to end further testing and would trigger remediation efforts. Even at this early stage, researchers are encouraged to consider the utility of a small Data and Safety Monitoring Board (DSMB),^120^ as is common for clinical trials, to provide independent monitoring of the malaria data, to avoid bias in decision-making. An independent trial steering committee would be helpful to provide advice on the many decisions to be made in field testing.

### Efficacy.

It will be important to plan the first introduction of gene drive mosquitoes to provide the best opportunity for a successful trial. Modeling based on the specific characteristics of the investigational gene drive product and current entomological and epidemiological data from the field site location will be instrumental in designing the initial trial. However, allowance should be made for the fact that field performance of vector control tools is rarely as good as predicted in theory based on limited biological information.

Proof-of-principle testing will determine whether the construct performs as expected, that is, whether it increases in frequency within the local *An. gambiae* population over time and maintains its biological function. A major objective will be to understand movement of the driving transgenes through the local mosquito population. Preliminary insights may be gained at this phase about how environmental conditions and human population distribution affect the rate of spread. An initial understanding of these characteristics can be obtained at any location where *An. gambiae* are present, but it will be important that baseline studies have been conducted for a sufficient period to accurately assess them. This information on rate of spread will be crucial for design of subsequent trials for epidemiological efficacy. For example, for a randomized, cluster-controlled trial (described under Epidemiological testing), it will be important to be able to predict how long it will take to observe the effect in mosquitoes throughout a treatment cluster and how much separation between clusters will be required. Because these characteristics are expected to be influenced by local conditions, it will be necessary to conduct a small-scale release in an environment that is similar to the one envisioned for the epidemiological efficacy trial(s). In locations where seasonality is an issue, monitoring for transgene spread must continue across the dry season. Transgene tracking should make use of available life stages, including larvae, adult swarms, and females (including spermathecae).

The other major category of performance information needed to design an epidemiological efficacy trial is functional activity (entomological efficacy). For population suppression strategies, entomological efficacy can be determined by testing for transgene prevalence and reduction of *An. gambiae* within the local mosquito population by trapping or other collection methods. Although localized elimination may be desirable, this could be difficult to achieve under conditions of small-scale release if there is potential for inward migration of *An. gambiae* from surrounding untreated areas. Care must be taken to ensure that any relevant environmental changes, such as diminished rainfall, are taken into account in interpretation of the entomological results. Testing entomological efficacy for population replacement strategies will require assessment of the ability to sustain development of local *Plasmodium* isolates by calculating the proportion of mosquitoes that are infected. Depending on the conditions of malaria transmission at the site, this may be accomplished by looking at naturally acquired infection rate or by membrane feeding with fresh gametocytes in the laboratory. In either case, mosquitoes will need to be brought back to a laboratory facility for testing. Thus, accessibility of the release site will be a particularly important consideration for assessing function of population replacement strategies.

Resilience to the development of genetic resistance should be assessed to the extent possible within the time frame of the small-scale release. Development of resistance can be assessed by observing the stability of functional activity and by periodic sampling and molecular characterization of the local *An. gambiae* population to look for variations in the gene drive–targeted sequence. For population suppression, resistance might be detected as a reversal of the trend toward decreasing numbers of *An. gambiae*.^43^ For population replacement, the goal is complete inhibition of parasite development.^11,13,121,122^ If complete refractoriness is not observed, it will be important to monitor for development of parasites that are resistant to the effector mechanism and for evolution of genetic resistance to the gene drive construct in the mosquitoes. If resistant parasites are observed, it will then be necessary to consider whether the changes might make them more dangerous (e.g., more virulent, or more resistant to antimalarial drugs).

Other useful data that can be collected at this stage, if the release is allowed to proceed long enough, might include effect on other species within the *An. gambiae* complex (assuming other species are present); changes in overall malaria vector abundance, composition, or age structure over time; and changes in *Plasmodium* infection rates in the vector (assuming malaria transmission at the site).

Performance should be judged against defined criteria for adequate behavior (e.g., establishment, spread, functionality), which should be established before release within a TPP discussed with the regulatory authorities (see **General considerations for developing gene drive mosquitoes**). Inference can be drawn about the adequacy of the investigational gene drive product, based on whether the key performance targets were reached and the predicted study objectives were achieved. For each small-scale release, researchers and regulators must agree on the timepoint(s) for reporting results and for deciding whether to move forward to large-scale releases, irrespective of whether the release is terminated. Indeed, in keeping with the concept of a field testing continuum (Figure 3), large-scale trials may involve an expansion from the site(s) of small-scale release.

As mentioned, testing epidemiological efficacy is not an anticipated goal at this phase. However, comparison of facility-based (clinics, hospitals) disease passive surveillance data before and after release and/or between areas where release has and has not occurred, advised previously as a safety precaution, might also provide some preliminary indication of epidemiological efficacy. It will be necessary to determine the value of putting additional effort into measuring health impact within these small-scale releases; sample size calculations based on prior knowledge of malaria prevalence and incidence at the trial location could help with these decisions. Further measurements that might be considered include incidence of cases (or severe disease or mortality), prevalence in different age groups, and seroconversion rate. Comparison with a historical or internal control may allow exclusion of other explanations for an observed change and suggest a causal relationship.^123^ Researchers are encouraged to consult with the WHO VCAG about their trial plans well in advance of releases.

### Site selection and containment requirements.

Many important factors should be taken into account when identifying the initial release site. These include existence of the necessary entomological conditions and adequate access to local, technical, and regulatory expertise, as well as appropriate isolation. Ideally, small-scale release sites also will be located distant from national borders. The working group recognized that it will be difficult to identify sites that provide all these factors and, therefore, some degree of compromise likely will be required.

The site for the initial small-scale release must at a minimum provide mosquitoes of the same species as the investigational gene drive product. Because *An. gambiae* feeds extensively on people, this will require the presence of humans. Because the primary goal of small-scale releases is to measure entomological efficacy, there is no requirement for any baseline malaria prevalence at the field site. However, the absence of local malaria transmission will influence how population replacement strategies are tested for entomological efficacy. Absence of malaria transmission at the initial site also will mean that any potential adverse effect on disease cannot be assessed until later releases.

The working group envisioned that these early releases could be small, perhaps only the size of a single village. Researchers are advised to plan for more than one release site (perhaps at least three) to increase flexibility and avoid unanticipated timeline delays. Whereas it may be ideal to have only *An. gambiae* at the first release site to maximize the ability to measure functionality of the gene drive construct, such sites are likely to be difficult to identify. Entomological efficacy can be distinguished in the presence of other malaria vectors. Moreover, it could be useful for a release to be undertaken at a site where other members of the *An. gambiae* complex in addition to *An. gambiae* s.s., particularly *An. coluzzii* and *An. arabiensis*, are present to assess the potential for spread of the driving transgenes to sibling species.

#### Ecological confinement.

There are differing opinions about the extent to which ecological confinement is achievable for *An. gambiae* mosquitoes in sub-Saharan Africa. Identification of ecological islands on the mainland where genetic isolation will be maintained reliably within a natural microhabitat is complicated by the general instability of species boundaries, although geographic barriers can contribute.^124^ Human mobility can facilitate the movement of mosquitoes, as the insects can involuntarily be transported in vehicles, boats, and airplanes. Evidence for other methods of long-range dispersal of *An. gambiae* also is building.^125^

Islands have important advantages over ecological containment on the mainland for initial testing of gene drive mosquitoes, however genetic analyses indicate that neither lake nor oceanic islands will provide absolute confinement or inability to spread beyond the island.^126–128^ Moreover, it must be remembered that conduct of field testing on islands also may introduce certain other challenges, including difficulty of access.

Because absolute isolation cannot be guaranteed at either mainland or island sites (see Table 3), the working group did not make any definitive recommendation regarding the location for initial release. Rather, it recommended that adequate thought be given early in the planning process to the degree of isolation achievable at the initial release site. In making this decision, researchers should consider not only location, human behavior, and mosquito behavior, but also possibilities for enhancing ecological confinement by creating buffer zones or taking advantage of seasonality.

**Table 3:** Some considerations for ecological confinement

Consideration	Island	Mainland
Efficacy	Possibility of less complex mosquito population genetics, less polymorphism, may increase chances of initial establishment	Easier to obtain conditions for realistic prediction of establishment and spread rate comparable to those necessary for testing epidemiological efficacy
	May provide a simpler mosquito population structure less generalizable to mainland settings	
	Lower potential for inward migration of wild-type mosquitoes may increase ability to detect establishment and spread	

Containment	Genetic evidence for some degree of isolation may simplify monitoring	Difficult to define boundaries and thus to control diffusion
	Boat or air traffic to and from islands may complicate detection of escapes	Any road or river traffic in the area could increase emigration potential

Monitoring	Depending on size, may facilitate intense monitoring	May require monitoring over larger area

Engagement	Malaria elimination may be achievable using conventional methods, decreasing willingness to try a novel strategy	Malaria elimination likely to require new tools, simplifying explanation of potential benefit for public health
	Depending on location, may be less likely to have local scientific leadership and champion for the technology	More likely to have research infrastructure, local scientific leadership, and champion

Regulatory issues	Regional approval desirable in the absence of guaranteed confinement	Regional approval highly relevant
	Depending on degree of isolation, may have special biodiversity considerations	

The initial introduction of gene drive mosquitoes should be undertaken in an environment that provides the necessary conditions for testing establishment, spread, and entomological efficacy while at the same time offering geographic isolation to maximize, to the extent practical, the possibility for ecological confinement.

Assuming no adverse effects are observed during initial introduction, the working group recommended that the need for geographic isolation could be lifted for subsequent small-scale releases of the same investigational product, although safety monitoring should continue as a matter of due diligence.

### Monitoring.

Monitoring for transgene spread in this phase will be simplified under conditions of strong geographic isolation, although as mentioned previously absolute confinement of gene drive mosquitoes cannot be guaranteed. There may be transport to and from islands that could provide opportunities for escape to other locations; for both lake and oceanic islands this will include boat traffic, and for larger islands may also include aircraft. This would introduce complexity in terms of where and how to monitor for escapees. A release at a mainland site may spread in a more predictable manner, enabling monitoring from the fringes of the release, but monitoring may need to cover a large geographic area. Any road or river traffic in a mainland release area could increase the possibility of unpredictable longer-range dispersal.

Monitoring for development of genetic resistance by mosquitoes or malaria parasites to the gene drive construct within local mosquitoes has been discussed previously (see *Efficacy*). This will require that laboratory and/or field assays have been developed and evaluated beforehand.

An important consideration at this phase of evaluation is the period for which monitoring must be conducted. Data will need to be archived during the monitoring period. As mentioned previously, it would be useful to continue to follow the release for as long as possible. Defining monitoring obligations will be particularly relevant if there is no intent to terminate the trial and clear the area, as previously has been the expectation for trials of GM crops. This decision would be simplified if the site becomes part of subsequent large-scale releases, as this would necessitate ongoing monitoring. Researchers and regulators should agree on a feasible monitoring plan that defines expectations, including duration, before releases. Funders must be prepared to support monitoring requirements imposed by regulators.

### Engagement.

The release area for small-scale trials should be sufficiently small to allow for intense community engagement through personal interactions. The engagement team should clearly describe the trial to the community members, with authentic explanation of risks and benefits, and seek their perspectives. Although this is best performed by project team members who are social scientists familiar with the local culture and are experts in engaging community members, there should be opportunities for the community to meet with project leadership, if desired. It is advisable to conduct a survey to judge the level of community awareness before asking for endorsement of a release. A community liaison or reference group could be helpful for providing feedback on the level of community satisfaction and whether the project is meeting its engagement goals.

Relevant ethics committees and/or regulatory authorities may need to approve the community authorization plan before release, which should be included in the project development timeline. Researchers should anticipate that ethics committees and/or regulatory authorities will require assurance that the community has access to the standard of care for malaria according to national policy.

Obligations may differ according to the experimental strategy, for example, if female mosquitoes will be released. Access to long-lasting insecticidal nets is required at this stage if not previously provided during baseline studies, because this represents the current best practice. Access to malaria medication is not usually a research project’s responsibility, but it is recommended that researchers work with the health care system to ensure that it is readily available. It should be noted that increased treatment access may initially result in increased reporting of cases, so, similarly to bed nets, it is best if these practices are established early to provide realistic baseline data. If personally identifiable data or specimens are collected as part of small field releases, researchers must observe the requirements of human subject research.

An important consideration is whether opportunities can be made available for individuals or households at the release site to choose to not participate and, if so, what these opportunities might comprise. For small-scale releases, options for responding to concerns within the hosting community may include: project agreement to avoid releasing in the immediate location of the residence, or if that is unsatisfactory, at some mutually agreed on distance from the household; provision of mosquito repellent and/or provision of traps to remove mosquitoes from the household. However, none of these options can guarantee complete lack of exposure to gene drive mosquitoes. The community reference group may have additional ideas in this regard and should be consulted early in project development. Researchers should remember that there will be a need for engagement around these options, to ensure that community members understand how to access and use them and what they offer in the context of gene drive.

It is possible that a malaria outbreak will occur naturally during testing or follow-up, for example, as a result of rains that support mosquito development. This possibility, along with anticipated malaria management strategies, should be discussed with the community in advance. Such an outbreak can be expected to trigger a need for intensive community engagement and broader public communication efforts. The risk to the project will relate to the level of understanding and trust that has been established within the community. Researchers must be prepared to work with the community and respond to its needs. For example, this may involve temporarily halting releases or ensuring malaria treatment is available in the area where malaria has appeared.

At this testing phase, engagement must have proceeded beyond the local community. Researchers should be engaging with local malaria and vector control programs, both to understand their plans for future vector control campaigns that might impact trial results and to begin sensitizing them about the technology and their potential role in its assessment. Discussions of the release plan through existing regional organizations before initiation of the trial would be wise. Also before releases, it will be important to have reached out to third parties who are likely to have influence to discuss the technology and the testing plans. This will include relevant policy makers, who must be kept informed of and involved in planning of all phases of field testing. Although it may not be possible to win the endorsement of all parties, it remains critical to continually interact broadly to enhance understanding and avoid misperceptions about the research.

### Regulatory issues.

Individual countries will be responsible for regulatory decision-making. As envisioned by this working group, if semi-field testing has been conducted then regulators will already have confronted the possibility of small-scale field release of gene drive mosquitoes, and authorization for both types of testing may have been granted. If not preceded by semi-field testing, or if requested by regulators, this small-scale field testing will require a new regulatory application. Although the NBA will be the first level of entry, it is desirable for health regulators also to play a prominent role at this phase.

A major difference between ecologically confined testing of gene drive mosquitoes and standard conditions for ecologically confined testing of GM crops is that, because *An. gambiae* feeds almost exclusively on people, humans likely will be exposed to gene drive mosquitoes at this early stage. This will require prior discussion with regulators to ensure understanding of the biological basis for this requirement.

Because regulatory experience with confined testing of GM crops assumes clearing the site following a trial, the concept of leaving the release site active also may require a change in regulatory paradigms. Biocontrol precedents would be helpful to provide context for this discussion. Researchers should begin discussions with regulators early in the planning of initial small-scale releases if they wish to make a case for leaving the release site active for long-term follow-up.

The extent to which regional regulatory acceptance will be necessary to allow initial small-scale releases of gene drive mosquitoes likely will depend on the level of confinement that can be achieved at the trial site and distance from international borders. Modeling may be helpful to inform this prediction. Researchers must be transparent with regulators about the potential for transboundary spread of the gene drive construct, based on all available data and information. Under current mechanisms, the decision to notify or consult with neighboring country regulators will be made by the regulatory authority of the country in which the trial is proposed.

### Key points: small-scale field-testing for entomological efficacy.

- Small-scale field trials primarily are intended to assess entomological efficacy under natural conditions, while still restricting environmental exposure to the extent possible. Although absolute ecological containment cannot be guaranteed for mosquitoes modified with low-threshold gene drive, initial small-scale field release should aim for geographic isolation, to minimize the possibility of outward migration.
- The primary goals of small-scale field releases are to 1) test the rate of transmission of the transgenic construct into the local *An. gambiae* population; and 2) continue assessing biological function (suppression or replacement). Resilience to development of genetic resistance also should be assessed to the extent possible given scale and timing. Moreover, these releases also must be designed to address any safety questions raised by regulators or other oversight bodies.
- Trials for entomological efficacy must be conducted where the malaria vector that is the subject of the investigational gene drive product is present. If malaria transmission is present at the site, data on malaria incidence and prevalence before and after release should be collected (e.g., through passive surveillance) as a safety precaution. Evidence for adverse impact on human health would constitute a “no-go” criterion that would end further testing and trigger remediation efforts.
- Multiple small releases at different sites likely will be needed to understand the effects of differences such as geography, climate, ecology, and human habitation on the investigational gene drive product, which will be important for planning a large-scale trial for epidemiological efficacy. Assuming no early evidence of adverse consequences, the expectation of geographic isolation can be lifted in subsequent small-scale trials of the same investigational product but observation for any unanticipated results related to safety should continue.
- The longer the observation of the release can proceed, the more information can be collected about the behavior of gene drive mosquitoes, spread of the transgenic construct, and ecological interactions. However, allowing the release to remain active may not be standard practice, especially in the case of regulatory systems that are used to contain testing of GM crops, which ordinarily requires termination of the trial followed by elimination of GM materials from the environment. If researchers desire to leave the release active at the site, they will need to make a strong case to regulators about the reasons for continuing to observe the release. This will be simplified if the site is integrated into subsequent large-scale trials. Researchers and regulators must agree on a plan for how long the release will be followed. This plan must define the point for reporting results and making decisions about moving forward to the next level of testing.
- Before releases, researchers and regulators must agree on a monitoring plan that is feasible and defines expectations. Researchers must be transparent about the potential for spread of the transgenic construct, based on the degree of geographic isolation and prior testing and modeling results. Funders must be prepared to support monitoring requirements imposed by regulators.
- Relevant ethics committees and/or regulatory authorities likely will need to approve the community engagement and authorization plan before release. Researchers should anticipate that requirement to ensure the community is provided with the standard of care for malaria according to national policy.

## FIELD-TESTING FOR EPIDEMIOLOGICAL EFFICACY

The ultimate measure of the efficacy of a gene drive intervention is its ability to reduce or eliminate morbidity and mortality due to malaria parasites, without significant long-term costs to the socioeconomic system in which it is deployed. How epidemiological efficacy can be evaluated may differ depending on whether researchers are working in a malaria control setting (where there is still appreciable malaria transmission) or a malaria elimination setting (where disease burden and transmission levels will be low).^129^ It will be important to anticipate what the malaria situation will be at field sites at the time the investigational gene drive product is ready for field-testing, as more countries move toward elimination.

Researchers should strive to keep the trial design simple, as experience teaches that unexpected complications often arise during trial conduct. Entomologists are advised to enlist the participation of those with expertise in design and conduct of clinical trials, including epidemiologists and statisticians, to support development and execution of epidemiological efficacy trials. World Health Organization has provided recommendations for design of epidemiological efficacy trials for vector control products.^130^ Before entering into testing for epidemiological efficacy, researchers should understand as much as possible about the behavior of the investigational gene drive product from prior releases, and in particular how it responds to seasonality. Researchers should think carefully about the level of malaria transmission for which their investigational product would be best suited.

As explained in the WHO Guidance Framework, at the end of the epidemiological efficacy testing phase sufficient data should have been collected to understand spatial dispersal, transgene activity, ecological interactions, and the effect on disease transmission,^15^ as these will be key factors in the decision to implement the technology more broadly. Some sense of the cost of deployment also may be available, but this must take into account that delivery methods likely will not yet be optimized.

### Safety.

It will be necessary to update the all-hazards risk assessment before moving to large-scale releases, considering conditions specific to the trial design and location.

Trials for disease impact will involve human subject research and, therefore, it is expected that the project will be registered as a clinical trial in a public registry (such as the existing International Clinical Trials Registry Platform^131^ or U.S. National Library of Medicine ClinicalTrials.gov^132^). It is standard practice in clinical trials to monitor for adverse health events, and to determine whether these occur differentially in study arms and the likelihood that they are related to the intervention. A trial steering committee and DSMB must be put in place to regularly review adverse event reports and provide independent oversight of participant safety as well as efficacy of the investigational product.^133^

Ecologists should be incorporated in the team at this stage. Although no adverse environmental effects would be anticipated at this phase given prior safety studies and environmental monitoring, ongoing routine surveillance is still necessary for due diligence. It is expected that this will focus on case-specific monitoring based on risk assessment. Continued observation of mosquito community structure is a logical possibility. It also may be advisable to identify a manageable number of nontarget organisms with a predicted high exposure to the transgenic mosquitoes for surveillance during large-scale trials (for example, representative first order organisms as defined in Table 2). Although trials for epidemiological efficacy may be centered on villages, it is advisable to conduct monitoring in areas away from villages, as well as within them, to allow for the possibility of detecting ecological impacts that are different from those near human-dominated settings. Evidence for absence of nontarget effects at this phase might reduce concerns about the need for general surveillance during the post-implementation monitoring period.

### Efficacy.

There are several ways to collect evidence of epidemiological efficacy in a setting where there is a significant malaria disease burden. If the ability of gene drive mosquitoes to prevent malaria transmission is as significant as predicted by some modeling, efficacy against malaria may become obvious even during small-scale releases. However, because experience suggests that the performance of new vector control tools in the field is rarely at the level predicted from laboratory studies and modeling results, the working group assumed a need for generating high-quality evidence of epidemiological efficacy by randomized trials. This likely will require larger field sites, which introduces additional complexity for planning and execution. It is quite likely that these sites will contain several species of malaria vector. In that case, the investigational gene drive product ideally would target all major vectors of the *An. gambiae* complex in the area. If the product targets only a single species, it will be especially important in designing the trial to rely on modeling to predict its potential effect in the presence of other vectors, as this will affect the establishment of epidemiological endpoints. In this regard, it will be necessary to understand the proportion of malaria transmission attributed to the specific vector species being targeted as part of baseline data collection.

One possible approach is a stepped-wedge design, with sequential roll-out across sites in a randomized entry order.^134^ This approach could build on sites of earlier small-scale release. The cluster randomized controlled trial (CRT), in which each cluster is a village or group of villages, is considered the gold standard for assessing epidemiological efficacy of vector control products.^15,130,135–137^ In this design, clusters will be randomly assigned to either receive releases of gene drive mosquitoes or to remain untreated, serving as controls. It is critical to ensure that all other factors are as similar as possible between control and intervention clusters, including the use of other vector control methods. To achieve this, study arms should be balanced using restricted randomization. Randomization should be conducted in a public, transparent manner involving communities wherever possible. Researchers are strongly advised to coordinate the timing of the trial with delivery of routine vector control interventions by the national malaria control program. The bigger the treatment effect, the fewer clusters that will be required to observe a significant difference.

The characteristics of gene drive technology introduce complexities for any trial design. The most obvious of these is the potential for driving transgenes to spread to mosquito populations in control areas before the end of the trial, which requires predicting how isolated from each other the intervention and control clusters must be. Presence of buffer zones may be helpful to prevent, or at least slow, such spread^138^; their optimal size and other features should be informed by data obtained from small-scale releases. A related concern is predicting how quickly each of the intervention clusters will “convert” (that is, most, if not all, of the target mosquitoes within the cluster carry the gene drive construct) after release of gene drive mosquitoes, and thus when to begin measuring for the gene drive effect on malaria. If measurements begin before the gene drive mosquitoes have spread throughout the intervention clusters, this could result in a lower than realistic effect. Depending on cluster size, it may be possible in per protocol analysis to measure the correlation of change in transmission parameters and/or malaria incidence along the leading edge as the driving transgenes spread through the cluster—although the usual patchy distribution of mosquito populations may complicate this type of analysis. Trial planning for gene drive interventions will require substantial forethought and an adaptive trial design may be necessary. Data collected from small-scale trials, such as the rate of spread of the transgenic construct, will aid the planning for epidemiological efficacy trials, which underlines the need for early testing to be conducted under conditions as similar to the environment of trials for epidemiological efficacy as possible.

In a setting with appreciable malaria transmission, the primary clinical endpoint will be the one used for assessing other malaria interventions—a reduction in incidence of infection in children measured by cross-sectional surveys, or in disease measured by active and/or passive case detection.^130^ When setting the efficacy goal, researchers must keep in mind the potential for malaria transmission by vectors that are not the focus of the gene drive strategy. The goal in terms of level of reduction in malaria incidence/prevalence will have to be determined site by site because of differences in malaria transmission conditions and standard mosquito control practices. Double blinding, a widely accepted method for preventing bias by ensuring that neither the trial participants nor the researchers know who is receiving a particular treatment, could be difficult for so obvious a treatment as release of transgenic mosquitoes, and the safety and ethical implications of releasing similar numbers of unmodified mosquitoes in control clusters would need to be explored thoroughly. However, at a minimum those technicians who are assessing malaria incidence from blood specimens should be kept unaware of the source of samples and the identity of treatment versus control clusters.

Entomological endpoints will be determined according to the gene drive strategy as in earlier small-scale releases. Primary entomological endpoints could be sporozoite rate (for population replacement strategies) and mean number of *An. gambiae* per house per night (for population suppression strategies). The required sample size should be calculated based on the primary clinical endpoint, but the entomological conditions must also be taken into consideration in sample size calculation to better understand how gene drive affects the vector population.

Secondary endpoints might be:

- Clinical, such as anemia and parasite diversity (especially for population replacement strategies).
- Entomological, such as, indoor and outdoor biting patterns through the night, resistance to gene drive, or changes in insecticide resistance. Newer methods, such as detection of antibodies to mosquito saliva,^139^ also may provide information on the number of bites to which individuals are exposed.

The WHO recently expressed a preference to see results from at least two CRTs to make a decision on recommendation of new vector control tools. The preference would be for these to be performed in different areas (such as East and West Africa) and different settings (high and low malaria transmission).^32^ However, the working group acknowledged that a CRT design might not be feasible for a rapidly spreading gene drive construct. In this case, the realistic alternative would be simply to track the spread of the transgenic construct and to correlate it with a changing incidence of malaria cases. Such comparisons are complicated by the likelihood of various confounders, but there is increasing experience with this for vaccine studies. If the reduction in malaria transmission is sufficiently significant, regulators may accept the gene drive product without more rigorous trials. In that case, they may impose some additional efficacy monitoring requirements after wide scale deployment as a public health tool (similar to Phase 4 in drug trials).

As mentioned previously, it is possible that by the time a gene drive tool is ready for large-scale testing the malaria burden may have been dramatically reduced by diligent application of other control methods. In an elimination setting, efficacy measures other than reduction in incidence of malaria cases in children may need to be explored. This might involve measurement of a reduction in the incidence of malaria infections in all age groups, as detected by a sufficiently sensitive method. This will require large populations to ensure a sufficient accrual of cases. In settings of very low transmission, routine surveillance systems are usually of higher quality and these may be used for passive case surveillance instead of setting up cohorts. Moreover, the value of the technology may be in its durability and capacity to reduce receptivity to reintroduction of malaria at sites that have been cleared (i.e., as a “last mile” technology for achieving malaria eradication).

Alternative options are possible for measuring the efficacy of gene drive mosquitoes, and trial design must consider the characteristics of the investigational product and the malaria transmission setting under which it is tested. Flexibility may be required to adjust expectations according to conditions at the time of testing. Researchers should consult in advance with the WHO VCAG in the development of their testing plans to ensure the results will contribute meaningfully to WHO decision-making about product utility as a public health tool.

### Site selection.

Baseline data from the site over at least 2 years before the trial should be available. It will be critical that baseline studies at the site of epidemiological efficacy trials identify all malaria vector species present, and their relative densities and infection rates, as this will influence planning for detection of reduced malaria transmission. In the lead-up to this phase, up-to-date site-specific entomological surveillance, linked to modeling, should be used to translate from the extant mix of species in a location to the epidemiological impact that can be expected for a construct of a given efficacy, which relates back to identification of the relevant clinical endpoints.

For efficiency, it would be ideal to use the same infrastructure and personnel as in earlier testing efforts. Indeed, as previously mentioned, one scenario is that the epidemiological efficacy trial will simply grow and expand from the prior small-scale testing site(s). This should be taken into consideration when choosing the sites for small-scale field testing. For a CRT, the site will need to provide sufficient numbers of clusters with sufficient separation to minimize spillover of gene drive mosquitoes into control areas before the end of the trial as described previously.

From a regulatory and a stakeholder engagement perspective, it would be desirable to locate the trial as far as possible from a national border or major transport routes to other countries. Alternatively and ideally, because the eventual spread of the gene drive mosquitoes is likely, neighboring countries might collaborate on this research. The extent of regional coordination, including regulatory harmonization, will be a factor in the decision as to how important distance from national borders is for site selection.

### Monitoring.

It will be important for researchers, funders, and regulatory authorities to agree on a feasible monitoring plan well in advance of the epidemiological efficacy trial, which describes expectations for the extent, that is, frequency, scale and period, of monitoring. Monitoring for spread of the transgenic construct during large-scale releases is likely to require an extensive network for trapping and collection, along with facilities and resources for processing and analyzing samples. As in small-scale releases, it will be important to follow the gene drive construct by all available means. Monitoring for development of genetic resistance to the gene drive construct within local mosquitoes must be conducted. For population replacement strategies, monitoring for appearance of malaria parasites resistant to the effector mechanism is also required. Ideally, national malaria control/elimination surveillance and monitoring teams will become involved in these efforts. This would help to build technical awareness and capacity in anticipation of their future role in implementation and post-implementation activities.

### Engagement.

Epidemiological efficacy testing necessarily will involve interactions with human participants living in the trial area for the purpose of collecting individually identifiable information and/or specimens, and this must be conducted according to standards for human subjects research. As described previously, however, not all individuals living in the trial area will meet these criteria. Thus, broad community engagement is a vital aspect of preparation for and conduct of this phase of testing. At this larger scale, there may be less opportunity for a personal approach to community engagement and endorsement. More emphasis may need to be placed on wide distribution of informational materials, interactions with key opinion leaders and influencers, and on mechanisms such as reference or liaison groups to gauge community opinion. Given the area that may be covered in epidemiological efficacy trials, consideration might be given to the suitability of an “opt-out” model of consent. The engagement plan should provide for ongoing communication with the community about the trial’s progress. Media interactions can help in this regard.

It must also be recognized that there is a higher probability of dissent at the scale of an epidemiological efficacy trial. Serious consideration must be given to providing meaningful opportunities for individuals or households to decline participation. Whereas some serious options can be presented, such as not allowing releases or monitoring at their home or place of work and/or not participating as human subjects through provision of personal identifying information or specimens, it likely will be increasingly difficult to prevent some degree of exposure to gene drive mosquitoes as large-scale testing proceeds. The extent of exposure is likely to differ according to whether a suppression or replacement strategy is being tested. Expectations for the long-term spread of the gene drive construct within the local mosquito population must be conveyed realistically in the engagement process. It is advised that a survey of public understanding of these expectations be undertaken before releases. Some mechanism for protecting the privacy of trial participants may need to be considered, perhaps similar to the standardly used certificate of confidentiality (for example, certificates of confidentiality from the U.S. National Institutes of Health^140^).

Government-level championship of the research will be essential before this stage, as this will be critical for regional interactions. In addition to the government of the host country, researchers must consider neighboring countries as stakeholders and begin interacting regionally before the trial begins. For example, presentations about the project might be made at regional meetings of health ministers and national malaria control programs. Results from small-scale trials on the rate at which the transgenic construct will spread in the local *An. gambiae* population will help to inform these interactions.

Regional and international organizations concerned with malaria control must be engaged before large-scale releases begin, although preferably this will have occurred even earlier in the project. As described for small-scale releases, researchers must ensure access to appropriate standard of care for all households involved in the trial. This likely will require coordination with the national malaria control program in trial planning.

### Regulatory issues.

Regional scientific collaborations and a mechanism for regional regulatory authorization are especially important for large-scale releases, and a framework will need to be put in place for this. It is anticipated that the national regulatory authority for the country hosting the trial will be responsible for informing other countries’ regulatory bodies and invoking any regional cooperation mechanisms, although it is possible this process might be initiated by a regional body depending on the status of regional harmonization activities.

The possibility exists that more than one project may be advancing its investigational gene drive product toward field-testing within the same time frame and within the same (or nearby) regions. This could be challenging for risk assessment, as well as efficacy and safety assessment and stakeholder engagement, if the different gene drive products were to spread and overlap. If multiple trials are proposed within the same country, the national regulatory authority would be aware of the applications and should be responsible for determining whether a need exists to invoke additional management requirements. This situation could become very challenging to manage if the different trials are being regulated by different countries, underscoring the importance of a regional coordination and authorization process. Information on final decisions about importation or release of living modified organisms is required to be provided to the Biosafety Clearing-House of the Cartagena Protocol,^141^ and, as mentioned previously, there are existing websites for registering clinical trials. However, a centrally managed trial registration/declaration website could make information on gene drive mosquitoes easier to access.

The WHO will intervene at the request of a country. Prior interaction with the WHO will be important in case it is called on by a country to become involved in trial issues. Researchers are advised to consult with the WHO VCAG during trial planning, which can advise on study design and protocols. The best approach is to ensure that the host country has been thoroughly involved in planning the CRT at all levels (local Ministry of Health or equivalent, malaria control program, local institutions, and WHO local office).

### Key points: field testing for epidemiological efficacy.

- The goals of large-scale testing for epidemiological efficacy will be to determine the effect on disease transmission, and to understand spatial dispersal and ecological interactions, as these will be key factors in the decision to incorporate the gene drive product into national malaria control programs. Optimally, epidemiological efficacy testing would be conducted under at least two different malaria transmission settings (high and low). Before the trial, it will be critical to have identified all malaria vector species present at the trial site(s), their relative densities and infection rates, as this will influence the ability to detect reduced malaria transmission. When setting the efficacy goal, researchers must keep in mind the potential for malaria transmission by vectors that are not the focus of the gene drive strategy.
- For gene drive mosquitoes, if prior small-scale releases are allowed to remain active, large-scale trials may build on and expand these. The widely preferred design for determining efficacy of vector control tools is the CRT. However, although the CRT works well in malaria control settings, it must be recognized that by the time investigational gene drive products reach this stage of testing, some parts of sub-Saharan Africa are likely to be close to malaria elimination. Moreover, specific characteristics of the investigational product, particularly the rate of spread of the transgenic construct, likely will require flexibility and innovative trial design.
- The goal in terms of level of reduction in malaria incidence and/or prevalence will have to be determined site by site because of differences in malaria transmission conditions. In a setting with appreciable malaria transmission, the primary clinical endpoint will be that used for assessing other malaria interventions—a reduction in malaria incidence in children measured by active and/or passive case detection. In a malaria elimination setting, efficacy might better be measured by reduction in prevalence of infection. Durability of the effect (capacity to reduce receptivity to reintroduction of malaria) also may be an important efficacy indicator.
- As for earlier small-scale releases, it will be important for researchers, funders, and regulatory authorities to agree well in advance of the trial on a feasible monitoring plan that describes expectations for the extent, that is, scale and period, of monitoring. At this point no adverse environmental effects would be anticipated given prior safety studies, but hypothesis-based monitoring is still advisable as a precaution. The monitoring plan should be derived from results of risk assessment.
- Testing for epidemiological efficacy will involve collection of identifying information and specimens from individuals living at the trial site, which will require individual informed consent. Wider community engagement activities also will continue to be necessary to involve those living in the area but not directly participating as human subjects. Researchers will be expected to register in a clinical trial database at this phase of testing.
- Broad stakeholder and public engagement will be required before the initiation of large-scale trials. Researchers must consider neighboring countries as stakeholders and interact regularly with them, sharing information about the investigational product and results from earlier trials. This will be facilitated if a regional approach is taken at the stages of semi-field testing and small-scale releases.
- The potential for transboundary movement will be especially important at this phase, and a regional coordination and authorization process will be essential to prepare for large-scale releases of gene drive mosquitoes.

## IMPLEMENTATION AS A PUBLIC HEALTH TOOL

Because of the characteristics of potential persistence and spread, implementation of gene drive mosquitoes as a public health tool within national malaria control programs likely will build onto prior large-scale releases for testing epidemiological efficacy, moving to broader and more systematic regional distributions. Observations from prior releases of the behavior of the investigational product under different geographic and ecological conditions, in combination with modeling, will inform the design of deployment plans (see Box 8).

Box 8 **Release strategy**

Design of a release strategy to achieve the goals of malaria control or elimination must take into account the characteristics of the specific gene drive mosquito product and factors such as the local population size of the target mosquito species, mosquito movement and spatial connectivity between populations, and the effects of seasonality. The release strategy as implemented may involve hundreds to thousands of mosquitoes released over a limited period of time from each of a potentially large number of spatially distributed sites. Despite the prediction that establishment may result from accidental release of low numbers of gene drive mosquitoes, higher numbers likely will be needed to maximize the chances of implementation success.

### Decision-making.

The decision to implement at scale is expected to be based on proof of efficacy, safety, and other measures of acceptability, and perception of need (benefit) and overall cost (including deployment and follow-up activities).^15,142^ For gene drive mosquitoes, these considerations will be viewed in the context of malaria elimination and eradication goals, which aim to maintain the burden of malaria stably at zero forever. This will require an overall strategy that is robust to any reversal that might result from, for example, evolution of resistance mechanisms that reduce the efficacy of individual malaria control tools, decreases in funding that impede accessibility of those tools, or failure of delivery systems due to economic/social crisis or user apathy. Gene drive mosquitoes may prove highly effective at reducing malaria transmission on their own,^14^ and thus be a critical control tool for use in conflict areas or under other conditions where conventional tools cannot be delivered effectively. However, the working group recommended that they generally should be considered, at least at first, as complementary to other malaria control measures within an integrated strategy that will provide the best protection against malaria resurgence because of any of the aforementioned conditions. In this case, the benefit and cost of implementing gene drive mosquitoes will be considered based on additive value in combination with other interventions. Prior assessment of the utility of combinations of gene drive mosquitoes with other commonly used vector control interventions, either in the context of field trials or by modeling, will be important to inform decision-making.

The WHO has indicated that scale-back of vector control may be considered in areas where malaria transmission has been interrupted and reduced vulnerability and receptivity indicate minimal risk of resurgence, given the presence of adequate disease and entomological surveillance systems along with capacity for case management and vector control response.^143^ Gene drive mosquitoes are expected to reduce transmission intensity, which should lower the required level of coverage with other interventions and contribute to cost savings. Moreover, they are predicted to be useful for preventing reintroduction of infection in areas where malaria has been eliminated, which could contribute to the durability of cost savings and be crucial in the last mile of an eradication campaign.^144^ For population suppression strategies, an additional benefit may come from control of other diseases carried by the same vector; for example, in the case of *An. gambiae* s.s., this would include lymphatic filariasis.

A prerequisite for implementation of gene drive mosquitoes will be long-term prior engagement with government authorities, especially malaria control or elimination programs, underpinning their ability to make a decision about the value proposition for the technology and the role they wish to take. This engagement could build on the regional mechanism recommended for cooperation and coordination around large-scale field trials.

### Production and distribution.

To ensure a smooth transition to implementation, researchers, funders, regulators, and government policy makers must begin thinking early on about how gene drive mosquitoes will be produced and distributed, as this may influence the development pathway. Registration is generally the last step of the regulatory approval process. It sets the marketing conditions for product quality, labeling and effectiveness claims, usage conditions, and, if applicable, advertising and promotional materials, and requirements for post-approval monitoring, adverse event reporting, product experience reporting, and manufacturing changes. For commercial products, the applicant or registrant (normally the developer or manufacturer) is required to assure ongoing product quality and to comply with other conditions of approval. There remains a need to determine the most appropriate business model for implementation of this new technology, which will best facilitate taking the product to scale in target countries.^145^ Current thinking on gene drive mosquitoes is to transfer the technology to national governments for use in their national malaria control programs.

Manufacturing is expected to require relatively small-scale insectaries, which might be nationally or regionally distributed, or even mobile, and could be operated by the government. The mechanisms and requirements for registration of gene drive mosquitoes are unclear at this time and require further consideration because this will influence planning around issues of intellectual property, liability, and post-deployment monitoring. Registration is usually performed on a national basis, but in the case of gene drive mosquitoes regional harmonization of registration requirements and processes may be required. Understanding how such challenges have been met for agricultural biocontrol agents could provide useful insights.^146^ Regulatory harmonization efforts under the African Medicines Regulatory Harmonization program may also provide a model.^147^

If gene drive mosquitoes are to be produced and distributed by government programs as a component of integrated vector management (IVM), countries must be ready to take responsibility for these aspects of their deployment, along with the necessary public engagement and post-implementation monitoring.^148^ This should be facilitated by the recent commitment of African leadership to eliminate malaria by 2030.^148^

Keeping in mind the potential for, and implications of, transboundary movement, decision-making about approval for implementation will need to take place on a regional or even continent-wide basis and a framework will need to be put in place to coordinate and support deployment. A relevant model is the Pan-African Tsetse and Trypanosomiasis Eradication Campaign,^150^ which was agreed on by African Heads of State, is managed through a coordination office that was established by the Organization of African Unity’s Secretary General, and is run by national authorities.

### Policy development.

Researchers developing gene drive mosquitoes for use in developing countries must be aware of the influential evaluation and prequalification process conducted by WHO. Prequalification follows assessment, recommendation and policy development for new tools and approaches, and is intended for products that already have a place in disease control operational strategies.

Countries likely will look to WHO for guidance on the utility of gene drive mosquitoes as a public health tool. Within the WHO process for evaluation of vector control products, gene drive mosquitoes will, at least initially, be considered as a new product class and, therefore, will be reviewed through the New Intervention Pathway.^151^ Currently, under this process, the WHO will develop evaluation standards and the WHO VCAG will review supporting data submitted by the applicant (researchers or other developers) on entomological and epidemiological efficacy, and potential public health impact, of the gene drive mosquito product. The WHO VCAG recommendation on the public health value of the product will be forwarded to the relevant policy advisory committee, which in the case of malaria is the Malaria Policy Advisory Committee. On the basis of the strength of the evidence, the WHO may issue a policy recommendation and develop operational guidance for its use in malaria control programs. The WHO prequalification team will coordinate any needed dossier review, finalization of quality control criteria and inspections of product manufacturing facilities with the VCAG process, so that once policy guidance has been issued, a decision can rapidly be made on prequalification of a product. In the future, depending on the nature of the technical strategy used for each new product, gene drive mosquitoes may become recognized as a product class for which a policy recommendation has been issued. If so, they would enter directly into the prequalification pathway. In such a case, some of the extensive risk assessment requirements recommended previously for “first-in-class” products may not be considered necessary.

### Quality control.

Currently there are no widely recognized quality standards for manufacture of a living mosquito product. Most relevant programs that are now in field trials, including those based on sterile insect technique or other strategies, still are operating in investigational rather than commercial mode. As such, developers are defining individual standards for quality and consistency of their products, which are subject to regulatory approval. Although at the time of this writing, none of these efforts involve *An. gambiae* mosquitoes, lessons learned from large-scale production and maintenance of mosquito colonies within these programs, or from similar efforts for other insect vectors of disease, could be valuable for establishing broader standards in the future.^152^ Ideally, this would include the identification of simple and cost-effective methods to predict performance in the field.

A particular issue for *An. gambiae* products will be the current inability to adequately cryopreserve seed stock of transgenic mosquitoes, which results in a need for transgenic lines to be constantly maintained through all life cycle stages in the insectary. Unless this challenge can be overcome by discovery of improved preservation techniques, regulators will need to understand the implications of this requirement for ongoing maintenance on the genetic lineage with reference to product quality and product specifications. Avoidance of adaptation to insectary environments, which could result in reduced fitness, may require occasional refreshing of the colony through crossing with local mosquitoes. Moreover, there may be a need to adapt the gene drive product to different locations to improve the probability of initial establishment, which also would be accomplished by introgression of the genetic construct into the genetic background of the local mosquitoes.

Regulatory authorities may not consider such breeding activities as a new transgenic event, in which case a new risk assessment would not be needed. However, the usual regulatory considerations of identity, strength, quality, purity, and potency applied to other types of products almost certainly will have to be reinterpreted for gene drive mosquitoes. Thus, there is a need to develop quality standards for manufacturing of living mosquito products that are consistent with their particular production requirements.

### Key points: implementation as a public health tool.

- Because of the characteristics of persistence and spread, it is likely that implementation of gene drive mosquitoes as a public health tool within national malaria control programs will build onto prior large-scale releases, moving to broader and more systematic regional deployment that will be based on observations of the product’s behavior under different geographic and ecological conditions.
- Gene drive mosquitoes should be considered as part of an IVM plan to control and eliminate malaria. The decision to implement is expected to be based on proof of efficacy, safety, and other measures of acceptability, as well as perception of need (benefit) and overall cost (including deployment and follow-up activities). These considerations will be viewed in the context of national and regional malaria elimination and eradication goals.
- Because of the implications of transboundary movement, decision-making about approval for implementation will need to take place on a regional or even continent-wide basis, and a framework will be required to support this. The WHO will play an important role in evaluating the product and making a recommendation to potential user countries about its value as a public health tool for malaria control.
- Researchers, funders, regulators, and policy makers must begin thinking early about how gene drive mosquitoes will be produced and distributed, as this may influence the development pathway. Current thinking on gene drive mosquitoes is that the technology will be transferred to national governments for use in their national malaria control programs, but there remains a need to identify the most appropriate business model to facilitate this. Long-term engagement will be required to ensure government authorities are familiar with the technology and have the information they need to judge the value proposition of taking on this role.
- Most current trials of living mosquito products, including those based on sterile insect technique or other strategies, are operating under research authorization and defining their own standards for product quality and consistency, which are subject to regulatory approval. For scaled-up deployment as a public health tool, there is a need to develop quality standards for manufacturing of living mosquito products that are consistent with their production requirements.

## POST-IMPLEMENTATION SURVEILLANCE, MONITORING, AND EVALUATION

After a decision is made to deploy gene drive mosquitoes as a public health tool, there will be a need for ongoing surveillance, monitoring, and evaluation. In this regard, the working group re-emphasized the following three important points:

1. The requirements for post-implementation monitoring and evaluation of gene drive mosquitoes must be considered on the background of current activities routinely conducted by national programs to monitor malaria cases and the efficacy of vector and other control methods, as well as activities that must be put in place in the context of malaria elimination efforts. Malaria surveillance is identified as a core intervention in the Global Technical Strategy for Malaria 2016–2030.^31^
2. The loss of efficacy due to genetic selection is a general challenge for malaria control tools, including insecticides, drugs, and diagnostics.^28,153^
3. Reduction in the number of malaria vectors is a generally accepted public health goal and the aim of all existing insecticide-based control methods. Commonly used insecticides are known to have effects on a broad range of nontarget species,^154^ and gene drive mosquitoes must be considered in the context of their relative risk and benefit versus other forms of vector control.

### Current monitoring considerations.

Monitoring of vector control methods currently includes operational aspects (such as coverage, usage, quality, and durability), entomological surveillance (including local vector species composition and distribution, and susceptibility to insecticides, vector, and human behaviors), and epidemiological surveillance (such as malaria incidence, malaria prevalence, and mortality). Standard indicators for vector surveillance include larval density and abundance, adult abundance, degree of vector contact with different hosts, the infection rate of the vector, and vector susceptibility to the control method of interest. Choice of which indicators to include will be influenced by whether the gene drive strategy aims at population suppression (which will emphasize monitoring of vector numbers) or replacement (which will emphasize monitoring susceptibility of the vector to the pathogen). Examples of standard entomological monitoring assessments are shown in Table 4, although it is unusual for any program to conduct the entire range of tests.

**Table 4:** Examples of standard monitoring considerations for current vector control methods

Vector control methods	Operational monitoring	Entomological monitoring
Long lasting insecticidal nets	Dosage	Biting cycle in relation to sleep habits
	Coverage	Human blood Index
	Timing	Human biting rate
	Persistence	Sporozoite rate
	Status of equipment	Adult density
	Resources used	Insecticide susceptibility status
	Cost	

Indoor residual spray	Dosage	Daytime indoor resting
	Coverage	Human-biting rate
	Timing	Human blood Index
	Persistence	Parous rate
	Status of equipment	Sporozoite rate
	Resources used	Insecticide susceptibility status
	Cost	Adult density

Larviciding	Coverage	Presence and density of larvae
	Persistence	Adult mosquito density
	Resources used	Insecticide susceptibility status
	Cost	

Source reduction	Number of potential breeding sites	Adult mosquito density
	Number eliminated	
	Resources used and cost	

Improved housing	House design and structure	Indoor mosquito resting densities
	Mosquito proofing	
	Distance from potential breeding sites	
	Resources used and cost	

Requirements for epidemiological surveillance will differ under different transmission conditions. In malaria control settings, where the disease burden remains high, surveillance, monitoring, and evaluation generally includes a combination of passive surveillance of cases reported through the health system and periodic representative household surveys to assess population coverage of key interventions. In low-transmission or elimination settings,^155^ surveillance mechanisms must be able to detect more dispersed malaria transmission foci. In such situations, all cases need to be investigated and managed in a timely manner to prevent onward transmission of disease. This may be accomplished through active case detection, whereby individuals are tested whether they are symptomatic or not. This is termed reactive case detection when it is conducted in response to a case presenting at a health facility, and usually involves testing of individuals living in the same or neighboring households of the index case. Foci investigation, wherein an area of transmission or transmission potential is defined through active case detection, geographical reconnaissance, entomological investigation, and community behavioral surveys, will be a key component of surveillance for malaria elimination programs and will identify areas that need to be closely monitored.

Regardless of whether the goals of malaria elimination and eventual eradication are to be reached solely with more traditional drug, vaccine, and vector control tools or will incorporate the use of gene drive mosquito products, significant investment of resources will be required for training and facilities and improvements in information collection, data management, and reporting. Thus, the working group recommendations focus on different or additional requirements especially relevant to the use of gene drive mosquitoes. These largely relate to ongoing monitoring for lack of spread of the gene drive construct into the local vector population, any loss of efficacy, or adverse effects on human health or the environment.

### Safety.

The working group looked for relevant precedents from agricultural biocontrol and GMO as background for formulating its recommendations on gene drive mosquitoes. For biocontrol agents, regulators decide to allow releases based on risk analysis and data collected in containment. Generally, there is no formal process of post-release monitoring and evaluation or for reporting back to the regulator. Thus, there is no mandated time frame or area for follow-up and this is left to the discretion of the developer, who will be influenced by funding constraints. Post-release monitoring may focus more on efficacy issues such as how well the biocontrol agent established, how far it spread, and whether it had the desired effect on the target species.^156^

Post-release requirements for agricultural GMO differ widely among countries. Monitoring may involve general surveillance for unanticipated effects and/or case-specific surveillance for identified nonnegligible risks. Currently, Brazilian law does not require post-release monitoring of GMO. However, the National Biosafety Technical Commission has adopted a general surveillance approach that requires the applicant to make information about the release available to the public, provide questionnaires to users of the GMO technology and others involved in the process to facilitate reporting, and may require other notification systems and monitoring tools in line with the GMO use application; case-specific monitoring would be triggered if nonnegligible risks are identified. In the United States, where GMO are reviewed by different federal regulatory authorities according to their product claim and intended use,^157^ requirements differ among the different regulatory bodies but may include a requirement for the applicant to report adverse effects and monitor for any changes in safety or effectiveness or failure to meet the approved product claim including the occurrence of resistance. In Australia, the applicant is required to report on any adverse impacts, unintended effects or other information relating to potential harms to human health and safety or the environment. In the European Union, applicants are required to submit a plan for monitoring that may include both case-specific monitoring (e.g., for development of resistance) and general surveillance (e.g., through surveys of those conducting related work or existing monitoring networks and literature searches of relevant studies). In Africa, several countries have experience with GM crops, but few have established procedures for post-release monitoring. South Africa, which has extensive experience of commercialized GM crops, has developed a post-marketing monitoring strategy that takes into account monitoring for compliance with the release permit and independent monitoring for unintended effects on biodiversity.^158^

Thus, currently, there is no uniform precedent from either biocontrol or GM crops on which to base post-implementation monitoring recommendations for gene drive mosquitoes. By the time an implementation decision is made, individual gene drive mosquito products will have undergone extensive risk assessment that should have taken into account all identified hazards to human health and the environment, and possible pathways to harm. Such products also will have undergone rigorous study during small- and large-scale trials, including intensive observations on health and ecosystem effects. It should be presumed that the technology would not have reached this stage if there was any indication of nonnegligible adverse effects that could not be suitably mitigated, or if authorities had not determined that the benefits outweighed any adverse effects. If prior studies have been conducted over a range of ecological conditions, they may provide sufficient data and experience to justify an exemption from ongoing surveillance for broad ecosystem effects. However, because at present gene drive mosquitoes are a novel technology, it is likely that some degree of ongoing post-implementation monitoring of effects on carefully specified nontarget organisms will be expected. Effects on human health will be addressed through ongoing monitoring for epidemiological efficacy (see *Efficacy*), which will determine whether malaria incidence decreases or increases after implementation.

If continued ecosystem monitoring is required by regulators, it will be important for parties to agree beforehand on what is to be monitored and to what extent, as well as where responsibility for response would lie should an adverse event be detected. This might include agreement on conditions under which monitoring could be stopped if no adverse effects are observed. Those who make this decision will need to keep plausibility and feasibility well in mind, given the envisioned use conditions. Gene drive mosquitoes are not expected to be a commercial product where profits might offset monitoring costs, but to be delivered by governments as a component of IVM. Moreover, gene drive mosquitoes most likely will be implemented in conjunction with commonly used insecticide-based vector control methods that could have an independent effect on the ecosystem. And finally, the complex relationship between mosquitoes, climate conditions, and human behavior likely will make it particularly difficult to attribute any observed ecosystem changes directly to exposure to the gene drive product.

Regarding case-specific monitoring, the working group felt that it will be important to keep track of changes in the local mosquito community structure. The focus of these recommendations, a gene drive product in *An. gambiae* s.s., presents an unusual case, in which it would be considered desirable for the genetic construct to move through cross-mating to other malaria vectors within the *An. gambiae* s.l. species complex. It should be made clear that movement of the construct to sibling species that also are malaria vectors is considered part of the intended effect, and in that case functionality with respect to population suppression or replacement should be monitored in those other species. It also would be of interest to monitor for the unexpected transfer of the construct to nonvector *Anopheles* outside the *An. gambiae* species complex or a few other sympatric mosquito species as a measure of the specificity of the technology.

If surveillance for broader effects on biodiversity is determined to be required, then plans must be put in place for what should be assessed, how and by whom the assessment will be conducted, how the data will be collected and analyzed, and how the process will be funded. It will be imperative to agree in advance on the endpoints of concern. These should be chosen for biological relevance, as indicators not just of change per se but of potential to cause harm to the environment as defined on the national or regional level, and, given the complexity of determining causality, with serious consideration of how the resulting data will be used. The working group suggested that any such monitoring should be tightly focused on selected organisms closely related to *An. gambiae* in the ecological food web (first-order organisms as described in Table 2).

The decision regarding which nontarget species to monitor must be informed by cultural and scientific considerations and, therefore, should be determined by representatives from those regions that would be affected, positively or negatively, by implementation of gene drive mosquitoes for malaria control. There may need to be an agreement on establishment of a limited number of sentinel sites, which ideally would be a subset of sites monitoring entomological efficacy so that environmental observations can be integrated with information on the presence of gene drive mosquitoes.

As opposed to ongoing surveillance for entomological and epidemiological efficacy (see *Efficacy*), environmental surveillance is outside the purview of national vector management and malaria control programs. There will be a need to assign oversight and management to some entity with appropriate authority and how this will be handled needs to be part of regional decision-making on implementation. Options for management might include formation of a coalition of regional government authorities, establishment of an authorized regional entity, or contracting to some existing organization. One example of how this has been handled is the South African National Biodiversity Institute, a governmentally established organization with the mandate to monitor the environmental impact of GMO after commercial release.^159^ It also might be possible to partner with organizations that are already engaged in collecting data on agricultural development, conservation, climate change effects, etc.

### Efficacy.

Given appropriate planning, training, and resources, much of the monitoring for entomological efficacy of gene drive mosquitoes could be performed through existing programs. The WHO has issued guidance on IVM for national and regional program managers in sub-Saharan Africa.^160^ Entomological surveillance usually is carried out by vector control personnel at national and subnational levels. World Health Organization has recommended the establishment of sentinel sites within each country, based on criteria such as disease endemicity, ecology, and accessibility. These sites will conduct collection of *Anopheles* and other insect vectors of disease. These routinely will be analyzed for vector density, insecticide susceptibility, and various parameters of disease transmission (e.g., proportion of blood-fed mosquitoes and proportion carrying malaria parasites in their salivary glands) and behavior (e.g., number of mosquitoes resting and biting indoors). As stated previously, these ongoing activities of entomological surveillance established for monitoring of other vector control methods also will provide critical information on the efficacy of gene drive mosquitoes. For example, measurement of vector density will be a key efficacy indicator for population suppression strategies, and number of parasites in the salivary glands will be an important indicator for population replacement strategies. Attention to any behavioral changes by the gene drive mosquitoes, or any changes in carriage of other routinely monitored pathogens, would also be informative.

For gene drive mosquito products, the additional measurement of whether the transgenic construct is found in collected mosquitoes will be required. If possible, researchers should include a marker in the construct that can be assayed easily by local vector control personnel, given the availability of appropriate equipment, training, and resources; otherwise, mosquitoes may need to be shipped to a centralized testing facility that has the appropriate expertise, equipment, and resources. It will be important to bring local vector control programs into the planning process early to provide them with sufficient time to prepare.

In a typical malaria control setting, data on disease incidence is obtained by passive case detection from health management information systems and collated from public and private health facility records. Some countries have established sentinel surveillance systems to monitor malaria trends.^160^ Increasingly, countries are using district health information software platforms^161^ for reporting, analysis, and dissemination of data for all health programs. With the understanding that they will reflect the totality of malaria control measures, these systems can be harnessed for ongoing monitoring of the disease impact of gene drive mosquitoes.

In areas of low transmission and in a malaria elimination context, monitoring for entomological and epidemiological efficacy will become more difficult. The limitations of current trapping and other methods for measuring malaria transmission will become more pronounced. This may be alleviated to some extent by development of better diagnostics, traps, and other tools. The WHO has published considerations for increasing the sensitivity of surveillance systems for malaria elimination.^155^ Methods of reactive case detection and focus investigation offer opportunities to understand the circumstances of rare malaria transmission, including checking for performance failure of gene drive mosquitoes. Country coordination and cooperation will be required for regional surveillance and programs such as the current Elimination 8^162^ initiative in southern Africa provide a good example of how this might be performed.

There will be a need to define assays for product performance for use in disseminated surveillance and to determine who will conduct these assays. There also will be a need to develop a system for reporting performance failure and the decision-making process related to response, including the outcome from a regulatory perspective of a failure to respond. Similar reporting and analytic requirements are necessary for management of insecticide resistance.^163^ Regional information sharing will be an important consideration and lessons may be learned from international networks that have been established to monitor for drug and insecticide resistance (The Worldwide Insecticide Resistance Network^164^; WorldWide Antimalarial Resistance Network^165^; and IR Mapper).^166^ It is expected that the WHO will play a normative role in setting standards and guidelines.

A failure in product performance can have two different causes: 1) production or deployment problems that result in a failure of gene drive mosquitoes to establish locally after delivery; or 2) selection for or evolution of resistance mechanisms that reduce the efficacy of the gene drive construct. Production problems may be identified and corrected. Deployment problems may be addressed by reapplication of the same gene drive product, taking into account a possible need to redesign the delivery protocol in response to local conditions.

As with other malaria intervention products, such as insecticides and drugs, it is expected that naturally occurring genetically based resistance to the gene drive mosquito product will appear at some point in time. Methods employing combinations of targets can be incorporated by researchers during early development of the construct to try to prolong the viable product lifetime (see **Physically confined laboratory studies**), but these cannot be expected to maintain efficacy indefinitely. Important indicators of the emergence of resistance, or another form of product performance failure, would include: increased malaria case incidence; resurgence of vector numbers, in the case of population suppression strategies, or numbers of parasites in the salivary glands, in the case of population replacement, in the presence of the genetic construct; or alternatively, an unexpected reduction in the proportion of *An. gambiae* carrying the transgenic construct.

It probably will be necessary to follow up on any initial observations of product failure by national programs with a more focused assessment of the situation, to verify the result and try to understand its cause. This follow-up assessment likely will need to be performed by a highly trained team through a central testing facility. For population replacement strategies, it will be necessary not only to test for resistance developing in the mosquitoes, but also within the parasite population.

The most desirable scenario for performance failure due to resistance is that it is picked up early by monitoring and, therefore, is limited to a small area. In this case, it might be possible to eliminate the resistant variant locally by intensive application of traditional vector control methods and, thereby, retain the overall viability of the gene drive product. However, if gene drive mosquitoes spread as efficiently as predicted, this would be highly unlikely. Thus, planning should focus on the more probable scenario in which a rapidly and widely spreading resistant variant imminently threatens the utility of the product. Assuming malaria transmission is still ongoing at the time efficacy begins to wane, this scenario may require release of a second-generation gene drive product to maintain the effect until malaria elimination has been achieved. Lessons learned from management of insecticide resistance suggest that additional products should be ready for deployment relatively quickly after the first gene drive product is implemented.^163^ Indeed, concurrent release of multiple gene drive products might be a desirable deployment strategy for rapid effectiveness, but this would require parallel development.

The WHO likely will play an important role in providing guidance for when it is time to implement a second-generation gene drive product.

### Engagement.

Engagement in the implementation and post-implementation phases primarily will be at the national level, in the context of public health campaigns. However, regional country coordination and cooperation will be important in preparation for implementation of gene drive mosquito products. During product launch, it will be important for all stakeholders at the national or regional level to work together to make the announcement. Spokespersons should come from involved government authorities, such as the NBA and National Malaria Control Program, with product developers available to provide support if necessary.

At the implementation phase, a major concern will be obtaining financial support for wide-scale deployment and follow-on activities. Assuming that deployment will be managed through national control programs, countries will need to engage in advance with major funders such as the Global Fund to Fight AIDS, Tuberculosis, and Malaria; various national development agencies; and the regional development bank. It will be essential that these organizations understand the gene drive technology, and are fully briefed on its utility as determined from efficacy trials and on its role in malaria elimination and eradication.

### Key points: post-implementation surveillance, monitoring, and evaluation.

- After a decision to implement gene drive mosquitoes has been taken, there will be a need for ongoing surveillance, monitoring, and evaluation. The requirements for post-implementation monitoring and evaluation of gene drive mosquitoes must be considered on the background of current activities routinely conducted by national programs to monitor for malaria cases and the efficacy of vector and other control methods and activities that must be put in place in the context of malaria elimination efforts.
- There is no uniform precedent from either GM crops or agricultural biocontrol agents on which to base recommendations for post-implementation safety monitoring. By this stage, gene drive mosquitoes already will have undergone extensive risk assessment and rigorous study in the context of prior field trials, so it must be assumed that no nonnegligible adverse effects have been detected or that benefits have been deemed to justify proceeding in the presence of appropriate mitigation measures. However, because gene drive mosquitoes are a novel genetic technology, it is likely that some degree of ongoing post-implementation monitoring of effects on nontarget organisms may be expected. The decision about what nontarget species to monitor must be made on a cultural and scientific basis and, therefore, must be determined by representatives from those regions where the technology will be used to control malaria.
- If continued ecosystem monitoring is required by regulators, it will be important for parties to agree in advance on the endpoints of concern, keeping both biological significance and feasibility in mind. Effects on mosquito community structure and on organisms closely related to *An. gambiae* in the food web are logical possibilities. Prior agreement must be reached on who will conduct this monitoring and how it will be funded.
- There is guidance available for conduct of entomological and epidemiological surveillance in the context of malaria control and elimination, and these activities will be required regardless of the tools that are used. An additional efficacy monitoring requirement specific to gene drive mosquitoes will be the need to test for the presence of the transgenic construct in mosquitoes collected for entomological surveillance. Given appropriate training and resources, much of the monitoring for entomological efficacy of gene drive mosquitoes could be performed through existing programs. It will be important to bring these programs into the planning process early to provide them with sufficient time to prepare.
- As with other malaria intervention products, such as insecticides and drugs, it is expected that resistance to the gene drive mosquito product eventually will appear. If malaria transmission remains a threat at that time, evidence of product performance failure must be followed up by a more focused investigation of the cause. There will be a need to define assays for performance failure for use in disseminated surveillance and to determine who will perform these assays and analyze the results. Some may be within the purview of existing vector management programs, but there may be a need to enable centralized testing facilities for certain more in-depth analyses. There also will be a need to develop a system for reporting performance failure and decision-making on follow-up. It is expected that the WHO will play a normative role in setting standards and guidelines.
- Engagement during implementation and post-implementation primarily will be at the national level, in the context of public health campaigns. However, it will be important to ensure that funders of national control programs are fully briefed on gene drive technology and its role in malaria elimination and eradication to obtain support for implementation and post-implementation activities.

## DISCUSSION

These recommendations describe the development pathway for a new and potentially powerful tool, based on gene drive technology, to prevent transmission of vector-borne diseases. New synthetic gene drive applications, such as those using the CRISPR/Cas system, have the potential to introduce a beneficial modification rapidly into the local population of vector mosquitoes allowing it to become established, spread, and persist. Two applications of gene drive, leading to either a reduction in numbers of vector mosquitoes or a reduction in their ability to transmit pathogens, currently are being considered. Because of the present uncertainties around gene drive technology, the complexity of anticipating the development pathway was such that this working group chose to simplify discussions by focusing on a relatively narrow use, namely a tool for controlling malaria transmission by *An. gambiae* s.l. mosquitoes in Africa. However, it is expected that these recommendations will stimulate similar thinking about other possible uses of gene drive technology.

The working group members found many similarities to, and distinctions from, the development pathway for other types of vector control tools.^15,137^ The group proposed that the testing pathway for gene drive mosquitoes should follow a similar trajectory for proof of public health impact, moving from testing for entomological efficacy to epidemiological efficacy. However, the potential of gene drive mosquitoes to establish and spread within the local environment in a way that would be difficult to halt, draws parallels to the testing pathway for agricultural biocontrol agents. Thus, a key conclusion was the requirement for extensive safety testing while still working in strict containment under conditions where the probability of inadvertent environmental exposure is negligible. The working group concluded that no investigational gene drive product that reveals a potential for significant negative impact on the environment or human health during testing in containment, as compared with wild-type *An. gambiae* and conventional vector control tools, should be moved to field testing. For those investigational products that pass this initial hurdle, the pathway for further field-testing will resemble that for GM crops, initially aiming to limit environmental exposure through physical and geographic confinement and only gradually moving to open releases. A similar progression was recommended in the WHO Guidance Framework.^15^

Because of the presumed ability of gene drive products to persist in the environment, the working group recognized that development might not take place in a series of distinct and separate phases, as for other types of products, but rather that each new phase may represent an extension or expansion of the one before. Emphasis was placed on a requirement for external all-hazards risk assessment before expansion from each phase of testing to the next. Such a risk assessment may well identify issues that must be addressed before moving forward, to reduce risk and increase acceptability of testing.

The working group concluded that low-threshold gene drive products for control of malaria transmission can be tested in a safe and ethical manner. Members found many precedents that can be built on to create a pathway for responsible development of these products. These include not only existing regulatory frameworks, but also development activities being put in place for malaria elimination efforts more generally. However, they recognized that gene drive evaluation will require significant advanced planning and coordination among researchers, funders, regulators, and other government officials, and policy makers. Several important resources and practices should be put in place to prepare for field testing (see Box 9).

Box 9 **Resources recommended to prepare for field testing of gene drive mosquitoes**

More sensitive monitoring tools and approaches for detecting escapes from confinement and assessing the spread of gene drive mosquitoes in field trial

Standards and best practices for the operation of laboratories/insectaries housing gene drive mosquitoes, including an external process for validating compliance (although good laboratory practice certification is not considered necessary)

Processes and venues (e.g., databases) to promote information and data sharing

Support for appropriate risk assessment

Support for effective stakeholder and community engagement

Opportunities for regulators to obtain training and experience to build capacity in regulation of modified insects

Mechanisms for regional harmonization of regulatory requirements and regional decision-making about regulatory authorization

Quality standards for manufacturing of living mosquito products that are consistent with their production requirements

Mechanisms for oversight and management of post-implementation environmental surveillance, if required

Assays for product performance failure for use in disseminated surveillance

Systems for reporting performance failure and decision-making on follow-up

A neutral body to manage high-level coordination among the various stakeholders and organize centralized responses to the diverse challenges that will arise in the development pathway for gene drive mosquitoes as public health tools

In particular, this new technology will require a robust and transparent mechanism for regional regulatory coordination and decision-making to deal with the potential for transboundary movement. This must be underpinned by substantive capacity building, to enable decision-makers in affected countries to make well-reasoned judgments about the risks and benefits of the technology in their own context. Scientists and research institutions in the countries where the gene drive mosquito product will be used must play a central role in the development process from its early stages; it is they who will represent the technology to communities, regulators, government authorities, and other stakeholders. Emphasis must be placed not only on technology transfer to partner institutions, but on building knowledge about gene drive technology among African scientists and the public more broadly. Public acceptability is a critical determining factor in the success of this technology, and will be influenced by public confidence in the in-country scientists and government authorities to make wise judgments. Product developers (researchers and funders) bear major responsibility for conducting conscientious stakeholder engagement for their activities, listening and responding to concerns and obtaining appropriate authorizations for their work. Honest dialog between developers and involved communities is fundamental for building trust, and such collaboration must begin early. Communications about the technology and product(s) must be open and honest, avoiding hyperbole about either benefits or risks, and framed to suit the backgrounds and interests of different audiences.

Although at the time of this writing there is no gene drive mosquito product ready to enter field testing, there is substantial enthusiasm for the technology’s potential to make a significant contribution to malaria elimination and eventual eradication. Investigational products are already in laboratory development and, given the rapid pace of advances in the science, more can be expected to appear shortly. Therefore, it is imperative that testing of such products be informed by the best practices described in this report. Like its predecessor document, the WHO Guidance Framework for testing GMM,^15^ these recommendations aim to foster quality, consistency, and credibility of the processes for testing and regulating this new genetic biocontrol technology. The ability of proponents of gene drive technology to demonstrate convincingly a thoughtful and prudent approach to their work may be a key determinant of whether it achieves sufficient public acceptance to allow its potential to be tested.

## GLOSSARY

**Allele:** A variant form of a gene at a particular locus on a chromosome. Different alleles produce variation in inherited characteristics. Examples: A variant allele may cause mosquitoes to become resistant to insecticides. Other variant alleles may cause resistance to endonucleases used in gene drive technology (see Crispr/Cas9).
**Biosafety:** Policies and practices intended to prevent harm to the health or safety of human beings, other living organisms, or the environment, especially those pertaining to safe handling and containment of infectious agents.
**Biosafety committee:** A group, chartered by an institution, which is responsible for implementing policies and guidelines related to use of potentially hazardous biological agents, including, but not limited to, infectious agents, human materials, and recombinant DNA studies. This group ensures that research involving these agents does not endanger researchers, laboratory workers, human research subjects, the public, or the environment.
**Cage testing:** See Semi-field release.
**Capacity building:** The provision and promotion of education and practical training, particularly within low-resource and unskilled communities, often with respect to essential services. The term is also used in regard to promoting skills and knowledge of researchers and regulators.
**Cartagena protocol on biosafety to the CBD:** An international agreement that addresses the safe handling, transport, and use of living modified organisms resulting from modern biotechnology, with the aim of protecting biological diversity and human health. One hundred and seventy countries are signatories to the agreement, which took effect on September 11, 2003.^82^
**Champion:** An individual who plays a dominant role overcoming technical and organizational obstacles in product development.^167^
**CRTs:** Trials that group individuals into clusters, such as residents of particular villages or urban neighborhoods. Each cluster is assigned randomly an experimental treatment such as a placebo or drug, or, in the case of GMM, releases may be in one set of clusters and not in another.
**Combination therapy approach:** See Multiplexing.
**Community:** A group of people who live in or near a potential field trial or release site and have a tangible and immediate interest in the gene drive project.
**Community engagement:** Practices undertaken to inform stakeholders about the diseases and vectors of interest and goals of a proposed research study or intervention trial, and to understand stakeholder perspectives and reaction.
**Compliance:** The act of following or obeying a rule or order, particularly with respect to governmental regulation.
**Computational modeling:** The process of using various mathematical structures to predict real world situations.
**Construct:** The DNA introduced in the process of genetic engineering.
**Confinement** (also called physically confined): The use of measures that seek to prevent unplanned or uncontrolled release of the transgenic organism into the environment. For GMM, this may involve physical confinement (also termed “containment”) behind barriers within a laboratory, insectary or cage facility; and/or ecological confinement by geographic/spatial, and/or climatic isolation. In addition or alternatively, biological confinement aims to use molecular or reproductive strategies to prevent spread of the transgenic construct if escapes occur.^85^
**Containment:** See Physical confinement.
**CRISPR:** A naturally occurring mechanisms of immunity to viruses found in bacteria that involves identification and degradation of foreign DNA.
**CRISPR/Cas9:** A gene editing platform in which an endonuclease and a guide RNA are used to introduce double strand breaks at a specified location within the genome.
**DSMB:** An independent advisory group that monitors participant safety and treatment efficacy during a trial.
**Deployment:** Implementation of an intervention method to prevent disease; for example, the use of GMM as part of a national or regional program for vector control.
**Drive:** See Gene drive.
**Driving transgene:** See Gene drive.
**Ecological confinement:** A situation in which the spread of organisms is limited by the presence of ecologically unsuitable terrestrial habitat that the species cannot colonize.
**Ecological risk assessment:** The study and use of probabilistic decision-making tools to evaluate the likely benefits and harms of a proposed activity on the well-being of humans and environment, often under conditions of uncertainty.
**Ecosystem:** A dynamic biological system consisting of all organisms in a specific environment and the nonliving features of the environment with which they interact.
**Effect:** A potential beneficial or harmful outcome.
**Endemic:** A situation in which disease is present continuously at some level in an area.
**Endpoint:** An event or outcome that can be measured objectively to determine whether the intervention being studied has the desired effect.
**Entomological efficacy:** A measurement of the intended functional effect on the target mosquito population, such as reduced reproduction or competence to transmit a pathogen.
**Engagement:** Seeking and facilitating the sharing and exchange of knowledge, perspectives, and preferences between, or among, groups who often have differences in expertise, power, and values.
**Ethics:** An activity or inquiry intended to shed light on the correctness or justifiability of a given course of conduct.
**Ethics committee** (also called institutional ethics committee, institutional review board, or ethical review board): A group charged with providing oversight for biomedical and behavioral research involving humans, with the aim to protect the rights and welfare of research subjects.
**Ethical review board:** See Ethics committee
**Field trial:** An experiment designed to test a promising new investigational product or process in a context similar to that in which the product or process is intended to be used.
**First-in-class:** The first of a particular mechanism, approach, or strategy.
**Fitness:** Description of the ability to both survive and reproduce and is equal to the long-term average contribution to the gene pool by individuals having a particular genotype or phenotype. If differences between alleles of a given gene affect fitness, then the frequencies of the alleles will change over generations, the alleles with higher fitness become more common.
**Fixation:** Hundred percent frequency of an allele in gene across the species population.
**Gene:** A segment of DNA that serves as a basic unit of heredity and that serves as the chemical information required by cells for synthesis of a product.
**Gene drive:** A system of biased inheritance in which the ability of a genetic element to pass from a parent to its offspring through sexual reproduction is enhanced. Thus, the result of a gene drive is the preferential increase of a specific genotype that determines a specific phenotype from one generation to the next, and potentially throughout a population.
**Gene drive candidate:** Either the gene drive mosquito or the DNA construct.
**Gene drive mosquitoes:** See Investigational product.
**Gene drive strains:** See Investigational product.
**Gene editing:** A technique that allows researchers to alter the DNA of organisms to insert, delete, or modify a gene or gene sequences to silence, enhance, or otherwise change an organism’s specific genetic characteristics.
**Gene flow:** The transfer of genetic information from one population into another population (also called gene migration).
**Gene pool:** The collection of genes in an interbreeding population.
**Genetic engineering:** Introduction of DNA, RNA, or proteins manipulated by humans to effect a change in an organism’s genome.
**Genetically engineered mosquitoes:** See GMM.
**GM:** An organism whose genotype has been altered, including alteration by genetic engineering and nongenetic engineering methods.
**GMM** (also called genetically engineered mosquitoes, transgenic mosquitoes, or living modified mosquitoes): Mosquitoes that have heritable traits derived through use of recombinant DNA technology, which alter the strain, line, or colony in a manner usually intended to result in reduction of the transmission of mosquito-borne human diseases—see also GMO. Genetically modified mosquitoes likely also will be characterized by introduced heritable marker traits to facilitate monitoring on release into the environment, and in some cases may include only such markers, as for population biology studies.
**GMO:** Any organism that has in its genome novel DNA of endogenous, exogenous, or mixed origin that was made using modern recombinant DNA technology. Although successive selective breeding of strains of organisms with naturally occurring allelic variations also results in strains with genotypes different from the natural population, these are excluded from this definition.
**Genome:** The complete sequence of DNA in an organism.
**Genome editing:** Specific modification of an organisms’ DNA to create mutations or introduce new alleles or new genes.
**Genotype:** An individual’s genetic identity.
**GLP:** Refers to a quality system of management controls for research laboratories and organizations to ensure the uniformity, consistency, reliability, reproducibility, quality, and integrity of chemical (including pharmaceuticals) nonclinical safety tests.
**Governance:** The process of exercising oversight through traditions (standards of practice) or regulations by which individuals and communities are held accountable. Governance often involves such policy tools as professional standards of practice and codes of conduct; formal guidelines, agreements, and treaties; and legislation or other governmental regulation.
**Hazard:** An event, activity or other cause of a negative consequence or impact identified in a risk analysis.
**Horizontal gene transfer:** Heritable transfer of a functional genetic element from one organism to another without mating, most often relating to genetic exchange between different species or more distantly related species.
**Incidence of infection:** The rate at which new infections occur during the specific period of time.
**In-country:** As used in these recommendations, refers to people or groups of people, processes, and/or regions in Sub-Saharan Africa.
**Informed consent:** The process intended to ensure that human subjects who will be observed or involved in a research activity are fully and explicitly advised of all risks, costs, or inconveniences they may bear as a result of participating as a research subject, and voluntarily agree to accept or bear those risks and costs.
**IVM:** A decision-making process for the effective and efficient use of a combination of available resources in the management of vector populations, so as to reduce or interrupt transmission of vector-borne diseases.^148^
**Investigational gene drive product:** See Investigational product.
**Investigational product:** As used in these recommendations, the investigational product is considered to be the transgenic mosquito species carrying a gene drive system (for convenience, also referred to as a gene drive mosquito). This term applies to the original mosquito species (e.g. *An. gambiae* s.s.) in which the gene drive construct was introduced.
**Last mile:** The final step in the process of malaria eradication.
**Malaria elimination:** Interruption of local transmission (reduction to zero incidence of indigenous cases) of a specified malaria parasite in a defined geographical area as a result of deliberate activities. Continued measures to prevent reestablishment of transmission are required.^168^
**Malaria eradication:** Permanent reduction to zero of the worldwide incidence of infection caused by human malaria parasites as a result of deliberate activities. Interventions are no longer required once eradication has been achieved.^167^
**Mathematical modeling:** See Computational modeling.
**Migration:** The movement, often seasonal, of populations, groups, or of individuals across geographic space.
**Mitigation:** Actions, policies, and programs that serve to prevent, minimize, or compensate for disruption of the natural environment.
**Multiplexing:** Construct(s) aimed against multiple target genes and multiple sequences within each target gene.
**Nontarget effect:** A direct, unintended, short- or long-term consequence for one or more organisms other than the organism intended to be affected by an action or intervention. Concern about nontarget effects typically centers around unforeseen harms to other species, but nontarget effects can also be neutral or beneficial.
**Nontarget organism:** any organism that is not a direct target of an intended intervention. For GMM the direct target organism is other mosquitoes of the same species in the wild population.
**Opt-out:** A consent model where participants are contacted without specifically volunteering to take part in the research and excluded only when they say they are unwilling to participate.
**Pathogen:** A biological agent, such as a virus, bacterium, or parasite, that causes disease. In malaria infection, the pathogen is a unicellular parasite.
**PCR:** A laboratory technique used to amplify, make multiple copies of, a specific DNA target from a mixture of DNA molecules.
**Phased testing pathway:** A step-wise approach to guide the preparation for and conduct of research in the laboratory through environmental release.
**Phenotype:** The observable traits of an organism (i.e. how an organism appears outwardly and physiologically) based on genetic and environmental influences.
**Physical confinement:** The use of human-made or natural physical restrictions or barriers to prevent unintended or uncontrolled release of an organism into the environment.
**Population:** All individuals of a given species within a defined ecological area.
**Population biology:** The study of populations, including their natural history, size, migration, evolution, and extinction.
**Population replacement:** The use of genetic methods to change specific traits in an entire population. Also referred to population modification and population alteration.
**Population suppression:** Intentional reduction of the number or distribution of a population through physical, chemical, or biological means, particularly with pest species (also called population reduction).
**Prevalence of infection:** the frequency of infection within a population at any given time.
**Product:** See Investigational product.
**Publics:** Groups who lack the direct connection to a project that stakeholders and communities have but nonetheless have interests, concerns, hopes, fears, and values that can contribute to decision-making.
**Refractoriness:** A condition in which the mosquito is intrinsically unable to support the development of a pathogen to an infective stage or to a point of sufficient abundance such that the mosquito cannot transmit disease.
**Regulation:** an official rule to manage the conduct of those to whom it applies, usually developed from legal interpretations of legislation and implemented by government ministries or agencies.
**Regulatory agency** (also called regulatory authority, ministry, regulatory body, or regulator): A public authority or government entity responsible for exercising authority over some area of activity in a supervisory capacity.
**Remediation:** Actions, policies, and programs that seek to stop or reverse any disruption of the natural environment.
**Risk:** An objective measure of the product of the likelihood and consequences of a hazard, defined within a prescribed set of circumstances. Risk is often described as a probability distribution of a set of consequences over a defined time period.
**Risk assessment:** The process by which all available evidence on the probability of effects is collected, evaluated, and interpreted to estimate the probability of the sum total of effects.
**Risk communication:** The process through which concerns about and tolerance of risk are articulated by stakeholders and the results of risk assessment and risk management are communicated to decision-makers and the public.
**Risk management:** The process of identifying and implementing measures expected to reduce risk to a tolerable level.
**Self-limiting:** Genetically modified mosquitoes approaches where the genetic modification will not pass on indefinitely through subsequent generations.
**Self-sustaining** (also called self-propagating): Genetically modified mosquitoes approaches where the heritable modification is spread and maintained indefinitely through the target population.
**Stakeholder:** A person with a professional or personal interest sufficient to justify engagement, but may not have geographic proximity to a potential release site for a gene drive technology.
**SOPs:** Written, step-wise instructions or descriptions of essential, routine practices, intended to ensure consistent and safe performance.
**Step-wedge trial:** A form of randomized controlled trial where an intervention is rolled out in a random but sequential manner so that all control clusters eventually are exposed to the intervention.
**TPP:** A strategic development process tool that uses set of criteria to predefine ideal attributes of a candidate investigational product and subsequent modifications to acceptance thresholds.
**Trait:** A genetically determined characteristic or condition.
**Transboundary movement:** Movement across national, state, or other political lines of demarcation.
**Transgene:** Any gene transferred into an organism by genetic engineering.
**Transgenic mosquitoes:** See GMM.
**Transgenic construct:** See Construct.
**Transgenic organism:** An organism into which one or more genetic sequences from another species or synthetic sequences have been introduced into its genome by genetic engineering.
**Values:** Deeply held, complicated, sometimes evolving beliefs about what kinds of things—in humans’ lives and the world at large—should be fostered, protected, or avoided.
**Vector:** An organism that spreads disease to other species by transmitting one or more pathogens rather than causing infection itself.
**Vector mosquitoes:** Those mosquitoes that are able to transmit a disease-causing pathogen.
**Wolbachia:** A type of intracellular symbiont bacteria found in the cells of many invertebrates, including insects and nematodes, that can affect the reproductive biology of its hosts.

## LIST OF ACRONYMS

**ACL:** Arthropod Containment Level
**CDISC:** Clinical Data Interchange Standards Consortium
**CRISPR:** Clustered regularly-interspaced short palindromic repeats
**CRT:** Cluster randomized controlled trial
**DNA:** Deoxyribonucleic acid
**DSMB:** Data and safety monitoring board
**GLP:** Good Laboratory Practice
**GM:** Genetically modified
**GMM:** Genetically modified mosquitoes
**GMO:** Genetically modified organism
**IVM:** Integrated vector management
**NASEM:** National Academies of Science, Engineering and Medicine
**NBA:** National Biosafety Authority
**NEPAD:** New Partnership for Africa’s Development
**PCR:** Polymerase chain reaction
**RNA:** Ribonucleic acid
**SOPs:** Standard operating procedures
**TPP:** Target Product Profile
**VCAG:** Vector Control Advisory Group
**WHO:** World Health Organization

## Footnotes

‡ The term “threshold” refers to the proportion of modified mosquitoes with respect to the total mosquito population that will reliably initiate spread of the modification to high levels within the local mosquito population by mating. The goal of gene drive is to rapidly increase the proportion of vector mosquitoes carrying the beneficial modification. Low-threshold gene drives are defined here to include those that are predicted to spread from a rare introduction (zero threshold) or low initial release frequency.

§ Population suppression is sometimes called population reduction. Population replacement is sometimes termed population modification, population alteration, population transformation, or population conversion. The present article retains the terminology that was used in the WHO Guidance Framework.

‖ The working group acknowledged that self-limiting or self-exhausting drive approaches might be useful in restricted locations, for other diseases or under other transmission conditions, and recognized the importance of modeling for making this determination.

¶ It is possible that candidates may be imported in the form of a modified mosquito strain, most likely as eggs, to be interbred with local mosquitoes or as a DNA construct to be introduced into local mosquitoes by transfection.

# This phase of testing is herein termed physical confinement, in keeping with the terminology of the WHO Guidance Framework,^14^ but is also widely known as containment. Recommendations are based on requirements recognized as Arthropod Containment Level (ACL), and the physical structure is standardly called a containment facility.

** Although containment of *Plasmodium*-infected gene drive mosquitoes is not anticipated, Biosafety Level-2 practices are recommended for this parasite.^108^
